# Factors influencing tuberculosis preventive treatment implementation in India: A hybrid inductive-abductive thematic analysis using implementation framework

**DOI:** 10.1371/journal.pone.0359171

**Published:** 2026-10-14

**Authors:** Prakash Prabhakarrao Doke, Amruta Paresh Chutke, Jayashree Sachin Gothankar, Sanjivani Vishwanath Patil, Medha Deepak Bargaje, Bhagyashree Mitragotri, Dipali Pakhale

**Affiliations:** 1 Community Medicine, Bharati Vidyapeeth Deemed University, Pune, Maharashtra, India; 2 Central Research and Publication Unit, Bharati Vidyapeeth Deemed University, Medical College Hospital, Pune, Maharashtra, India; 3 Department of Research and Biostatistics, Bharati Vidyapeeth Deemed University, Homeopathic Medical College, Pune, Maharashtra, India; 4 Respiratory Medicine, Bharati Vidyapeeth Deemed University, Pune, Maharashtra, India; Independent Global Health Consultant, INDIA

## Abstract

**Background:**

India has committed to achieving ‘Eliminate Tuberculosis (TB)’ by 2025. The Government of India issued guidelines regarding the crucial strategy of Tuberculosis Preventive Treatment in July 2021.

**Objectives:**

To identify factors influencing tuberculosis preventive treatment program implementation and acceptance of tuberculosis preventive treatment.

**Methods:**

We conducted the study in the Pune district from April to August 2024. It was a qualitative study using Focus Group Discussions (FGD) with Medical Officers (MO) and adult contacts of the index case, and In-depth Interviews (IDI) with senior health officers. We conducted eight FGDs with MOs and six with adult contacts, and conducted in-depth interviews with senior health officers. Data were transcribed, translated, and analyzed using MAXQDA.

**Results:**

Eighty-six health/medical officers and 43 adult contacts participated in FGD and IDI. Some health officers have received training in tuberculosis but not in preventive treatment. Knowledge about tuberculosis preventive treatment and duration was minimal. Medical officers minimally briefed contacts. Stigma mainly from contacts perspectives, migration from health personnels perspectives and lack of awareness from both the groups emerged as important factors influencing screening and treatment of adult contacts. Very few underwent blood/ X-ray/ sputum testing, and fewer received treatment for varied durations. Medical officers knew only the isoniazid regimen, and interferon-gamma release assay testing was either unknown or unavailable. There is a shortage of trained human resources. The supply of diagnostic kits and medicines was disrupted. Health personnel and contacts recommended non-pharmacological measures such as mask use, cleanliness, and good nutrition. All officers perceived that elimination may linger until 2030. Training, counselling, community awareness, and multi-sectoral collaboration were strategies suggested by health officers.

**Conclusions:**

Health personnel capacity building, strengthening infrastructure and logistics, multiple strategies to mitigate stigma, and multi-sectoral collaboration are needed to satisfactory implementation of TB preventive treatment.

## Introduction

Globally, Tuberculosis (TB) has remained a crucial public health problem. The recent meta-analysis studies and the nationwide survey conducted by Indian Council of Medical Research have estimated a prevalence of 296–350 per 100,000 population above 15 years [1–3]. Incident cases have declined to 187 per 100,000 population in 2024 [4]. Although the cases are declining, India accounts for a disproportionately higher share (25% of cases) of TB cases compared to China [5].. The TB control program has seen a paradigm shift from control to elimination, particularly after the WHO’s End TB strategy [6,7] which is reflected in the commitment to achieving ‘Eliminate TB’ by 2025, five years before the Sustainable Development Goals targets.

The close contacts of a positive pulmonary tuberculosis case are susceptible to infection, especially the vulnerable population. Tb preventive treatment is defined as, “treatment offered to individuals who are considered to be harboring TBI (TB infection) and to be at risk of developing TB disease in order to reduce that risk. Also referred to as treatment of Latent TB Infection or TB infection, or TB preventive therapy [8].” Targeting latent TB infection is essential for elimination, as the majority of cases result from the activation of existing foci.

The Government of India issued guidelines on TPT in July 2021 [9], and updated later. The management of LTBI will accelerate elimination and support the ‘*TB Mukht Bharat Abhiyan’*. During the 2010s, the decline in incidence was approximately 2.5% annually, and the nation desires to reach about 10% annual decline, which may be achieved by upscaling TPT to people living with HIV, to all contacts, including children, and high-risk individuals of progression from LTBI to disease [9,10].

The annual India TB reports have consistently pointed out poor TPT initiation among household contacts. The latest 2024 report shows that TPT was initiated in only 26% of adult household contacts, far lower than among children [11]. The global scenario is almost similar; only about 10% of estimated household contacts complete the preventive treatment [5]. Several studies have identified challenges like poor knowledge of health workers, human resource deficit, non-integrated HIV/TB services, logistics problems, and community unawareness in the implementation of TPT [12–16]. Government of India needs to focus more on mass educational drive for Tb preventive treatment. Oral or written Government communication or personal interest in reading are the only sources to acquire knowledge and shape perceptions. The feelings towards TB elimination will be formed on the knowledge and perceptions. However, qualitative studies exploring the experiences of various stake holder including health care providers and adult contacts of the index case are limited in India. The aim of this study was to identify the reasons for the poor compliance of preventive treatment from the service providers and the beneficiaries.

### Objectives

1. To identify factors influencing tuberculosis preventive treatment program implementation among the medical officers and senior health officers from Pune district
2. To identify the factors for the acceptance of tuberculosis preventive treatment among adult contacts of patients of the index positive case

## Methodology

### Research team and reflexivity

A pair of authors conducted Focus Group Discussion (FGDs) and In-Depth Interviews (IDIs). The first four authors are medical Postgraduates, and two of them hold a Doctorate and are teaching faculty (except one, who is a member of the research unit) at a medical college. All are women except the first author. First four authors have more than a decade of experience in qualitative research. The remaining two have about five years of experience assisting with research.

The interactions between the investigators and the participants were for the first time. The participants were briefed by the concerned health/medical officers about the investigator team. Hence, the participants knew the institution and that the investigators are doctors and directly/indirectly are related to the tuberculosis elimination program. The team members, due to their background in teaching and health administration, were interested in this research project.

### Study design

This was a qualitative study using FGDs and IDIs. We conducted FGDs with medical officers and adult contacts of TB patients, and IDIs among senior concerned health officers. We used a hybrid inductive-abductive thematic analysis and did not start with any specific codes. The senior health/medical officers, on behalf of the investigators, telephonically/orally invited about ten consecutive participants who were available and willing to join FGDs. The authors telephonically requested all concerned senior health officers to spare time for IDIs.

### Sample size and sampling

The number of FGDs and IDIs were pre-determined and sampling was convenient. The study conducted one FGD for MO PHCs of two adjacent blocks (excepting one FGD which represented three blocks). We invited available on the day of FGD and willing in-charge medical officer from each PHC. We also invited medical officers working as Taluka Health Officers for focus group discussion. A total of six FGDs were conducted in the rural area. One FGD of medical officers was conducted in each of the two Municipal Corporations. In corporation area, the study invited willing MOs from nearby health institutions. We requested district level supervisory officers and higher officers from the Corporations to refrain from FGDs. There were no exclusion criteria for MOs FGDs both from rural and corporation areas. Fig 1 provides information on the blocks from which MOs attended the FGD.

**Fig 1:**
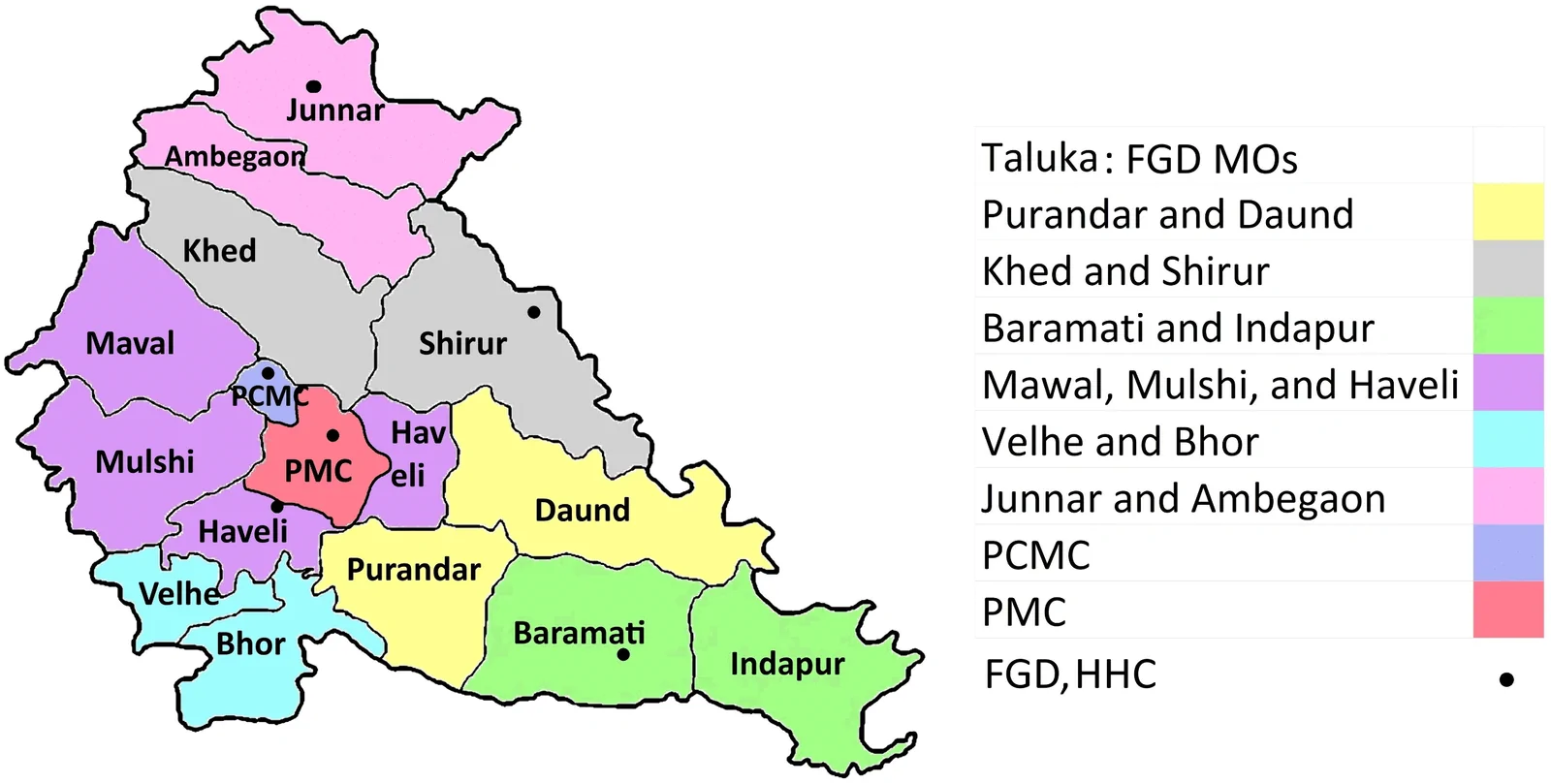
Focus Group Discussions places and area representation, Pune, India.

We randomly selected four blocks and then one PHC from each. The study invited adult contacts of a TB case residing in the PHC village to participate in an FGD. Similarly, the authors, through corporation officers, invited adult contacts of cases from Corporation areas and conducted two FGDs, one in the Pune Municipal Corporation (PMC) and one in the Pimpri Chinchwad Municipal Corporation (PCMC).

We conducted IDIs with the following officers: State TB officer, City TB officer PMC, City TB officer PCMC, District TB officer, District Health Officer, Medical Health Officer of PMC, and Medical Health Officer PCMC.

In the study, seven senior health officers, 43 adult household contacts of patients having pulmonary TB, and 79 medical officers participated. While conducting IDI, for ice breaking we asked about their performance. One senior health officer did not participate and instructed the concerned senior medical officer to grant us an interview.

### Study settings

We conducted the study in Pune district, India. The district has a population of 9.43 million. There are 13 blocks in the district. In rural areas, the Primary Health Centre (PHC) provides TB services to the population. There are two Municipal Corporations in the district, PMC and PCMC. The corporations provide TB services to the population through Hospitals, Dispensaries, Urban Health Posts, and Tuberculosis Units. The investigators conducted FGDs at participants’ workplaces (either PHCs or Taluka Health Offices) and IDIs at the offices of the concerned participants. The FGDs were conducted in the absence of any distractions and supervisory officers, and the group were homogeneous..

The FGDs and interviews were conducted from April 2024 to August 2024. Table 1 provides details on the socio-demographic characteristics of the healthcare officers. The mean age of the medical officers was 37.37 (SD: 8.92) years, and total experience was 9.38 (SD: 8.40) years, and mean experience in the TB program was 7.83 (SD: 8.11) years. All were at least graduates in modern medicine (63) or Ayurveda (22), except one, who was a technician representing a medical officer. Table 2 provides the details of the demographic characteristics of the adult contacts. The mean age of the adult contacts was 38.47 (SD: 13.73) years. Only and 29 persons responded to duration of education. The mean duration of school education was 8.97 (SD: 4.76) years.

**Table 1:** Demographic details of healthcare providers participated in focus group discussion or in-depth interviews in Pune district.

FGD/IDI	No. of participants	Age in years Mean (SD)	Sex: Male n (%)	Experience in years Mean (SD)	Experience in TB program in years Mean (SD)
FGD MO 1	12	36.17 (8.50)	7 (58.33)	10.50 (9.54)	9.86 (9.31)
FGD MO 2	9	37.89 (9.05)	8 (88.89)	6.22 (5.12)	5.89 (5.42)
FGD MO 3	14	37.93 (9.01)	8 (57.14)	11.14 (9.21)	10.36 (8.98)
FGD MO 4	8	33.13 (8.71)	5 (62.50)	4.63 (4.49)	4.51 (4.55)
FGD MO 5	10	34.00 (9.18)	4 (40.00)	7.78 (8.91)	7.89 (8.98)
FGD MO 6	8	39.88 (10.64)	4 (50.00)	14.14 (9.86)	13.14 (10.38)
FGD MO 7	9	39 (10.38)	5 (55.56)	11.61 (10.15)	3.61 (7.16)
FGD MO 8	9	39.78 (6.78)	4 (44.44)	7.89 (5.37)	5.33 (4.42)
IDI	7	47.29 (7.11)	6 (85.71)	19.29 (9.45)	8.27 (7.45)

**Table 2:** Demographic details of adult contacts of index case participated in focus group discussion in Pune district.

FGD	No. of participants	Age in years Mean (SD)	Sex Male n (%)	Years of schooling Mean (SD), N=29
FGD AC 1	9	47.89 (15.24)	6 (66.67)	6.11 (5.71)
FGD AC 2	6	41.17 (17.34)	4 (66.67)	9 (3.35)
FGD AC 3	10	32.3 (10.35)	5 (50.00)	10.10 (3.35)
FGD AC 4	6	34.83 (14.27)	2 (33.33)	3.67 (5.47)
FGD AC 5	6	38.50 (6.47)	2 (33.33)	3.67 (6.83)
FGD AC 6	6	35.5 (14.27)	2 (33.33)	2.33 (5.72)

### Data collection

The study collected data using a pretested, validated interview guide. We did not repeat discussions/interviews. The discussions and interviews were audio-recorded. One experienced person took notes. We conducted the interviews in the local language (*Marathi*). The interviews began with information about the research project. Investigators also informed participants about confidentiality and voluntary participation and encouraged them to share their experiences freely. The FGD lasted 45–60 minutes, while the IDI lasted 30–45 minutes. At the end, the summary was shared with the participants, but the written transcript was not. Although the number of FGDs and IDIs was pre-determined, we observed data saturation. In the seventh MOs FGD a few points emerged while in the eighth FGD no additional point emerged. Similarly, in sixth IDI very few points emerged and in the seventh no new point emerged.

The study obtained approval from the Medical College Institutional Ethics Committee (BVDUMC/IEC/252). We obtained informed written consent from the participants for participation, audio-recordings, and publication.

### Data analysis and findings

The recorded interviews were transcribed and further translated in English by last two authors who had taken notes and audios. The transcripts and the translations were cross-checked against the recordings and notes taken. Data was anonymized and analyzed. A hybrid inductive-abductive approach to thematic analysis was adopted. Initially, the codes were identified using inductive analysis mostly by one author supported by another and reviewed by the senior author. The codes were then grouped based on the Consolidated Framework for Implementation Research (CFIR) using abductive approach. The researchers reviewed codes, and finalized the code tree. MAXQDA software version 22.8.0 was used for analysis. We did not study the variability. Excerpts representing the identified codes were reported with participant number. The present study used the Consolidated Framework for Implementation Research (CFIR) for analysis. Fig 2 provides the details of identified codes.

**Fig 2:**
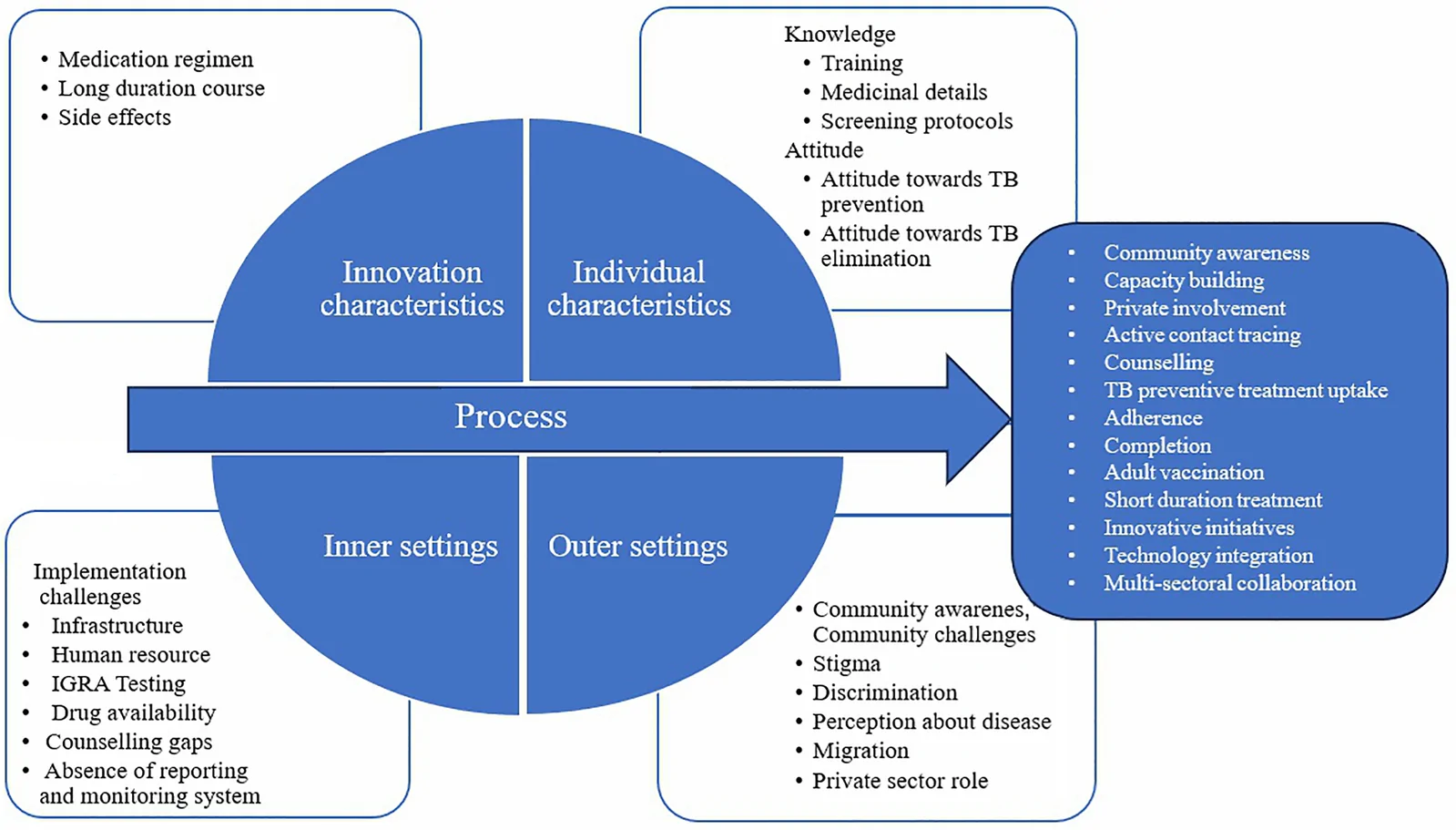
Identified codes based on Consolidated Framework for Implementation Research (CFIR) in Pune district, India.

### Innovation characteristics

The characteristics of the TPT intervention itself influenced its acceptability and implementation. Health personnel participants perceived TPT as beneficial in reducing future risk of tuberculosis.

FGD AC5 R3: “Chest X-ray was done, and reports were normal. But for prevention, the pills were started. She and her son were taking the pills only.”

However, prolonged duration, pill burden (sometimes, 100 mg INH were received instead of 300 mg) and concerns regarding adverse effects negatively affected acceptance and adherence.

A few healthcare providers correctly described TPT to be initiated after screening of the household contacts and ruling out active tuberculosis among adults; and treatment is isoniazid for six months. HCPs also reported that sometimes the drugs were available in lower strength, where the pill burden had to be higher, affecting the compliance. Few adult contacts who had received TPT reported side effects as well as variable treatment durations ranging from one to six months. This reflected the inconsistencies in implementation.

FGD MO 1 R1: “Sometimes INH is available as a 100 mg tablet, instead of 300 mg. Then people need to be given 3 tablets for a single dose. That is why compliance is reduced. The second thing is Rifapentine is not available here.”

The findings indicate that intervention characteristics affect the treatment adherence behavior of the adult contacts, thereby affecting the compliance.

### Individual characteristics

Many of the participants reported about the TB treatment for the active patients, and prophylaxis treatment among children, rather than that of the adult contacts. Many of the health care providers were not aware of the TB preventive treatment guidelines for adult contacts, including screening protocol and dosage.

FGD MO 6, R2: “We start Isoniazid therapy for high-risk contacts, i.e., for children below eight years of age, in contact with patients on TB treatment.”

Very few health care providers reported correct information about TPT. Majority of the MOs did not receive formal TPT training. The limited information they had was acquired through informal discussions and meetings.

FGD MO1, R1: “Actually, we are giving TB Preventive Therapy to close contacts of sputum-positive pulmonary TB cases. We give INH 300 mg for six months, as that is the current best available treatment. After confirming the index case, contacts are screened. Contacts are asked about symptoms and investigated with X-ray or sputum testing. If negative, we can start TPT.”

IDI 1: “I have been working in the TB program for many years. But no formal training on TPT has been provided so far.”

Knowledge of TPT was poor among adult contacts. Very few contacts underwent screening and testing, and only a few received TPT.

FGD AC 4, R-4: “When we came to the hospital and took treatment for the patient, no one told the family members that they could also suffer from TB through the patient. The information is not provided to us.”

Despite the limited knowledge, the MOs were positive towards the role of prevention strategies in tuberculosis control. However, they were skeptical about achieving the TB elimination goal by 2025.

IDI R3: “Elimination is possible though our graph of the number of cases is still increasing. After the increasing trend, there will be a plateau, and then a decline. So, it will take another 10 years.”

The adult contacts were also confident that TB would be cured and controlled upon the implementation of appropriate measures. However, they were unaware of the TB elimination goal and were unable to comment on that.

The findings suggest that insufficient knowledge at both provider and community levels influencing implementation of TPT.

### Inner settings

The challenges reported at the health facility level, were logistics-related and a lack of trained manpower. These included limited availability of diagnostic facilities, irregular availability of Cartridge-Based Nucleic Acid Amplification Test (CBNAAT) kits, unavailability of x-ray facilities at the PHC level, and limited availability of drugs.

FGD MO 3 R11: “Basically, there is no dedicated, trained staff for taking quality sputum samples and performing sputum quality checks. We have to force the existing staff, like ASHAs, to understand how to collect sputum. Often, the sputum is of poor quality, and its quality examination is not being performed.”

FGD MO 2 R8: “CBNAAT toolkit is not there. Many times, the cartridge is not available. It was not available for at least 4-5 months.”

FGD MO 3 R3: “The biggest problem is this intermittent drug supply. Drugs were not available for the last 4-6 months.”

MOs reported that counselling was provided to patients and their family members by the health staff; however, it was not universally comprehensive at all places. Additionally, the medical officers reported the absence of a monitoring and reporting system for contacts as a challenge.

The findings suggest that despite the availability of guidelines on TPT, weaknesses within the health system infrastructure and organizational readiness limit effective delivery of TPT.

### Outer settings

Social, cultural, and community-level factors strongly influenced implementation of TPT. Stigma emerged as the most prominent barrier consistently reported across all stakeholder groups but more conspicuously among adult contacts. It was also reported to be more pronounced in the rural and tribal areas. Adult contacts reported experiences of discrimination, social isolation, fear of disclosure and emotional distress. Consequently, families would maintain confidentiality regarding TB diagnosed individuals. Family contacts were often reluctant to cooperate with sputum collection, investigations, and follow-up visits. Stigma and limited knowledge about the disease’s spread influenced the acceptance and implementation of TB treatment as well as TPT.

FGD AC 3 R4: “My experience is that after knowing about TB patient, the neighbors started looking at us in a weird way.…. we used to feel ashamed while taking our … (relative) anywhere. We came here from… village. It was difficult for me to get a room for rent initially. It was a very bad situation….”

Secondly, the patient’s close contacts reported that they were asymptomatic and healthy, and thus believed that treatment was not needed. HCPs further reported that some patients preferred seeking treatment from private practitioners or migrate, so as to avoid social labeling.

Migration was another important barrier reported, especially among migrant laborers in industrial sectors and agricultural workers with seasonal mobility patterns. This affected the implementation of the program, limiting screening, treatment initiation, and follow-up.

IDI R6: “Major drawback in case detection, treatment completion, and contact tracing is migration. People migrate in search of work. Secondly, if they are detected with TB, they migrate because of stigma and to earn a livelihood. People working on construction sites stay for a few months only and keep moving. So, the detection is low.”

The MOs also reported limited knowledge and involvement of private practitioners, resulting in delayed diagnoses or non-reporting of cases treated by them and delayed contact tracing.

The findings indicate that outer setting factors act not only as social barriers but also as influence treatment-seeking behavior, adherence, and continuity of care.

### Process

The implementation process of TPT involves screening, contact tracing, counseling, treatment initiation, and follow-up. However, multiple operational challenges affected implementation.

Healthcare providers described efforts toward household screening and counseling of patients and family members. Contact tracing was challenging, especially among migrant populations, limiting timely initiation of the preventive treatment.

FGD MO8 R6: “Most people require counselling multiple times, as they think they are not ill, so why take care and medicines? If counselling is done properly, people respond positively. Patients stay in contact with us. Patients’ relatives also come to get TPT. Regular follow-up is a must.”

FGD MO 1 R1: “Household contacts are traced. But their workplace contacts, neighborhood contacts, etc., are more important according to my opinion. We are missing these contacts.”

Both healthcare providers and adult contacts recommended strengthening community awareness programs focusing on disease prevention, treatment adherence, and curability of tuberculosis. Counseling was considered essential for improving awareness regarding disease transmission, reducing stigma, alleviating anxiety, and motivating treatment completion.

FGD MO 1R5: “The first and most important thing is awareness. TB disease and its treatment, and precautions need to be informed to people. We search for TB patients, but if we give proper treatment to all close contacts, then cases will be reduced. I mean awareness should be through education, via mass media or school teachers. This will have a benefit.”

Suggested implementation strategies included structured training programs for healthcare workers and private practitioners, expansion of initiatives such as *Axshaya Plus*, incentives for ASHAs, and involvement of TB Champions (recovered patients) and community leaders to improve awareness and reduce stigma. *Nikshaya Mitra* (Nutritional support), adoption of patients by politicians and industrialists, collaboration with community leaders and organizations in various mass programs, were other strategies suggested by the healthcare providers to address socio-cultural and community barriers.

Healthcare providers also recommended using centralized digital reporting systems to track migrating patients and improve continuity of care. Additional suggestions included workplace-based TB prevention initiatives, inter-sectoral collaboration, and strengthening community participation through programs such as *TB Mukta Panchayat and TB Mukta Bharat Abhiyan.*

IDI R4: “We have launched a new initiative, i.e., *TB Mukta Panchayat*, which means we are starting it at the grassroots level.…. There is another initiative, namely, union-supported TB-free workplaces. The union people spread awareness in the industrial area, emphasize this point, and inform about preventive measures. One more initiative is inviting TB champions. We invite TB patients for counselling, and during that session, we call cured TB patients TB champions to share their experiences. This helps patients gain the confidence to complete their treatment and get cured…..We had one agency, *Axhaya Plus*, that supported in the process of completing TPT of the contacts.”

Overall, the findings demonstrated need of strengthening of implementation processes across community, and organizational levels through community engagement, and system strengthening initiatives.

The common codes/themes that emerged during discussions and interviews are shown in Fig 3. Senior health officers believed that almost all medical officers are trained. Contrarily, most medical officers said that they did not receive the specific training. Medical officers estimated 60–75% initiation of TPT, whereas senior health officers’ estimates were higher, at 80–100%. Medical officers, through health care workers, may have provided counseling about investigations and chemoprophylaxis. However, contacts were not agreeable to counseling. They opined on the need for thorough counseling. Senior officers were revolving around policy points. Medical officers represented the program’s implementation. While the contacts shared their live experiences.

**Fig 3:**
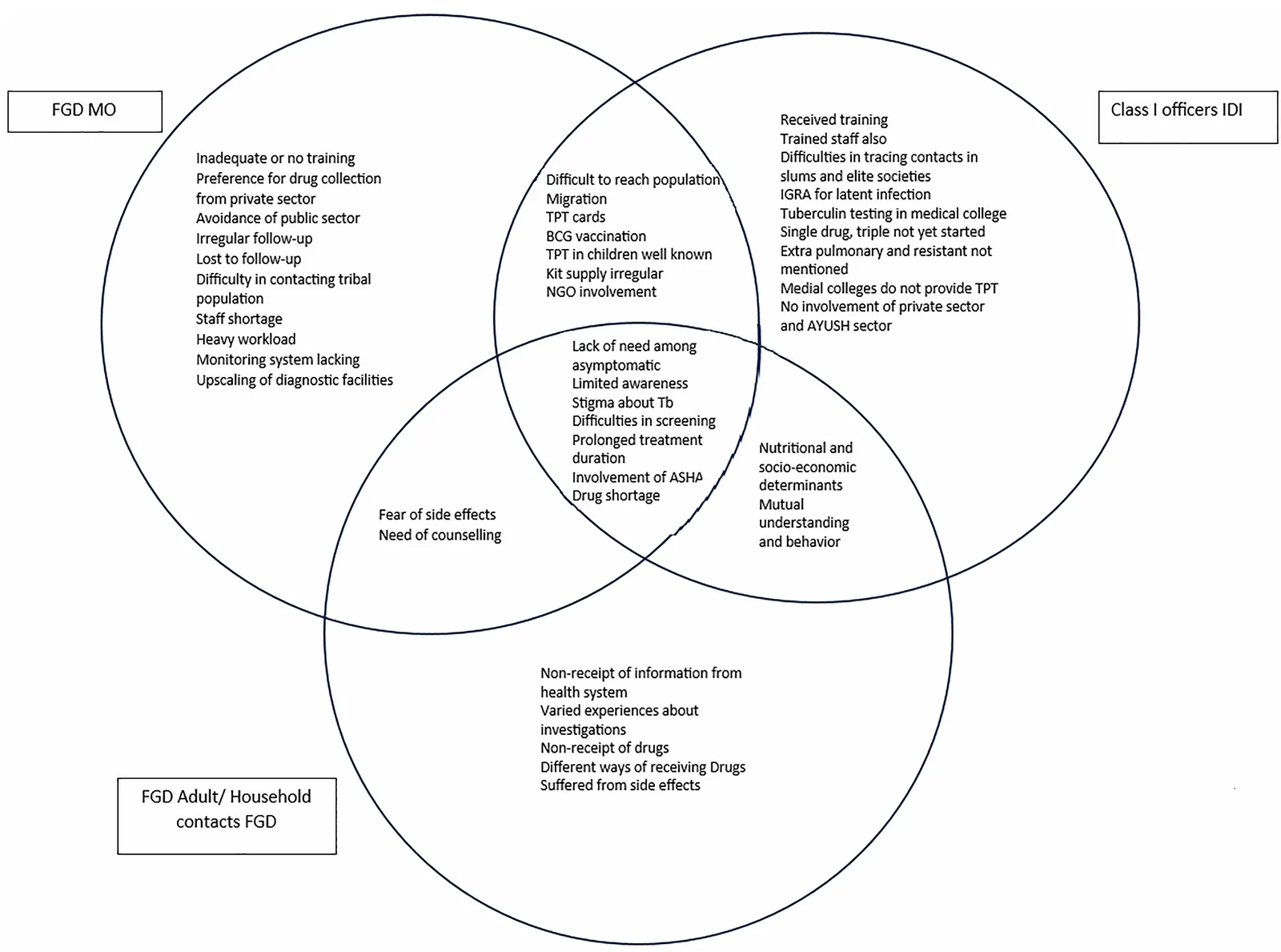
Common and unique findings from the focus group discussions and in-depth interviews in Pune district, India.

## Discussion

The study is unique which combines the providers and beneficiaries perspectives. The focus on registration, initiation, and completion monitoring is minimal. Even the annual India TB reports mention only number of initiated TPT. Lack of TPT documentation leads to assessment difficulties [17,18].

Before starting TPT for household contacts one has to rule out active TB infection and identify contacts eligible for TPT [8,19]. IGRA has been the preferred test for detecting TB infection. Many times X ray facility may be out of order, skin test, IGRA (outsourcing IGRA is costly), and drugs are not available at peripheral institutions; other barriers include absence of transportation and dedicated team [20,21]. Similar problems exist in India and outside also for implementation and monitoring [18,22–25]. Contact tracing and investigations are challenging; the person has to commute long distance first to peripheral institution then to higher institution. It is time consuming; many contacts have family problems like kids in home hence they do not comply instructions [26]. Our study also endorsed the unavailability of trained human resources, newer diagnostic test as well as drugs. As result very few contacts underwent X-ray, sputum or blood examination, or received and completed treatment.

Lack of knowledge among health care workers including medical officers is chronic, ubiquitous and pernicious. The lack pertains to all aspects of TPT, infection testing procedures like TST, IGRA, the advised drugs, duration of treatment, side effects of the drugs, efficacy of TPT, etc.[17,18,22,23,25,27]. The lack of knowledge is result of non-receipt of proper training. Non-inclusion of TPT in TB training highlights the fragmented approach of the program. Like present study in Delhi also the health care workers have not received TPT training [18]. This highlights the need of a structured training program for TPT to be incorporated in the regular trainings for medical officers and other health care workers.

Counseling should be very strong component of training. Root cause of poor acceptance and compliance is prevailing stigma associated with TB. Stigma emerged as another most important barriers to TPT uptake in our study. The limited contacts undergoing investigations and treatment may also be due to stigma. TB-related stigma significantly hinders testing, contact tracing, treatment linkage, and medication adherence [22,23,28]. Stigma acts at multiple levels as social rejection, fear of labeling, workplace discrimination, and family exclusion, further leading to delayed or avoided care-seeking behavior. A narrative review reported various types of stigmas including anticipated, perceived community, enacted and internalized stigma, that can affect mental well-being, furthermore affecting the treatment seeking behavior, treatment adherence, and treatment completion [28–30]. The participation of adult contacted in FGDs may be affected by the stigma of attending PHC. Additionally, the perception among household contacts that healthy person does not need medicines, long duration of treatment prevent acceptance of TPT [18,26]. Such barriers are reported from African country also [31]. It is already documented that if contagiousness and the minimal risk of side effects is explained and perceived, acceptance improves [32]. Addressing stigma needs continuous efforts and strategies beyond community awareness programs. Individual and community-level awareness programs alone are insufficient to address stigma. The *TB Mukt Bharat Abhiyaan* strategy emphasizes de-stigmatization through community engagement, normalization of discussions around TB, and dispelling of myths [33]. Tele counseling of contacts in the form of text messages, audio and video clips may partially address problems of attendance at institutions due to their busy schedule of and the stigma.

Many cases of TB are treated by the private practitioners. Our study cautions that private providers may not be aware of TPT guidelines, and rarely provide TPT, consistent with other studies [34–37]. There is need of involvement of private sector over and above notification. Regular CME should be conducted for private practitioners. Patients diagnosed in private clinics should be linked to National Tuberculosis Elimination Programme for appropriate action, including contact tracing and TPT [38].

Migration is one of the barriers in contact tracing and screening. It also may contribute to non-completion of the preventive treatment in many individuals along with other reasons [23,39]. The migration point appeared only in MOs FGDs. The senior health officers did not think and for contacts it was not applicable. Amongst different barriers, societal stigma about tuberculosis emerges the most important determinant of non-acceptance of preventive treatment. The persons fear that he/she may be labelled as tuberculosis patient if take treatment. The second important barrier was the individual perception that a healthy person does not need any treatment.

TB elimination would require an 80% reduction in new TB cases and 90% reduction in deaths compared to 2015 levels [33]. Considering the present situation, the MOs and senior health officers perceived that TB elimination would be delayed, probably by five years after the target, i.e., by 2030.

A study in Mumbai used home based IGRA testing and treatment strategy reached 85% treatment initiation and 90% treatment completion rate [40]. While, another implementation study used a structured “7-1-7” timeliness metric (identify household contacts within 7 days, initiate TPT within 1 day if eligible, and ensure follow-up within 7 days) through TB champions to improve household contact screening and TPT initiation [41]. The short duration isoniazid-rifapentine regimen has also been evident to be improve the completion rate [16,39,42,43]. Thus, strengthening infrastructure and availability of newer diagnostic tests, like biomarkers and newer treatment regimen are required for effective implementation [44]. Performance-based incentives to ASHAs may also be tried. In project mode the performance may be better; 90.6% completed TPT in the project [21]. Government has approved Rs.250 per person to ASHAs after completion of TPT.

The senior health government officers are transferred less frequently than medical officers. Officers from corporation are rarely transferred. This may be root the cause for some discordant findings about training. During COVID-19 pandemic other programs were ignored that may have resulted in suboptimal performance of contact tracing, screening and completion of TPT. Senior officers instructed and expected good TPT coverage but probably their opinions were optimistic. The happenings in the field were revealed by medical officers.

The study represents a fairly large population. The authors are highly qualified and experienced. The participants were care providers, decision makers, and beneficiaries. In FGDs the of Medical Officers supervisory district level officers or senior officers who may influence responses were not involved. The study provides insights into perceptions and challenges among health care providers and the adult contacts regarding TPT. The study was conducted in a single district and hence the results may not be generalizable to other settings. The Taluka Health Officers were present in the FGDs. Although they are from medical officer cadre, are working as in-charge health officer of an administrative block; hence their presence might result desired responses. We did not analyze inter-coder variability.

Further research may be conducted in different areas like tribal/hilly areas to explore the perceptions and challenges. Also, conducting mixed methods studies including assessment of TPT coverage, completion rates, and triangulation of the qualitative and quantitative findings would add to our comprehensive understanding of the problem. We recommend that the government should initiate mass educational drive for TPT and specific training of all health personnel (including private practitioners) on TPT.

## Conclusions

The study concludes that a robust monitoring system, regular supply of drugs, and transportation to contacts should be assured. Capacity building of healthcare providers for technical details and counseling is essential. Training of health workers may not be sufficient to reduce stigma; hence, community involvement, as advocated in the *TB Mukt Bharat Abhiyan,* is critical. Private sector involvement is also critical for translating TPT guidelines into practice.

## Supporting information

S1 File Focus Group Discussions (Adult contacts); Focus Group Discussions (MO); In-depth Interview. (ZIP)
