# Supplementary material for: Factors influencing tuberculosis preventive treatment implementation in India: A hybrid inductive-abductive thematic analysis using implementation framework
Source: PLoS One. 2026 Oct 14;21(10):e0359171. doi: 10.1371/journal.pone.0359171 (PMC13667825; doi:10.1371/journal.pone.0359171)
Supplement: Supplementary file 1 [file pone.0359171.s002.docx]

| Reviewer 1 | The authors thank the reviewers of constructive comments. |
| --- | --- |
| 1. Is this a basic qualitative study? Please clarify this point.  2. What are the exclusion criteria?    3. How do you reach the data saturation point? Explain briefly.  4. Do not mention any results in the Method section; instead state them clearly within the result section.  5. Write the name of the software used to process and analyze the data within the method section. 6. In the discussion section, write about the recruitment, such as why some people refuse to participate? 7. Write briefly about the potential role of telehealth in improving tuberculosis preventive treatment approach. 8. Write about the limitations of the study and any potential bias. 9. Check the written abbreviations throughout the manuscript. | 1. Yes, it is a basic qualitative study. It is clearly mentioned now. 2. All those who were available and willing were included. There were no exclusion criteria. 3. In the seventh FGD a few points emerged while in the eighth FGD no additional point emerged. Similarly, in sixth IDI very few points emerged and in the seventh no new point emerged. It is mentioned now. 4. No results/analysis is included in method section 5. We used MAXQDA software. It is already mentioned. 6. As suggested, one sentence is added in discussion. 7. A few lines are mentioned. 8. One important limitation and potential bias is mentioned now. 9. The abbreviations are standard in the Indian context. However, as per the suggestion and keeping international readers full forms are given at first appearance of abbreviations. |
| Reviewer 2 | The authors thank reviewer for critically reviewing and giving suggestions for improving quality of the manuscript. |
| I appreciate the authors for addressing an important public health issue and for employing qualitative methods such as FGDs and IDIs, with the identification of five themes being commendable. However, the manuscript requires substantial revision to improve clarity, structure, and alignment with qualitative research reporting standards. The title needs modification. While knowledge and perceptions can be directly inferred, barriers are largely subjective and context-dependent; therefore, the title should be revised to better reflect this distinction. Additionally, the key terms used in the title and objectives: knowledge, perceptions, barriers, and practices, should be clear and conceptually resolved in the introduction. The introductory section is overly detailed. The initial paragraphs describing TB incidence can be shortened and summarized, and the details of India’s elimination programs can be consolidated into a single paragraph. The study aims also need to be more clearly and precisely stated. There is noticeable repetition between the data collection and data analysis sections, which should be streamlined. The manuscript would benefit from being rewritten in accordance with established qualitative reporting guidelines (e.g., COREQ or SRQR), with clearer description of analytical methods. Although the identification of themes is appropriate, detailing every response from interviews is not necessary. The results section should be more concise, using representative quotations rather than extensive verbatim reporting, and should emphasize synthesis over description. Overall, I kindly advise the authors to thoroughly reframe and rewrite the manuscript after reviewing similar high-quality qualitative publications. As a useful reference, the following article may help guide structure and presentation: <https://jamanetwork.com/journals/jamadermatology/fullarticle/2841453> | The title is modified.  A few sentences are added in the introduction section.  The introduction section is reduced as per the suggestion.  The aim is rewritten.  The repetition is removed.  We have used COREQ guidelines.  We have used the suggested article for data analysis and findings. |
| Reviewer 3 | Many many thanks for reviewing and nicely summarizing the contents of the manuscript. |
| This qualitative study conducted in Pune district, India, explored knowledge, attitudes, and implementation barriers related to tuberculosis preventive treatment (TPT) among medical officers, senior health officials, and adult household contacts of pulmonary TB patients. Using focus group discussions and in-depth interviews, the study identified major gaps in awareness and training regarding TPT, particularly among healthcare providers and community members, along with low uptake and inconsistent implementation of preventive therapy.  Key identified barriers included stigma, migration, limited diagnostic and drug availability, weak monitoring systems, and inadequate engagement of private practitioners, while participants generally expressed positive attitudes toward TB prevention despite skepticism about achieving elimination targets.  Suggested solutions included strengthening community awareness and counselling, improving healthcare worker training, ensuring reliable supply chains, adopting shorter and more tolerable regimens, enhancing digital tracking systems, and fostering multisectoral collaboration. Overall, the study highlights that successful TB elimination will require not only technical improvements but also robust health system strengthening and sustained community engagement.  This reviewer commends the authors for their substantial effort and the relevance of their findings on tuberculosis preventive treatment (TPT) implementation. However, several important limitations should be addressed.  - The introduction, methodology, and discussion sections could be streamlined to improve clarity/flow and focus, as the discussion at times extends beyond the scope of the study findings. - The sample size, while including multiple stakeholder groups, is limited to a single district, which restricts the ability to generalize results to broader populations or different programmatic settings. - Without replication across multiple regions, caution is warranted in extrapolating these findings and proposed solutions to national-level implementation. - Furthermore, while the study identifies important barriers and suggests reasonable solutions, many of these are largely intuitive and lack sufficient granularity to directly inform programmatic change. - The manuscript would be strengthened by more detailed, actionable recommendations or implementation frameworks. - Finally, the impact of the work would be significantly enhanced if the proposed interventions were tested through a pilot implementation study demonstrating measurable improvements in TPT uptake and outcomes, thereby moving from descriptive findings to intervention-based evidence. - As pointed out by the authors, Tb remains a problem in specific parts of India, and the applicability of this study for a broader audience of this journal, in this reviewer’s view, remains limited. | The introduction section is modified for clarity  Limitation is mentioned now.  That is very good idea. But it was beyond the scope of this study. It is possible with the government only.  Mentioned in limitations. |
