# Supplementary material for: Factors influencing tuberculosis preventive treatment implementation in India: A hybrid inductive-abductive thematic analysis using implementation framework
Source: PLoS One. 2026 Oct 14;21(10):e0359171. doi: 10.1371/journal.pone.0359171 (PMC13667825; doi:10.1371/journal.pone.0359171)
Supplement: Supplementary file 2 [file pone.0359171.s003.docx]

| Reviewer 2 | Thanks for critically reviewing and giving comments. |
| --- | --- |
| 1. Contradictory reporting of supervisory presence during FGDs: The Methods state that the focus group discussions were conducted in the absence of supervisory officers, whereas the limitations indicate that Taluka Health Officers were present during the FGDs and may have influenced the medical officers’ responses. These statements are mutually inconsistent. The authors should clarify what actually occurred and revise both sections accordingly. This is important because the presence of supervisors could introduce social-desirability bias and affect the credibility and interpretation of the findings. 2. Inconsistencies between the reported numerical summaries and Tables 1–2: As an initial verification step, I used AI-assisted calculations to recompute the weighted means from the participant numbers and subgroup means reported in Tables 1 and 2. The recalculated values do not fully reproduce the figures presented in the manuscript. For example, the reported mean age, overall experience and TB-program experience of the 79 medical officers are 38.35, 10.36 and 6.19 years, respectively, whereas the corresponding weighted means derived from Table 1 are approximately 37.22, 9.38 and 6.05 years. Similarly, the reported mean age and schooling duration of adult contacts are 38.37 and 5.81 years, whereas the weighted means derived from Table 2 are approximately 38.47 and 6.23 years. Although minor differences may result from rounding or missing observations, the manuscript does not provide variable-specific denominators or missing-data information, and the discrepancy in schooling is notable. The authors should independently verify these calculations against the original dataset, recalculate all descriptive statistics, report any missing-data denominators and ensure consistency across the abstract, main text and tables.  3. Incorrect numerical wording and broader reference-quality concerns: The Introduction states that scaling up tuberculosis preventive treatment may “reduce the incidence of TB from about 2.5% to 10%.” A change from 2.5% to 10% represents an increase rather than a reduction. The cited source may instead refer to a reduction by 2.5%–10%, or to a reduction from one specified incidence rate to a lower rate. The original source should therefore be checked and the statement corrected according to the published evidence rather than by simply changing a preposition. More generally, the reference list requires a systematic audit because several entries appear incomplete, truncated or incorrectly formatted, including an incomplete DOI in Reference 2, a truncated title in Reference 4, an apparent ISBN presented as a DOI in Reference 5 and incomplete publication details in Reference 31. All references should be verified against the original publications, with accurate titles, authors, journal details, years, volume and page information, URLs and DOIs. | The statement of absence of supervisory officers meant district level officers like DHO, Addl. DHO, DTO etc. Taluka Health Officer and Medical Officer are from the same cadre and are mutually transferrable posts. Many times they work as MO and are given additional charge of THO. Taluka Health Officer’s main role is coordination. Most Taluka Health Officers are BAMS and promoted from Group B to Group A. Whereas, medical officer from first appointment are in Group A services. We included THOs in FGDs. However, the theoretical possibility of influence is mentioned in the limitations.  The absence of supervisory officer was certainly followed in FGDs of MOs in corporation area.  2. We thank the reviewer for pointing slight variations in statistics. The difference were due to two reasons. First due converting months into years, secondly in Tb experience there was problem in data cleaning. For Doctors working in PHC the total experience and experience in Tb was considered same and written as “do’ or “ . It is rectified now. Now the data has been very carefully analyzed and rewritten.  The sentence is rewritten clarify.  The references are generated by Mendeley. All four references are corrected now. |
