# Supplementary material for: Factors influencing tuberculosis preventive treatment implementation in India: A hybrid inductive-abductive thematic analysis using implementation framework
Source: PLoS One. 2026 Oct 14;21(10):e0359171. doi: 10.1371/journal.pone.0359171 (PMC13667825; doi:10.1371/journal.pone.0359171)
Supplement: Supplementary file 3 [file pone.0359171.s004.docx]

| First round | |
| --- | --- |
| Reviewer 1 | The authors thank the reviewers of constructive comments. |
| 1. Is this a basic qualitative study? Please clarify this point.  2. What are the exclusion criteria?    3. How do you reach the data saturation point? Explain briefly.  4. Do not mention any results in the Method section; instead state them clearly within the result section.  5. Write the name of the software used to process and analyze the data within the method section. 6. In the discussion section, write about the recruitment, such as why some people refuse to participate? 7. Write briefly about the potential role of telehealth in improving tuberculosis preventive treatment approach. 8. Write about the limitations of the study and any potential bias. 9. Check the written abbreviations throughout the manuscript. | 1. Yes, it is a basic qualitative study. It is clearly mentioned now. 2. All those who were available and willing were included. There were no exclusion criteria. 3. In the seventh FGD a few points emerged while in the eighth FGD no additional point emerged. Similarly, in sixth IDI very few points emerged and in the seventh no new point emerged. It is mentioned now. 4. No results/analysis is included in method section 5. We used MAXQDA software. It is already mentioned. 6. As suggested, one sentence is added in discussion. 7. A few lines are mentioned. 8. One important limitation and potential bias is mentioned now. 9. The abbreviations are standard in the Indian context. However, as per the suggestion and keeping international readers full forms are given at first appearance of abbreviations. |
| Reviewer 2 | The authors thank reviewer for critically reviewing and giving suggestions for improving quality of the manuscript. |
| I appreciate the authors for addressing an important public health issue and for employing qualitative methods such as FGDs and IDIs, with the identification of five themes being commendable. However, the manuscript requires substantial revision to improve clarity, structure, and alignment with qualitative research reporting standards. The title needs modification. While knowledge and perceptions can be directly inferred, barriers are largely subjective and context-dependent; therefore, the title should be revised to better reflect this distinction. Additionally, the key terms used in the title and objectives: knowledge, perceptions, barriers, and practices, should be clear and conceptually resolved in the introduction. The introductory section is overly detailed. The initial paragraphs describing TB incidence can be shortened and summarized, and the details of India’s elimination programs can be consolidated into a single paragraph. The study aims also need to be more clearly and precisely stated. There is noticeable repetition between the data collection and data analysis sections, which should be streamlined. The manuscript would benefit from being rewritten in accordance with established qualitative reporting guidelines (e.g., COREQ or SRQR), with clearer description of analytical methods. Although the identification of themes is appropriate, detailing every response from interviews is not necessary. The results section should be more concise, using representative quotations rather than extensive verbatim reporting, and should emphasize synthesis over description. Overall, I kindly advise the authors to thoroughly reframe and rewrite the manuscript after reviewing similar high-quality qualitative publications. As a useful reference, the following article may help guide structure and presentation: <https://jamanetwork.com/journals/jamadermatology/fullarticle/2841453> | The title is modified.  A few sentences are added in the introduction section.  The introduction section is reduced as per the suggestion.  The aim is rewritten.  The repetition is removed.  We have used COREQ guidelines.  We have used the suggested article for data analysis and findings. |
| Reviewer 3 | Many many thanks for reviewing and nicely summarizing the contents of the manuscript. |
| This qualitative study conducted in Pune district, India, explored knowledge, attitudes, and implementation barriers related to tuberculosis preventive treatment (TPT) among medical officers, senior health officials, and adult household contacts of pulmonary TB patients. Using focus group discussions and in-depth interviews, the study identified major gaps in awareness and training regarding TPT, particularly among healthcare providers and community members, along with low uptake and inconsistent implementation of preventive therapy.  Key identified barriers included stigma, migration, limited diagnostic and drug availability, weak monitoring systems, and inadequate engagement of private practitioners, while participants generally expressed positive attitudes toward TB prevention despite skepticism about achieving elimination targets.  Suggested solutions included strengthening community awareness and counselling, improving healthcare worker training, ensuring reliable supply chains, adopting shorter and more tolerable regimens, enhancing digital tracking systems, and fostering multisectoral collaboration. Overall, the study highlights that successful TB elimination will require not only technical improvements but also robust health system strengthening and sustained community engagement.  This reviewer commends the authors for their substantial effort and the relevance of their findings on tuberculosis preventive treatment (TPT) implementation. However, several important limitations should be addressed.  - The introduction, methodology, and discussion sections could be streamlined to improve clarity/flow and focus, as the discussion at times extends beyond the scope of the study findings. - The sample size, while including multiple stakeholder groups, is limited to a single district, which restricts the ability to generalize results to broader populations or different programmatic settings. - Without replication across multiple regions, caution is warranted in extrapolating these findings and proposed solutions to national-level implementation. - Furthermore, while the study identifies important barriers and suggests reasonable solutions, many of these are largely intuitive and lack sufficient granularity to directly inform programmatic change. - The manuscript would be strengthened by more detailed, actionable recommendations or implementation frameworks. - Finally, the impact of the work would be significantly enhanced if the proposed interventions were tested through a pilot implementation study demonstrating measurable improvements in TPT uptake and outcomes, thereby moving from descriptive findings to intervention-based evidence. - As pointed out by the authors, Tb remains a problem in specific parts of India, and the applicability of this study for a broader audience of this journal, in this reviewer’s view, remains limited. | The introduction section is modified for clarity  Limitation is mentioned now.  That is very good idea. But it was beyond the scope of this study. It is possible with the government only.  Mentioned in limitations. |

| Editors comments |  |
| --- | --- |
| 1.Please ensure that your manuscript meets PLOS ONE's style requirements, including those for file naming. The PLOS ONE style templates can be found at  <https://journals.plos.org/plosone/s/file?id=wjVg/PLOSOne_formatting_sample_main_body.pdf> and  [https://journals.plos.org/plosone/s/file?id=ba62/PLOSOne_formatting_sample_title_authors_affiliations.pdf](https://track.editorialmanager.com/CL0/https:%2F%2Fjournals.plos.org%2Fplosone%2Fs%2Ffile%3Fid=ba62%2FPLOSOne_formatting_sample_title_authors_affiliations.pdf/1/010f019dadef921e-35d6462e-de56-452f-880a-a4c7ac006a95-000000/7oL01cYgz0UAVMsJGh1GsNNk6J89ePhgCsSbtF-h0IM=258)  2. During your revisions, please confirm whether the wording in the title is correct and update it in the manuscript file and online submission information if needed. Specifically, remove "Date: 11 Feb. 26 To The Editor" from the title .  3. In the online submission form you indicate that your data is not available for proprietary reasons and have provided a contact point for accessing this data. Please note that your current contact point is a co-author on this manuscript. According to our Data Policy, the contact point must not be an author on the manuscript and must be an institutional contact, ideally not an individual. Please revise your data statement to a non-author institutional point of contact, such as a data access or ethics committee, and send this to us via return email. Please also include contact information for the third party organization, and please include the full citation of where the data can be found.  4. Please amend either the title on the online submission form (via Edit Submission) or the title in the manuscript so that they are identical.  5.We note that Figure 1 in your submission contain map images which may be copyrighted. All PLOS content is published under the Creative Commons Attribution License (CC BY 4.0), which means that the manuscript, images, and Supporting Information files will be freely available online, and any third party is permitted to access, download, copy, distribute, and use these materials in any way, even commercially, with proper attribution. For these reasons, we cannot publish previously copyrighted maps or satellite images created using proprietary data, such as Google software (Google Maps, Street View, and Earth). For more information, see our copyright guidelines: <http://journals.plos.org/plosone/s/licenses-and-copyright>.   We require you to either (1) present written permission from the copyright holder to publish these figures specifically under the CC BY 4.0 license, or (2) remove the figures from your submission:   1. You may seek permission from the original copyright holder of Figure 1 to publish the content specifically under the CC BY 4.0 license.   We recommend that you contact the original copyright holder with the Content Permission Form (<http://journals.plos.org/plosone/s/file?id=7c09/content-permission-form.pdf>) and the following text:  “I request permission for the open-access journal PLOS ONE to publish XXX under the Creative Commons Attribution License (CCAL) CC BY 4.0 (<http://creativecommons.org/licenses/by/4.0/>). Please be aware that this license allows unrestricted use and distribution, even commercially, by third parties. Please reply and provide explicit written permission to publish XXX under a CC BY license and complete the attached form.”   Please upload the completed Content Permission Form or other proof of granted permissions as an "Other" file with your submission.   In the figure caption of the copyrighted figure, please include the following text: “Reprinted from [ref] under a CC BY license, with permission from [name of publisher], original copyright [original copyright year].”   2. If you are unable to obtain permission from the original copyright holder to publish these figures under the CC BY 4.0 license or if the copyright holder’s requirements are incompatible with the CC BY 4.0 license, please either i) remove the figure or ii) supply a replacement figure that complies with the CC BY 4.0 license. Please check copyright information on all replacement figures and update the figure caption with source information. If applicable, please specify in the figure caption text when a figure is similar but not identical to the original image and is therefore for illustrative purposes only.  The following resources for replacing copyrighted map figures may be helpful:   USGS National Map Viewer (public domain): <http://viewer.nationalmap.gov/viewer/>  The Gateway to Astronaut Photography of Earth (public domain): <http://eol.jsc.nasa.gov/sseop/clickmap/>  Maps at the CIA (public domain): <https://www.cia.gov/library/publications/the-world-factbook/index.html> and <https://www.cia.gov/library/publications/cia-maps-publications/index.html>  NASA Earth Observatory (public domain): <http://earthobservatory.nasa.gov/>  Landsat: <http://landsat.visibleearth.nasa.gov/>  USGS EROS (Earth Resources Observatory and Science (EROS) Center) (public domain): [http://eros.usgs.gov/#](http://eros.usgs.gov/)  Natural Earth (public domain): [http://www.naturalearthdata.com/](http://track.editorialmanager.com/CL0/http:%2F%2Fwww.naturalearthdata.com%2F/1/010f019dadef921e-35d6462e-de56-452f-880a-a4c7ac006a95-000000/0JO1OFQBucCzYH-pF4on3e7tBCraFBIlAfRNXMtq3ss=258)  6. Please remove your figures from within your manuscript file, leaving only the individual TIFF/EPS image files, uploaded separately. These will be automatically included in the reviewers’ PDF.  7.If the reviewer comments include a recommendation to cite specific previously published works, please review and evaluate these publications to determine whether they are relevant and should be cited. There is no requirement to cite these works unless the editor has indicated otherwise.   Additional Editor Comments:  The study addresses a relevant implementation gap of TPT uptake in India, however needs to address gaps in methodology and scientific rigor of the study and presented manuscript. Please address review commens raised by the peer-reviewers, alongwith following observations:-   - The study lacks a guiding conceptual or implementation framework, author must anchor the analysis using an established framework (e.g., CFIR or health systems framework) and map findings accordingly. - The manuscript claims a grounded theory approach but does not generate theory. Either develop a clear conceptual model emerging from data or revise the methodological claim. - Findings are primarily descriptive and reiterate known barriers. Deepen analysis by identifying mechanisms, interactions, and causal pathways between themes. - Themes are presented in isolation without integration. Develop cross-theme linkages and present an explanatory model. - There is no prioritization of barriers. Rank or differentiate barriers based on influence or recurrence. - Sampling strategy appears convenience-based and insufficiently justified. Clarify sampling logic and justify participant selection and adequacy. - Data saturation is asserted without methodological explanation. Describe how saturation was assessed and achieved. - Data analysis lacks transparency. Provide detailed coding procedures, including steps from initial coding to theme development. - There is no mention of inter-coder reliability or validation. Include steps taken to ensure analytical rigor (e.g., multiple coders, consensus processes). - Reflexivity is superficially addressed. Expand on how researcher positionality influenced data collection and interpretation. - Results rely heavily on quotes with limited interpretation. Add analytical commentary after each theme to interpret meaning and implications. - Stakeholder perspectives are not sufficiently compared. Author must explicitly contrast findings across medical officers, senior officials, and contacts. - No negative or divergent cases are presented. Include contradictory or minority views noted during the study to strengthen credibility. - The discussion largely repeats results and literature. Reframe the discussion to interpret findings and explain underlying mechanisms. - The manuscript does not clearly articulate its novel contribution. Explicitly state what new knowledge this study adds. - Limited linkage to global or implementation science literature. Strengthen positioning within broader evidence and frameworks. - Conclusions are generic and programmatic. Provide prioritized, actionable recommendations linked directly to findings. - Recommendations lack specificity. Translate findings into concrete implementation strategies or models. - The current statement (“data available on request”) does not meet PLOS requirements. Deposit data in a public repository with open access where feasible. - No justification is provided for restricted data sharing. Clearly state ethical constraints if full transcripts cannot be shared. - No supporting materials are shared. Upload de-identified transcripts or excerpts, a detailed codebook, and an expanded thematic framework. - The manuscript does not provide repository details. Include repository name, DOI/link, and access conditions in the data availability statement. - Methods section contains duplication and inconsistencies. Edit for clarity and remove redundant text. - The manuscript does not fully align with COREQ reporting standards. Revise to ensure complete qualitative reporting. - Language and grammar issues reduce readability → Undertake professional language editing. - The abstract is overly descriptive and lacks analytical insight. Revise to highlight key findings and their implications. - The title is lengthy and unfocused. Thus, shorten and sharpen to reflect the core contribution. - Figures (e.g., thematic diagram) are descriptive rather than analytical. Redesign figures to show relationships and explanatory pathways. | The style has been modified as suggested  It is confirmed and there is no difference. The date is removed.  All the data is available with Institutional Ethics Committee and available on demand.  The titles are now identical.  The figure is not copy righted. The map is obtained from website of **M**aharashtra **R**emote **S**ensing **A**pplication **C**entre (MRSAC), which is set up by Government of Maharashtra. The map is then modified suitably for the manuscript. For accurate map, one is expected to use authentic source only. The map is available from: <https://mrsac.gov.in/writereaddata/MRSAC/map/15672515405d6a5c544ada0TH_PCT_Baramati.pdf>  The figures are removed from word document.  One reviewer has suggested one article and we found that it is useful for writing style.  We agree that the study did not use CFIR framework. After the suggestion we have used CFIR for analysis.  We used a hybrid inductive-abductive thematic analysis and is mentioned.  After use of CFIR the Figure 2 is suitably changed.  In the analysis some linkage are developed.  The barriers have been prioritized.  Sampling information is mentioned.  The contextual meaning of saturation is mentioned now.  The coding procedure is mentioned now.  It is mentioned in the limitations.  All five points about the reflexibility are written as expected in COREQ. The presence of Taluka Health Officer may have influenced medical officers’ response. It is mentioned now.  The quotes are reduced and some interpretation is written now.  Few sentences are added.  The data analysis section is modified.  The study combines the providers and beneficiaries perspectives and it is mentioned now.  The study did not intend to compare other countries which have different social structure. However, as suggested few references are added from developing countries.  Now specific recommendations are made.  The information availability is modified.  It is not available on any website. But as suggested the data is submitted to the Institutional Ethics Committee. Supplementary files are attached and uploaded.  Supplementary files are attached and uploaded.  Supplementary files are attached and uploaded.  Duplication is removed.  Maximum guidelines of COREQ are followed.  Many sections are rewritten.  Abstract rewritten.  The title is shortened and changed.  Figure 2 is changed. |

| Reviewer 2 | Thanks for critically reviewing and giving comments. |
| --- | --- |
| 1. Contradictory reporting of supervisory presence during FGDs: The Methods state that the focus group discussions were conducted in the absence of supervisory officers, whereas the limitations indicate that Taluka Health Officers were present during the FGDs and may have influenced the medical officers’ responses. These statements are mutually inconsistent. The authors should clarify what actually occurred and revise both sections accordingly. This is important because the presence of supervisors could introduce social-desirability bias and affect the credibility and interpretation of the findings. 2. Inconsistencies between the reported numerical summaries and Tables 1–2: As an initial verification step, I used AI-assisted calculations to recompute the weighted means from the participant numbers and subgroup means reported in Tables 1 and 2. The recalculated values do not fully reproduce the figures presented in the manuscript. For example, the reported mean age, overall experience and TB-program experience of the 79 medical officers are 38.35, 10.36 and 6.19 years, respectively, whereas the corresponding weighted means derived from Table 1 are approximately 37.22, 9.38 and 6.05 years. Similarly, the reported mean age and schooling duration of adult contacts are 38.37 and 5.81 years, whereas the weighted means derived from Table 2 are approximately 38.47 and 6.23 years. Although minor differences may result from rounding or missing observations, the manuscript does not provide variable-specific denominators or missing-data information, and the discrepancy in schooling is notable. The authors should independently verify these calculations against the original dataset, recalculate all descriptive statistics, report any missing-data denominators and ensure consistency across the abstract, main text and tables.  3. Incorrect numerical wording and broader reference-quality concerns: The Introduction states that scaling up tuberculosis preventive treatment may “reduce the incidence of TB from about 2.5% to 10%.” A change from 2.5% to 10% represents an increase rather than a reduction. The cited source may instead refer to a reduction by 2.5%–10%, or to a reduction from one specified incidence rate to a lower rate. The original source should therefore be checked and the statement corrected according to the published evidence rather than by simply changing a preposition. More generally, the reference list requires a systematic audit because several entries appear incomplete, truncated or incorrectly formatted, including an incomplete DOI in Reference 2, a truncated title in Reference 4, an apparent ISBN presented as a DOI in Reference 5 and incomplete publication details in Reference 31. All references should be verified against the original publications, with accurate titles, authors, journal details, years, volume and page information, URLs and DOIs. | The statement of absence of supervisory officers meant district level officers like DHO, Addl. DHO, DTO etc. Taluka Health Officer and Medical Officer are from the same cadre and are mutually transferrable posts. Many times they work as MO and are given additional charge of THO. Taluka Health Officer’s main role is coordination. Most Taluka Health Officers are BAMS and promoted from Group B to Group A. Whereas, medical officer from first appointment are in Group A services. We included THOs in FGDs. However, the theoretical possibility of influence is mentioned in the limitations.  The absence of supervisory officer was certainly followed in FGDs of MOs in corporation area.  2. We thank the reviewer for pointing slight variations in statistics. The difference were due to two reasons. First due converting months into years, secondly in Tb experience there was problem in data cleaning. For Doctors working in PHC the total experience and experience in Tb was considered same and written as “do’ or “ . It is rectified now. Now the data has been very carefully analyzed and rewritten.  The sentence is rewritten clarify.  The references are generated by Mendeley. All four references are corrected now. |

| PlosOne: Editor’s comments: The authors than the editor for constructive suggestions. We have added two paragraphs in response to the comments. | |
| --- | --- |
| (A) Strengthen the analytical interpretation by developing explicit relationships and explanatory pathways between themes rather than presenting largely descriptive findings. | One paragraph before discussion section and one at the end of discussion is added. |
| (B) Provide a clear comparative analysis across the three stakeholder groups, highlighting convergent and divergent perspectives instead of combining them into a single narrative. | One figure is added to display the common and discrete themes/codes emerged from the discussions and interviews. |

| **Focused corrections** | |
| --- | --- |
| **Corrections** | Author’s response |
| **FGD supervision:** Clarify in the Methods and Limitations that district-level supervisory officers were absent, while THOs (of same cadre) participated in some FGDs. | In Method section and strength and limitations necessary suggested changes are incorporated. |
| **Education denominator Clarity:** Correct the sentence with data: misspelled currently as " Only and 29..." May correct with *“**Only 29 of the 43 adult contacts responded to the question on duration of schooling. The mean duration among these 29 respondents was 8.97 (SD 4.76) years.”* Please indicate the denominator in Table 2 if possible. | Thanks for proving correct sentence. The original sentence is deleted.  Denominator included in Table 2. |
| **TPT/incidence statement:** Revise the sentence referring to a “2.5 to 10% reduction” so that it accurately explains what these figures represent and is supported by the appropriate reference. |  |
| **Reference 10:** Please verify and correct the citation/DOI. The DOI currently listed corresponds to a different article, not the cited Basu article. Please ensure the reference cited is the actual source supporting the statement. | I apologize for the mistake. It is corrected now. |
