# Supplementary material for: Factors influencing tuberculosis preventive treatment implementation in India: A hybrid inductive-abductive thematic analysis using implementation framework
Source: PLoS One. 2026 Oct 14;21(10):e0359171. doi: 10.1371/journal.pone.0359171 (PMC13667825; doi:10.1371/journal.pone.0359171)
Supplement: Supplementary file 4 [file pone.0359171.s001.zip › In-depth Interview.zip!/In-depth Interview/IDI_1.pdf]

In-depth Interview: 1

Date: 05/12/2023
(Audio recording consent was requested, but he denied permission for audio recording.)

I: How many yearly TB cases are registered?
R: Approximately 8,000 TB cases are registered annually.

I: What proportions are children, pregnant women, adults, and the elderly?
R: The proportion of children is about 10%. The elderly population (aged 60+) accounts for approximately 25% of cases. The remaining cases are mainly in the productive age group of 18–50 years. The proportion among pregnant women is comparatively low.

I: Change in the number of cases in the last five years. Change in the number of cases pre- and post-COVID. Change in the proportion of children.
R: Previously, the proportion of cases reported from public facilities was higher. Cases from both pre- and post-COVID periods were considered through the Nikshay portal. The number of cases may vary, but the program is target-oriented, aiming for not less than 80% achievement.

I: Have you heard about TPT?
R: No. I worked in the TB program as a DTO until 2017. I am now working at the corporation, and no TPT training has been conducted yet.

R: Regarding relevance, INH prophylaxis is important. If feasibility targets are maintained, it is achievable for children. If the guidelines are improved and properly implemented, then the targets can be achieved.

I: Please tell us about the extent of coverage of these guidelines followed under your health facility. What is the rough proportion of manpower that has undergone training? What about private practitioner training? Are there differences across setups, such as medical colleges, private-sector hospitals, and public-sector hospitals?
R: I am currently taking follow-up, but I learned about this only today, as no one had informed me earlier. The private sector has been involved.

I: Challenges.
R: There are no major challenges. He may be referring to PMC health officers. Welfare society members conduct awareness marches.

I: How do you overcome these challenges?
R: Some guidelines are available, but I am not fully aware of them, so I cannot explain in detail. The system changed after COVID.

I: What about TB elimination?
R: During 2013–14, I worked in the TB elimination program. At that time, there was a greater focus on private-sector involvement and case detection. Currently, the Nikshay Poshan Yojana is being

implemented, and nutritional support has started. However, despite efforts to maintain adherence and provide good facilities, there are still weak components in the system.

In slum areas, workers are often not allowed entry, and in affluent societies, municipal corporation workers (MANAPA staff) are not easily accepted. Therefore, adherence cannot always be properly assessed. In slums, treatment is often taken from private providers or others, so supportive supervision is required. In rural areas, ASHA workers are available, making management easier. Urban areas are more difficult to handle.

The focus should be on treatment adherence. Funds are not the issue; supervision, treatment adherence, and technical ignorance are bigger concerns. I do not think TB elimination by 2025 is possible. There is still around a 10% gap in diagnosis. Ideally, 100% diagnosis should occur, but that is not happening. Nutrition remains inadequate, despite CSR support.

Among 500 contacts and 500 patients screened, around 60–70% coverage was achieved. Proper screening, diagnosis, and treatment can make elimination easier. CSR involvement can be made mandatory at the state level, especially for nutritional support. A timely supply of drugs is also necessary. If PMC support is required, we provide it. The TB cell provides funds, and, if required, PMC also provides financial support.

I: What is your opinion about TPT and other components contributing to TB elimination by 2025?
R: In my personal opinion, TB elimination by 2025 is not possible.

I: In your opinion, what is the possibility of TB elimination by 2025?
R: I will learn more about it and inform my seniors.
