# Supplementary material for: Factors influencing tuberculosis preventive treatment implementation in India: A hybrid inductive-abductive thematic analysis using implementation framework
Source: PLoS One. 2026 Oct 14;21(10):e0359171. doi: 10.1371/journal.pone.0359171 (PMC13667825; doi:10.1371/journal.pone.0359171)
Supplement: Supplementary file 4 [file pone.0359171.s001.zip › In-depth Interview.zip!/In-depth Interview/IDI_2.pdf]

In-depth Interview: 2

Date: 09/11/2023

I: What is the approximate population under your supervision?

R: The rural population under my supervision is around 38 lakhs.

I: Approximately how many yearly TB cases are registered?

R: Around 9,000 cases per year.

I: Approximately how many children are registered among these cases?

R: The target is around 9,000 cases per year.

I: Meaning that many cases are actually registered?

R: No, that is the target. The performance is around 94%, so approximately 8,500 cases are registered.

I: What is the proportion of children among these registrations?

R: Around 5%.

I: Has there been any change in registrations over the last five years, especially before and after COVID?

R: I do not know.

I: When did you first get to know about the guidelines for TB preventive therapy?

R: We knew about prophylaxis, but not specifically about the treatment period.

I: Did you receive any training regarding it?

R: No.

I: Would you be able to mention the key or important points about prophylaxis?

R: We provide prophylaxis with INH to some close contacts, especially children living in the same household.

I: Okay. How long is the treatment duration?

R: Six months.

I: Which drug is given?

R: INH.

I: What is the dosage?

R: I do not know.

I: The Government of India has decided on a target to eliminate TB by 2025. What is your opinion about this?

R: TB is basically a chronic disease, but now effective treatment regimens are available and accepted. Some villages are already approaching elimination status. Therefore, we may achieve the elimination target by March 2025.

I: So you feel it is achievable, or are there some challenges?

R: If complete treatment is properly taken and the guidelines given by the National TB Division are followed correctly, then there should not be a problem in achieving elimination.

I: Okay. This is regarding diagnostics. What about other aspects?

R: Other support, such as nutritional and financial support, is also provided to patients. Therefore, there are no major difficulties in treatment completion.

I: Can you comment on the coverage of these guidelines in Pune Rural?

R: Which guidelines, Madam?

I: The TB preventive therapy guidelines. Would you be able to provide an idea about their coverage?

R: No.

I: What about manpower training regarding these guidelines or training for private practitioners?

R: That has not been done, Madam.

I: Meaning it has not been done, or you do not know about it?

R: I do not know.

I: Are private practitioners involved in the training, and what about their role?

R: Private practitioners are involved in every TB-related activity.

I: What about corporate hospitals?

R: They are also involved because around 10–15% of cases are managed in the private sector.

I: Okay.

R: So, there is involvement of private practitioners in every TB activity.

I: What about the involvement of medical colleges in Pune, especially Pune Rural?

R: Yes, medical colleges are involved.

I: Are there any challenges in implementing these preventive therapy guidelines? If yes, could you explain?

R: Currently, no major challenges are known.

I: So, there are no challenges in implementation?

R: Not known.

I: You mentioned that the TB elimination program is feasible. On what basis do you say it is feasible? Please share your suggestions, opinions, or experiences.

R: The treatment completion rate is more than 95%, resistant cases are fewer, and patient compliance is good.

I: Why do you think resistance is less?

R: Early diagnosis of resistance can be done, so if any change in regimen is required, it can be initiated in the early stages.

I: And regarding diagnostic availability?

R: Early diagnosis can now be done due to the CBNAAT machine.

I: Okay. Is elimination easier because of preventive therapy?

R: Preventive therapy provides support, so elimination is possible.

I: Any other comments? Thank you for your valuable time and responses. They are valuable from our research perspective.

Note: The DHO had another scheduled meeting, so the interview was conducted within a short period of time.
