# Supplementary material for: Factors influencing tuberculosis preventive treatment implementation in India: A hybrid inductive-abductive thematic analysis using implementation framework
Source: PLoS One. 2026 Oct 14;21(10):e0359171. doi: 10.1371/journal.pone.0359171 (PMC13667825; doi:10.1371/journal.pone.0359171)
Supplement: Supplementary file 4 [file pone.0359171.s001.zip › In-depth Interview.zip!/In-depth Interview/IDI_3.pdf]

In-depth Interview: 3

Date: 20/10/2023

I: Your designation will be required. This is the consent form in front of you, which states that you are willing to participate.

R: Okay.

I: Our intention is not different from the government line. We only want to understand the current situation and challenges. This is not about speaking against the government.

R: Okay.

I: What is the approximate population under your supervision?

R: Around 35 lakh.

I: PMC?

R: Yes, PMC.

I: How many yearly TB cases are registered?

R: Around 7,500 cases from both public and private sectors combined.

I: What is the proportion of children among these 7,500 cases, especially pre- and post-COVID?

R: Around 10–15%.

I: Was there any change in that?

R: Yes. During COVID, patient registration reduced because patients were not diagnosed. The graph showed a rising trend before and after COVID.

I: Okay. Was it less during post-COVID?

R: During COVID, especially during lockdown, the numbers were lower.

I: Then it took almost two years to return to the same level as pre-COVID?

R: Yes.

I: When did you first hear about TB preventive treatment guidelines?

R: Approximately 10 years ago, when I was working as a Medical Officer.

I: Okay. Around 2012–13?

R: Yes. TPT guidelines were available at that time. INH chemoprophylaxis was also being used.

I: Now the format of TPT has changed. Did you receive any training regarding it?

R: Yes, we received training.

I: When?

R: One year ago.

I: Was the training conducted for all categories of staff?

R: Yes.

I: Where was the training conducted?

R: At Aundh.

I: Discussions about TPT have been happening for the last two years, but due to COVID, the proper implementation of guidelines was delayed.

R: Yes. The guidelines were ready, but training started late.

I: Please explain, priority-wise, the key or important aspects in the TPT guidelines.

R: Our first priority is contacts of pulmonary TB patients because, according to TPT guidelines, open pulmonary cases are the highest priority. After that, we focus on pediatric age groups, then on other age groups.

I: Is there a separate priority for the pediatric age group?

R: We mainly miss only those children who have weight loss or are already taking multiple medicines. Otherwise, we try to cover almost 100% in the public sector.

I: Okay. Open pulmonary cases are the first priority, and the public system is the second priority.

R: Private sector patients are also included. One NGO, under the Akshaya Plus Project, is working with us for TPT. Including both public and private sectors, our TPT coverage is around 73%. More than 80% coverage has been achieved in the public sector.

I: Okay.

R: Cases that are transferred out to other districts are often missed.

I: Meaning, if 10 cases are transferred out, it becomes difficult to trace their close contacts for treatment?

R: Yes. Once they move to another district, our control ends, and the center in that district must take action.

I: What is the approximate proportion of migration?

R: Around 15%.

I: Earlier, you mentioned that some people resist treatment. Approximately how much is that?

R: Around 5–10%.

I: Are TPT guidelines relevant, especially regarding contact treatment?

R: Yes, they are relevant. However, instead of focusing only on single-drug therapy, there should be more focus on 3HP therapy. It is feasible, but transfer-out cases reduce feasibility.

I: Are there any other challenges related to TB?

R: Yes. Suppose there are 15 patients. If contact tracing and counseling are done immediately after diagnosis, then contacts usually agree to treatment. But if contact tracing is delayed by even one month, the patient starts feeling better, and relatives feel treatment is unnecessary. Prompt action is the key.

I: Regarding targets?

R: This does not happen for all patients. It may be possible in approximately 80% of cases. About 10% refuse treatment, and another 10% from the private sector do not respond. If there are seven patients, it may only be possible to trace four contacts. After conducting IGRA on paper, a proper investigation is still required.

I: What are the challenges at the ground level, and how can they be overcome?

R: Yes, there are challenges. For example, IGRA testing cannot be done in every district because maintaining the cold chain is not cost-effective for Maharashtra. These are major issues. Therefore, some districts follow a "treat-only" model.

I: If we follow a treat-only model, will transmission reduce?

R: Certain districts in Maharashtra were randomly selected for the "test-and-treat" model, while 60–70 districts follow the "treat-only" model. We mainly focus on positive pulmonary open cases and their contacts, and results from the test-and-treat model will help determine future cut-offs.

I: What about TPT training?

R: All NTEP staff profiles, such as STS, MOTB, and Medical Officers working under PMC have undergone training.

I: Have all undergone training?

R: Yes. We have also conducted CME programs in the private sector, where training was provided.

I: Are there differences between different setups? Do public sector staff attend training conducted under the corporate sector?

R: Yes. If training is conducted uniformly, they definitely attend.

I: Through IMA or other organizations?

R: Under NTEP, an NGO called Union works with us. It arranges training twice a year.

I: What about private practitioners?

R: We arrange 2–3 trainings. One CME usually covers around 70–80% of doctors. These are mainly organized for chest physicians and MD Medicine specialists. TPT training is also provided, but private MBBS doctors have not received recent training. Their trainings are scheduled for this month and next month.

I: Was this specifically a TPT CME?

R: Not entirely focused on TPT.

I: So, TPT training has mainly been conducted for MD physicians, chest physicians, and corporate/private sector doctors?

R: Yes.

I: What about medical colleges?

R: Yes, medical colleges are also included.

I: We discussed challenges regarding the guidelines. Since IGRA is not available everywhere, many districts follow the treat-only approach. What do you think about ADR challenges?

R: ADRs are not very common with INH. Around 5% ADRs may occur. With 3HPR regimens, ADRs occur in about 5–10% of cases, but no more than that. We monitor patients locally for a few days, then decide whether to continue or withhold treatment.

I: Are most ADRs mild?

R: Yes, mostly mild to moderate. Severe cases requiring hospitalization are uncommon.

I: In your opinion, is it possible to follow the guidelines completely?

R: Yes.

I: Suppose implementation expands further in the future?

R: There are many challenges across the country, but in districts where TPT is functioning well, around 60% of contacts have started TPT.

I: So, is it possible to follow the guidelines with proper training and curriculum?

R: Yes. Communication with patients should be good. If patients and their contacts accept the treatment, then implementation is possible.

I: Mainly for prevention, right?

R: Yes. These suggestions are related to TB elimination through TPT. However, even if the target is set for 2025, elimination may not happen by then. It may be more realistic by 2035.

I: So, according to you, the elimination target should be 2035 instead of 2025?

R: Yes. This is a long-term process. Our graph is still showing an increasing trend. After the increase, there will be a plateau phase, followed by a declining trend. Therefore, it may take another 10 years, regardless of the 2025 target.

R: That is why there is a strong focus on TPT, so that contact patients and family members at risk can be identified early. Often, three or four patients from the same family are diagnosed at different stages. Therefore, once a patient is diagnosed, contacts should immediately receive TB preventive therapy, and early diagnosis and treatment should be carried out through contact screening.

I: What about patient compliance?

R: Compliance and adherence are monitored at the field level, but there are many problems. Around 10% of patients have alcohol addiction or similar issues. Because of resistance and

comorbidities, some patients are unwilling to continue treatment. Sometimes patients are found living on roadsides or outside their homes because their families do not allow them inside. Some patients do not have a permanent address or contact number, making them difficult to track. Pregnant patients are also sometimes lost to follow-up because adherence cannot be ensured. Therefore, separate centers for such patients could be helpful.

R: In our state, sanatoriums exist for homeless patients so that they can stay there during treatment. Similar systems already exist in the army for leprosy treatment, in which patients remain until treatment is completed.

I: We cannot keep all patients in sanatoriums, but as you are saying…

R: Many patients do not even get adequate food twice a day. They take tablets on an empty stomach and then suffer from side effects such as acidity and ADRs. Therefore, pre-counseling is essential. Patients should be properly convinced and emotionally supported. All our staff have undergone counseling training. At Aundh Hospital, patients receive comprehensive counseling at the start to improve adherence. However, if a patient leaves treatment midway, it becomes difficult to continue counseling effectively.
