# Supplementary material for: Factors influencing tuberculosis preventive treatment implementation in India: A hybrid inductive-abductive thematic analysis using implementation framework
Source: PLoS One. 2026 Oct 14;21(10):e0359171. doi: 10.1371/journal.pone.0359171 (PMC13667825; doi:10.1371/journal.pone.0359171)
Supplement: Supplementary file 4 [file pone.0359171.s001.zip › In-depth Interview.zip!/In-depth Interview/IDI_4.pdf]

In-depth Interview:4

Date: 17/11/2023

I: I am Dr. Jayashree Gothankar, one of the investigator coordinators. I will ask you some questions from the interview guide that I have. What is the population under your supervision?

R: A population of around 50 lakh is under my supervision. I am looking after TB work in Pune Rural, which covers approximately 50 lakh people.

I: Normally, how many yearly TB cases are registered?

R: Last year, we had 7,415 registered cases. On average, around 7,000 TB and DSTB cases are registered annually in Pune Rural.

I: Among these, how many children are registered?

R: Around 5-6% are children.

I: In the last five years, has the trend remained consistent with the targets, especially regarding pre- and post-COVID TB cases?

R: One noticeable trend over the last 5-6 years is that we are finding more extrapulmonary TB cases.

I: Okay.

R: One reason may be that Pune has a larger number of tertiary care centers. We have six medical colleges along with the TB Chest Hospital. This may be one reason.

I: Meaning more diagnosis?

R: Yes, more diagnosis is taking place. Extrapulmonary TB is being diagnosed more because we have an extended diagnostic algorithm. Therefore, we are identifying more extrapulmonary cases. There is definitely a shift, with pulmonary cases comparatively reducing.

I: Meaning reduced infectivity?

R: Yes. We are finding more extrapulmonary TB cases.

I: Especially regarding the number of cases pre- and post-COVID?

R: There is no major difference overall.

I: Meaning post-COVID detection rates had reduced, and now they are as good as pre-COVID levels, right?

R: During COVID, detection was lower because patients lacked transportation and could not reach healthcare facilities. Therefore, during that period, only around 4,000–5,000 cases were detected.

I: In 2020–2021?

R: Yes, in 2020–21. After that, the number of cases again increased to around 7,000–8,000 as before.

I: Okay. When did you first hear about TB preventive guidelines?

R: It started around October. I think the first training was conducted in October 2020 or 2021. I do not remember the exact year.

I: Meaning it happened just before COVID?

R: Yes, just before COVID, but implementation started after COVID.

I: Before the training, did you know these guidelines would be introduced?

R: Yes. We were already giving TB preventive therapy to children, but we were not aware that it would also be extended to adults.

I: INH is given to children, and IAP wanted Rifampicin to be added.

R: But currently, only INH is included in the program.

I: Have you received any training regarding TPT?

R: Yes, training has been conducted.

I: In October 2020?

R: Yes, in October 2020.

I: Priority-wise, what are the important aspects in the TPT guidelines?

R: Pulmonary TB contacts are included in the key guidelines, whereas extrapulmonary TB contacts are not included. Drug-resistant TB contacts are also not included. Only contacts of drug-sensitive pulmonary TB cases are included.

I: Their contacts?

R: Sorry, their contacts? Yes.

I: So, extrapulmonary contacts are not included, and contacts of drug-resistant TB cases are also not included?

R: Yes, contacts of drug-resistant TB cases are not included.

I: Then what are the guidelines for contacts of drug-resistant TB cases?

R: There are still no finalized national guidelines for drug-resistant TB contacts. It is currently under trial.

I: Okay.

R: They currently only have screening criteria.

I: Would you be able to elaborate more on why extrapulmonary cases are not included and what is done for pulmonary cases?

R: When a pulmonary TB patient is diagnosed, we take a detailed history of the patient. Earlier, we focused mainly on children below 5 years because they were receiving INH prophylaxis. After the new guidelines were introduced, we started enlisting all contacts. After enlisting them, we screen them for TB symptoms. If they have symptoms such as a cough, they undergo further screening and evaluation according to the existing diagnostic algorithm. Once TB is ruled out, we start TPT.

At the start of TPT, we counsel them that it must be taken as preventive medicine for the full six-month course. In Pune, fortunately, we have support from an agency called Akshaya Plus, which is assisting us in this process.

I: Okay.

R: Additionally, from this year onward, it has been added to the PIP that if ASHA workers supervise TPT and ensure completion of all 156 doses, they will receive an incentive of ₹250. This component has now been included in the PIP.

I: Okay. How do you confirm that six months of treatment have been completed? Is there any indicator?

R: We maintain TPT cards after TB treatment is completed. Based on the returned empty blister packs, we assess whether the doses were taken. From this year onward, ASHA workers are directly supervising patients.

I: Will this start from the financial year April 2024?

R: No, it has already started from 2023–24.

I: Is there any relevance, feasibility, or achievability regarding the 2025 NTEP elimination target, especially concerning IGRA tests, Tuberculin testing, and TB treatment?

R: Currently, Pune district is under the “treat-only” model. Therefore, IGRA testing is not being conducted in our district.

I: On what basis was our district selected for the treat-only model?

R: I am unable to say what criteria were used, but our district was included in the treat-only model.

I: What about Tuberculin testing/treatment?

R: Tuberculin testing is only performed at medical colleges. For example, a pediatric OPD runs every Tuesday at B.J. Medical College, where PPD testing for Tuberculin is conducted and treatment protocols are established.

Some private practitioners also conduct testing, but in the general public health system we are currently unable to perform Tuberculin tests routinely.

I: How much is the coverage for these guidelines?

R: As reviewed yesterday by the Collector, Pune district currently has 76% coverage for TPT guidelines.

I: How much manpower training has been completed?

R: Our training coverage is 100%.

I: What about training for private practitioners?

R: Training for private practitioners has also been conducted.

I: Are they cooperating?

R: Yes, they are cooperating.

I: Are these activities conducted through IMA?

R: Yes, mostly through IMA.

I: Among private medical colleges, corporate hospitals, and the public sector, is there any difference in cooperation?

R: Corporate hospitals are mostly located in corporation areas, so our interaction is comparatively less because we mainly work in rural areas. However, there is a project called "Union Supported TB-Free Workplaces." Through this initiative, awareness is spread in industrial areas, with emphasis on preventive aspects. In crowded workplaces, HR departments are generally cautious because they do not want to lose man-hours.

I: Are there any challenges while implementing the guidelines?

R: Initially, there were many challenges. If we consider 10 patients, for example, there may be 6 pulmonary TB patients. While treating those 6 patients, we also need to provide TPT to around 24 contacts. Ensuring consumption of 180 doses of TPT by a healthy individual is quite challenging.

I: Especially when they feel they do not have TB?

R: Yes. Since they do not have TB symptoms, convincing them to take preventive medicine is challenging.

I: So cooperation from contacts, whether family or workplace contacts, becomes difficult?

R: Close family members are usually willing to take tablets because they understand the risk from the infected family member. However, more distant contacts, such as brothers or workplace contacts, are different.

I: Basically, because of social stigma, they do not want to reveal it?

R: Yes. The main challenge is making healthy individuals consume 180 tablets. Therefore, we are shifting toward the 3HP regimen.

I: Meaning?

R: In the 3HP regimen, one tablet is taken once a week for 12 weeks, totaling 12 doses.

I: How can these barriers be overcome?

R: Continuous education is essential. ASHA workers are a major component in overcoming these barriers. We advocated for incentives for ASHA workers in the PIP, and ultimately, we succeeded in implementing them.

I: Do you think it is possible to fully follow all these guidelines across all health facilities under your supervision?

R: Yes, it is possible.

I: Our main goal is to eliminate TB.

R: Yes.

I: Based on your experience and knowledge, what are your suggestions and opinions regarding this?

R: It is possible.

I: Is it feasible?

R: Yes, it is feasible.

I: Which areas should receive more focus?

R: We have started a new initiative called "TB Mukta Panchayat/Abhiyaan." We are beginning elimination efforts from the grassroots level. On World TB Day in 2023, in Varanasi, the Honorable Prime Minister announced that TB Mukta Panchayat initiatives should be launched. This initiative has been very well adopted by every state. Even our Governor is interested in this issue. Therefore, if the movement starts from the grassroots level, we can definitely achieve the target.

I: Everything seems good in rural areas. What about urban areas, especially slums, where there is more chaos?

R: We should not think negatively like that.

I: Okay. Your opinion about TPT and other components contributing to TB elimination?

R: For TB elimination, I would again emphasize the TB Mukta Panchayat approach.

I: Meaning a field-to-central approach?

R: Yes, a field-to-central approach is one strategy.

I: Okay.

R: Secondly, we are also taking social support. Under the "Pradhan Mantri TB Mukta Bharat Abhiyaan," launched on 9 September 2022 by the Honorable President, generous individuals and Nikshay Mitras are encouraged to adopt TB patients and provide nutritional support. In Pune Rural, coverage under this initiative is around 60%.

I would specifically like to mention that our former President, Pratibha Tai Patil Madam, adopted 62 patients in Pune Rural. Maval MLA Sunil Anna Shelke adopted 351 patients. Skoda Company in Khed adopted 900 patients. Therefore, there is a significant contribution from society and the corporate sectors as well.

I: So you feel that TB can be eliminated by 2025?

R: It is possible.

I: Okay. Thank you for your support, cooperation, and valuable time.

R: Thank you.
