# Supplementary material for: Factors influencing tuberculosis preventive treatment implementation in India: A hybrid inductive-abductive thematic analysis using implementation framework
Source: PLoS One. 2026 Oct 14;21(10):e0359171. doi: 10.1371/journal.pone.0359171 (PMC13667825; doi:10.1371/journal.pone.0359171)
Supplement: Supplementary file 4 [file pone.0359171.s001.zip › In-depth Interview.zip!/In-depth Interview/IDI_5.pdf]

In-depth Interview: 5

Date: 18/12/2023

I: What is your opinion regarding the involvement of medical colleges in TB elimination?

R: All medical colleges should be actively involved in admitting and treating all MDR-TB patients, and treatment should be provided free of cost. Active participation by medical colleges is essential for TB elimination, as DM consultants are available there.

I: Which medical colleges are actively involved?

R: B.J. Hospital and D.Y. Patil Medical College are very active in TB-related work. RNTCP laboratories should also be established in medical colleges, and NIT laboratories and diagnostic facilities should be available there as well.

I: What is the approximate population under your supervision?

R: The approximate population under supervision is around 24 lakh people.

I: Approximately how many TB cases are registered annually?

R: Around 3,500 TB cases are registered annually, including both private and government sector cases, as well as pulmonary and extrapulmonary TB cases.

I: How many newly diagnosed cases are there per year?

R: Around 700–800 newly diagnosed cases are reported every year.

I: What proportion of the registered cases are children?

R: Around 6% of the registered cases are children.

I: Have there been any changes in TB cases during the post-COVID period or over the last five years?

R: Complicated cases such as military TB and pneumothorax have reduced in the post-COVID period and over the last five years.

I: When was TB preventive treatment (TPT) implemented?

R: TPT implementation became difficult during the COVID pandemic, but the program has been functioning since around 2022, after COVID.

I: Were there any issues related to drug availability?

R: Yes. Drug availability has been an issue. For example, the Amphotericin drug supply was not properly available for nearly six months.

I: When does TPT start?

R: Whenever a TB patient is newly diagnosed, TPT is initiated for eligible contacts. In adults, TPT is started if the IGRA test is positive.

I: When was TPT training conducted?

R: Training regarding TPT was conducted around one and a half years ago, and 100% training has been completed for all working staff.

I: Were any new guidelines introduced regarding IGRA-positive patients?

R: Yes. Newer guidelines have been introduced for IGRA-positive patients. However, the 3HP regimen has not yet been fully implemented and is currently mainly for adults.

I: Are there any treatment-related obstacles?

R: Yes. People are often scared, and detailed explanation and counselling are required. Private practitioners and the private sector are not fully prepared, and proper explanation regarding TB and TPT is needed for patients. Many people in the private sector are unaware that they have TB.

I: Is there any gap in health facility coverage in PCMC?

R: Some cases may still be missed. Since 2013, around 1,000 private sector cases have remained constant annually, while the target is around 1,100 cases.

I: What is the role of medical colleges in TB detection?

R: Medical colleges contribute significantly to TB detection. Around 30–50% of cases are detected through medical colleges. For example, if there are 250 monthly cases, around 100 cases are detected at D.Y. Patil and YCM Hospital.

I: Are there any gaps in TPT-related training among private practitioners?

R: Yes, there is still a gap in TPT-related training among private practitioners.

I: How many DOTS centers are present in the private sector?

R: There are around 450 DOTS centers in the private sector, and general practitioners directly refer patients to the government system. There is a good tie-up with private practitioners.

I: What are the major challenges related to TPT implementation?

R: The main challenges are:

- Drug availability
- IGRA test availability
- Reluctance of private practitioners regarding preventive drug use

- Need for counseling in the private sector

If patients are properly counseled, they are more likely to accept treatment.

I: How are these difficulties handled?

R: These difficulties are handled through regularly organized conferences and IME programs. Active participation from pulmonologists is necessary, and repeated awareness activities are required.

I: How can issues related to IGRA testing and drug availability be improved?

R: These issues can be improved if the private sector is more closely integrated into the program. If testing facilities are expanded in the government sector, these problems can be reduced. Alternative, more readily available tests are also required.

I: What is your opinion regarding the 2025 TB elimination goal?

R: Greater involvement of the private sector is necessary. Participation from medical colleges should increase further. Vaccination trials should also be successful if possible.

I: What other measures are needed for TB elimination?

R: Socioeconomically disadvantaged populations, especially people living in slums and urbanized areas, require additional support. Awareness about TB should be increased through media campaigns and advertisements. Health education should also be provided to people suffering from TB.

I: What role can recovered TB patients play?

R: Patients who have completed treatment should participate in group discussions and meetings with newly diagnosed patients. They can guide and motivate TB patients who are undergoing treatment. Such meetings are conducted monthly.

I: What are the major drawbacks in TB control?

R: Major drawbacks include delayed detection among migrant workers and construction workers. Detection in these groups is slow, although the number of detected TB cases remains relatively constant.

I: How is extrapulmonary TB diagnosis being managed?

R: Extrapulmonary TB patients are now being diagnosed more effectively.

I: How is contact tracing conducted?

R: Home visits are conducted regularly, and communication is also maintained over the phone.

I: Do you think the 2025 TB elimination target is achievable?

R: Efforts are ongoing. Proper diagnosis and treatment are continuing, but low socioeconomic conditions, urbanization, and slum populations remain major barriers to TB elimination.

I: What personal difficulties have you faced in implementation?

R: Logistics-related issues include:

- Drug availability gaps
- Delays in supply
- Cartridge shortages
- Patient-related challenges

More counseling is required regarding TPT and laboratory testing.

I: What is your opinion regarding the quality of RNTCP staff?

R: The quality of RNTCP staff is very good.

I: Is stigma related to TB still present?

R: Yes. Stigma related to TB still exists, and people remain scared of the disease. Some patients are even kept isolated by family members, including in educated families. Patients should therefore be properly guided and educated by doctors.

I: Which comorbidities are commonly associated with TB?

R: Comorbidities such as diabetes mellitus, old age, and immunocompromised conditions increase TB risk. Patients with cough symptoms for 3–4 days are screened. During TPT screening, if 100 patients are screened, about 15–20 are found to have comorbidities.

I: Do you have any additional suggestions?

R: Proper and detailed counseling should be provided to TB-positive patients during their first visit. Psychiatric counseling should also be available because depression is common among TB patients.

I: What about newer drugs for TB treatment?

R: If newer drugs become available, treatment will become easier. More new drugs are required.

I: How has MDR-TB management changed over time?

R: Earlier, around 2012, MDR-TB patients were considered very difficult to manage, and healthcare workers were often afraid of these cases. However, now more drugs are available, and MDR-TB can be treated effectively. Depression among patients has reduced, and survival rates have improved nowadays.

I: Are there any newer trends among TB patients?

R: Nowadays, even people working in the IT sector with good salaries are suffering from TB, mainly due to sedentary lifestyles and not taking proper care of their health.

I: What are the current treatment outcomes?

R: Current treatment outcomes are around 80–85%.

I: Any final comments?

R: Maximum awareness is still required in the private sector and among medical colleges.

I: Thank you
