# Supplementary material for: Factors influencing tuberculosis preventive treatment implementation in India: A hybrid inductive-abductive thematic analysis using implementation framework
Source: PLoS One. 2026 Oct 14;21(10):e0359171. doi: 10.1371/journal.pone.0359171 (PMC13667825; doi:10.1371/journal.pone.0359171)
Supplement: Supplementary file 4 [file pone.0359171.s001.zip › In-depth Interview.zip!/In-depth Interview/IDI_6.pdf]

In-depth Interview: 6

Date: 18/12/2023

I: The more frankly and openly you speak and share your experiences, the more it will help us.
Okay, generally, how many areas are under your supervision now? And out of the total PCMC area, what percentage does this cover?

R: Under my supervision, there are a total of six areas.

I: And what is the estimated population?

R: Approximately 2.3 lakh.

I: Generally, how many TB cases are registered every year? Do you have any approximate figures?

R: Considering both public and private sectors together, approximately 3,000 cases are registered annually.

I: Among these, what is the proportion of children? If you do not have exact figures, just a rough idea.

R: Around 5%.

I: And in the private sector, how many patients come?

R: Around 40%.

I: In the last 5 years, do you feel there have been any changes? Before COVID and after COVID?

R: I have recently joined, so I do not know much about the earlier trends.

I: Have you heard about TPT – Tuberculosis Preventive Therapy guidelines?

R: Yes, yes.

I: When did you first come across it?

R: During our training, around two years ago, in 2021.

I: What was mainly emphasized in that training? What things were taught?

R: For TB patients, we were taught to conduct screening for their contacts and also perform IGRA testing.

I: Is the IGRA test now happening routinely?

R: Yes, it has been outsourced. For smear-positive sputum TB patients, the contacts are screened.

I: Do you think the TB elimination target by 2025 will be possible? What is your opinion?

R: The government's approach is very good. They have provided a dedicated workforce, and there is full government support. So, we are working according to their plan.

I: But in your personal opinion, is it possible?

R: There might be a slight delay, but it is possible.

I: What could be the reasons for the delay?

R: Mainly because private sector involvement is still limited. Therefore, some data received from chemists is missing. There are reports that private doctors are diagnosing and treating patients, but they do not enter the details into our system.

I: You mentioned that you receive data from chemists. Do you also get information from pharmacists?

R: Yes. We have a list of chemists, and they provide us with data. We receive complete information regarding which TB drugs are dispensed. This system has been implemented. Reports go to the Commissioners, and accordingly, our staff approaches those chemists. If a chemist mentions that a patient has been given TB medicines for 10 days, then our staff immediately trace that patient. The patient is then included in our system. However, even then, some patients continue treatment without their data reaching us.

I: Approximately how many such patients do you get?

R: Through our campaign and recent survey, we identified only around 1–2 such patients.

I: So, out of the 3,000 cases you mentioned, only 1–2 cases are identified this way?

R: Yes, only a few cases, around 1–2. But there is still a gap and scope for improvement.

I: How is the coverage happening? You mentioned there are six areas. Is there full coverage, or are there any difficulties?

R: The main advantage is that our staff are very experienced. All our TB and MDR-TB staff are fully qualified and have been working for almost 20 years. They work very well. Since I am new, I do not know everything yet, but from what I have seen, they know the complete system thoroughly. However, there has not been much economic growth for them, so there are some issues within the program. They have certain expectations from the government, but those expectations are not being fulfilled.

I: Are there any challenges from private practitioners, and what support do you get from them?

R: Yes. In India, TB notification is mandatory, and accordingly, some support is available from private practitioners. Generally, most people come to us for screening and diagnosis. The challenge is that all MDR-TB patients are treated by us because MDR-TB drugs are not available in the private sector. For

extrapulmonary TB, screening and diagnosis are more challenging. Involvement of AYUSH doctors is also limited.

I: Have they received training?

R: Yes, private doctors have received training, but not all of them attend.

I: Approximately how many private practitioners are there?

R: I do not know the exact number.

I: What about support from the medical college?

R: Yes, there is support from D.Y. Patil Medical College. MDR-TB facilities are available there, along with staff and a Medical Officer. Pulmonary TB patients that we receive are treated there.

I: What proportion of cases do they handle?

R: Our staff is posted there, so they provide data regularly, and admitted patients receive free treatment.

I: Do you think there are any additional challenges?

R: One major challenge is the delayed reporting of cases. Many hospitals lack adequate computer facilities, and staff are dependent on hospital computers. As a result, reporting is delayed, and a lot of time is wasted. Online reporting is a major need. Improved computer efficiency would be beneficial.

I: Has Nikshay training been conducted?

R: Yes, Nikshay's training has been completed. However, training in Excel and Google applications should also be provided.

I: Do you think your clinics and facilities are following health facility guidelines 100%? Or are there any gaps? Also, now that the IGRA test has been outsourced, are drug availability and screening being done on time? What kind of gaps are there?

R: Gaps mainly exist in field work. Patients are usually contacted by phone rather than by home visit. If home visits were conducted, it would be better. Also, due to social stigma, patients hesitate to come for treatment. Newly diagnosed patients are especially very scared. Once treatment starts, the response is generally good, but initially, patients do not come forward easily. After diagnosis, the patient's card is filled, but greater emphasis should be placed on counseling. Each patient should receive counseling for at least 30 minutes.

I: Do you think there are any other gaps?

R: There is limited involvement of ASHA workers. Public health involvement is also limited. They conduct visits only after the patient enters the system, but their involvement in treatment support is lacking. Even screening is not occurring as extensively as it should. At least 5% of new OPD cases should

undergo TB screening, as we are required to report it. If more people are screened, elimination efforts will improve. Screening of diabetic, hypertensive, and elderly patients should also increase. We have recently started focusing on this.

I: Do you have a section for HIV co-infection?

R: Yes, it is available at the ART center.

I: In your opinion, if we want to eliminate TB by 2025, what strategies should be added through the government or public health system?

R: Supply systems need further improvement. CBNAAT services have improved significantly, but the supply chain should not be interrupted. When a patient is admitted, and a sample is collected, the report is expected within 24 hours. If the report is delayed, we cannot proceed efficiently.

I: Was the drug supply proper?

R: Yes, the drug supply is good. Access to drugs has been decentralized to the local level, so generally, there are no major problems. If required, we arrange supplies from the drug store.

I: And regarding prevention?

R: For TB prevention, we currently have limited options. Alternate regimens should be made available.

I: Regarding TPT (Tuberculosis Preventive Therapy), are there any components you feel would help or not help?

R: For TB patients who come to us, in-depth implementation of TPT would be beneficial. Along with the daily regimen, introducing a weekly regimen would have a major positive impact.

I: Now that I have asked all my planned questions, do you have anything else to add or share from your experience?

R: If more personnel, such as ASHA workers and ANMs, are involved, it will help significantly. Doctors generally have limited time, and that time should mainly be devoted to treatment. Other responsibilities can be shared with supporting staff.

I: Do you think cases are being missed? Most migrants may not be seeking treatment at either private or public facilities.

R: Yes, it is possible among the migrant population, although I am not completely sure. Migration is faster in slum areas. ANMs usually visit those areas, but I do not have complete awareness of that system.

I: What can be done for them?

R: Benefits are available for them, but our system needs to become more proactive. Patients should be diagnosed and brought into the system. ASHA workers should be added to support ANMs. If a patient is diagnosed with TB, contact tracing can help identify others.

I: Regarding stigma, what do you think?

R: Nowadays, people have started accepting TB more openly, but stigma still exists.

I: Do you see this in any specific groups?

R: No particular groups, but occasionally, 1–2 patients are found facing such issues.

I: How is compliance?

R: Some patients are started on DOTS treatment. However, due to human nature, once they start feeling better, they stop treatment. Therefore, regular follow-up is necessary. But when follow-up is planned, sometimes their phones are switched off, and no one is available at home. Such cases are seen occasionally.
