# Supplementary material for: Factors influencing tuberculosis preventive treatment implementation in India: A hybrid inductive-abductive thematic analysis using implementation framework
Source: PLoS One. 2026 Oct 14;21(10):e0359171. doi: 10.1371/journal.pone.0359171 (PMC13667825; doi:10.1371/journal.pone.0359171)
Supplement: Supplementary file 4 [file pone.0359171.s001.zip › In-depth Interview.zip!/In-depth Interview/IDI_7.pdf]

In-depth interview: 7
Date: 11-01-2024
(Audio recording was requested, but permission was not granted.)

I: Approximate population and TB registered cases.
R: The approximate population under supervision is about 10 crore. Around 2.26 lakh TB cases were registered this year, compared with about 2.33 lakh last year. For the last 2–3 years, approximately 2 lakh cases have been reported annually.

I: Approximate proportion of children registered.
R: As per norms, the proportion should be about 10–12%, but in our state, it is around 5–7%.

I: Change in the number of cases over the last 3 years, and also pre- and post-COVID.
R: During the COVID period (2021), the number of cases was around 1.60 lakh. After COVID, the numbers increased. The COVID period affected case detection in 2021, but from 2021–22 onward, the number of cases has been higher.

I: When did you first hear about the TPT guidelines?
R: Last year.

I: Training about TPT guidelines.
R: Formal training has not been conducted. We understood the guidelines through the staff. Awareness sessions are being conducted to prevent.

I: Priority-wise key/important aspects in the TPT guidelines.
R: Key aspects include DRTB and MDR-TB medicines, which can help reduce the percentage of TB cases. Contacts of TB patients also need preventive medicine because they are more vulnerable.

I: Please elaborate on each of these aspects.
R: The prevention aspect is important, but side effects are present. Levofloxacin is a potent drug. Nausea and vomiting can occur, and poor adherence may lead to the development of resistance in patients.

I: Achievability by 2025.
R: IGRA testing is being performed in 7 districts, and among those tested, about 40–50% are found positive. However, it is costly, and budget issues exist for testing all patients. Control studies are being conducted in 40 districts. Adult BCG vaccination is also being carried out.

The six groups included for vaccination are:

1. Individuals above 18 years of age
2. Old TB cases (>5 years)
3. Contacts of TB patients
4. Known diabetic patients
5. Individuals with a BMI <18
6. Immunocompromised individuals

I: Extent of coverage.
R: The guidelines are available in medical colleges, but awareness and implementation are lacking in the private sector. Training has been provided. In the public sector, some districts (around 33) are performing well because the Union government has provided additional staff. Some places still have low uptake. Medicine availability is also an issue. Administrative issues are present and need to be resolved.

I: Challenges while implementing the guidelines.
R: Patients need to be mentally prepared to take medicines. Daily INH dosing is difficult, whereas weekly Rifampicin is more acceptable to relatives. Relatives also need to be involved in medication counseling. Patient contact tracing is important, but only about 50% are detected. Follow-up mainly happens when NTP staff actively monitor patients. NGO staff supported by projects have also been recruited.

I: What are the challenges?
R: TB is associated with stigma, which leads to delays in diagnosis.

I: How do you overcome this?
R: General practitioners need awareness. Many prescribe antibiotics initially, and patients return later with a persistent cough. Therefore, early diagnosis is essential.

BAMS doctors should also receive training. People should accept that TB can be cured with proper treatment. IEC (Information, Education, and Communication) activities should be strengthened. The proportion of MDR-TB patients is increasing, and these cases are naturally resistant to antibiotics. Therefore, awareness among general practitioners is necessary. History taking should also improve, for example, by asking about weight loss. State NIMA (National Integrated Medical Association) practitioners need to be sensitized. The program should be properly explained so that they become actively involved. Most districts have already initiated this process. Training is ongoing, and I conducted one six months ago.

I: Is it possible to follow the guidelines effectively?
R: NTP staff should strictly monitor patients; only then will proper follow-up occur.

I: Suggestions for better follow-up of the guidelines.
R: The guidelines are good and are based on proper studies. If proper training is conducted, implementation is possible. Patient self-care should be promoted. Around 50 crore rupees have been received under Nikshay, and the funds are being used properly. The Nikshay portal should be used for monitoring. In about 80% of patients, adherence is maintained, while issues occur in 10–20% of cases. Malnutrition and TB care are directly correlated. Since TB treatment is long-term, counseling is essential for adherence.

I: Opinion about TPT.
R: Preventive drugs are important. Masks should be used, and patients should follow protective measures, similar to those followed during COVID, especially infective MDR-TB patients.

I: Elimination by 2025.
R: The incidence is decreasing. The preventive chain should be properly followed so that the TB burden can be reduced further. Multiple-sector collaboration is needed. Low socioeconomic status groups are more vulnerable. People living in the same household are at high risk.
