# Supplementary material for: Factors influencing tuberculosis preventive treatment implementation in India: A hybrid inductive-abductive thematic analysis using implementation framework
Source: PLoS One. 2026 Oct 14;21(10):e0359171. doi: 10.1371/journal.pone.0359171 (PMC13667825; doi:10.1371/journal.pone.0359171)
Supplement: Supplementary file 4 [file pone.0359171.s001.zip › Focus Group Discussions (Adult contacts).zip!/Focus Group Discussions (Adult contacts)/FGD_AC_2.pdf]

FGD_Adult Contacts_2
Date: 02.05.2024

I: New TB Preventive Therapy Guidelines 2021 have been introduced. Has anyone heard anything about this TB preventive therapy? Do you have any information about it?

R: No.

I: Is there any patient at home? Do people in the household come into contact with a TB patient? Are there any medicines people can take at home to prevent TB?

R1: Tablets are given. I have information regarding this.

I: Is there a TB patient in everyone's home?

R: Yes.

I: Then were any tests done for them?

R: Yes. Chest X-ray and sputum investigations were done.

I: Were the investigations done for everyone at home?

R: Yes, investigations were done for all.

R2: One scan was taken. There was an infection in my daughter's eye. That is why Madam asked us to do the test. In that, TB was found positive. Now tablets have been started.

R3: Testing was done for everyone at home. Chest X-ray and sputum testing were done. Tablets were started for those who were positive, and medicines were also started for the others, so that they do not get TB.

R4: Mother lives separately in a village, and we live in Baramati. We are not in contact with her. Still, they asked us how many days we had been out of contact with her. No tablets or tests were given as we are living separately.

R5: I have a daughter. Earlier, she had malaria, tick fever, and hepatitis. She became very weak, and her cough increased. She was admitted to the government hospital two times. All tests were done, but the treatments were different. The test was done in Khade, Baramati. They said this condition might be TB. Then all investigations and tests were done. Only she was affected. X-ray and blood investigations were done.

I: Were investigations done for any of you from your home?

R5: Yes, they were done. Blood tests were done for all. No one has any trouble or disease. No cold or cough.

I: When the patient was detected at your place, did any of you at home have symptoms?

R6: There was a fever. The fever was not reducing. They gave us Dr. Bora's address and told us to go for investigations and a chest examination. Tests were done for everyone at home. Children's and adults' tests were done.

I: Did they tell the reason behind doing the tests when the tests were done for everyone?

R: Yes.

R1: So that no one else in the family gets infected. That is why they gave information about TB. They came home for inspection. Information was given to all.

I: Yes, but why? Why was the X-ray done? Was information given about this?

R: Yes.

I: Did you know why it was done?

R6: I did not know much. I understood afterward. Then testing was done for all adults, aunts, and even the children at home.

I: Did you know why the tests were being done?

R5: Earlier, I did not know. They explained that after the reports came positive. They told us to keep the patient properly separated as much as possible, keep their utensils separate, and not mix them with others. Now the patient is cured.

I: Was any information given about treatment?

R1: Yes.

I: After testing, when the patient was identified in your home, were medicines started for all?

R1: Yes, medicines were started for all. It is compulsory.

I: Were medicines started for those who were in contact with the patient, apart from the patient?

R2: Yes, they have started.

R3: The voice of the first son had completely gone. Tests and examinations were started. Tablets were started. Two months have passed since the medicines started. Now he is okay. He speaks clearly now.

I: At your place?

R4: Only the patient received treatment.

I: At your place?

R5: Only the patient.

I: At your place?

R6: Medicines were not started for anyone else, but investigations were done for all.

I: Was any information given regarding treatment?

R1: Yes.

I: For how many days did she take medicines?

R1: They told her to take them for 2–3 months.

R3: Tablets were given for 6 months. Medicines had to be taken regularly every month.

I: Were medicines given to relatives in addition to the patient?

R3: Yes, tablets were given for 3 months.

I: At your place?

R5: Tablets were given only to her.

I: Do you have any information regarding the medicines? How are the medicines available? Where do you get them? Are there any side effects? Any information regarding this?

R4: Yes, I got to know from the patient only. If these tablets are started, itching may occur. Otherwise, nothing else. The bones may ache, and weakness may occur, but it will get cured.

R2: Nausea and acidity problems were there. They gave tablets for that also. After that, it was relieved.

R4: They gave tablets for this problem. I took the tablets for 2–3 days, and I felt relieved afterward.

I: Was any counseling done about why it is necessary to start these medicines and complete the whole treatment?

R1: No information was given about that.

R4: Because if we stop in between, side effects can happen. The 6-month course may need to be done again.

I: Sister, what information do you have?

R1: No.

R3: There is joint pain, itching on the body, and burning in the stomach. There is burning in the stomach 1–2 hours after taking the tablets. We gave her 2 tablets at one time.

I: We have started this TB prevention program for TB elimination. In addition to patients, we also give medicines to people who are in contact with them to prevent them from getting TB. From all this, do you feel that TB can be fully eliminated from India? Can we overcome this?

R4: Yes, we can do it.

R4: My mother got TB in 2018. The treatment was done in a government hospital. She was fully cured. Then again, TB occurred in 2023. This suffering happened again. Why does this happen?

I: We will talk about this later, but what is your opinion about this?

R4: Yes, it can happen.

I: What do you feel? Can we overcome TB?

R5: Yes, if we take proper care, then we can overcome it.

I: What do you feel? Can we eliminate TB completely?

R6: It can happen.

I: What are your experiences regarding TB treatment?

R3: We take our tablets on time every day. Now everything is okay.

I: Were there any difficulties during investigations?

R1: No.

R3: No.

I: When was TB detected?

R1: It has been 1 year since TB was detected.

R2: 1.5 months ago. Can an eye infection happen due to the tablets?

I: Yes, it can happen.

R3: 5 months ago.

R4: 2 months ago.

R5: 3 months ago – recurrence.

R6: 9 months ago.
