# Supplementary material for: Factors influencing tuberculosis preventive treatment implementation in India: A hybrid inductive-abductive thematic analysis using implementation framework
Source: PLoS One. 2026 Oct 14;21(10):e0359171. doi: 10.1371/journal.pone.0359171 (PMC13667825; doi:10.1371/journal.pone.0359171)
Supplement: Supplementary file 4 [file pone.0359171.s001.zip › Focus Group Discussions (Adult contacts).zip!/Focus Group Discussions (Adult contacts)/FGD_AC_3.pdf]

FGD_Adult Contacts_3
Date: 15.05.2024

I: Now there is a case of TB in everyone's home, right? Then this case came to light, and the diagnosis was made. Was anything said after that regarding its treatment or any related information? Do you want to say anything regarding this?

R6: Yes, I was told to take these medicines on time. Along with that, eat leafy vegetables and foods from which protein can be obtained, such as eggs or beans.

I: Now this is about the patient, but what about you taking care so that the disease does not happen to you?

R: Maintain less contact. Actually, there were lesions. Everything will become normal gradually. There is also a small baby.

R: Then they said there is no problem. There are two lesions.

I: Was anything told to the rest of the family members by the doctor or nurse?

R4: My experience is that after the neighborhood people came to know, they started looking at us differently. The situation became such that we felt ashamed even to take our sister anywhere. After coming here, the doctor helped us a lot. My expectation is that instead of distancing themselves, they should encourage the patient. Nothing happens by frightening the patient. They should give confidence and support. Such behavior troubles me. If this happens for the first time, it is the doctor's responsibility to reassure the patient that nothing will happen. But we did not get that support at all. We are from Akkalkot, Solapur proper. Another thing is that we live here on rent with my two small children. Even if we look for another room, no one is willing to give us one. I feel awareness should be created regarding this disease, that if proper treatment is taken, the patient will get cured. The condition became very bad. Even now, if we see someone nearby, we hesitate because of what people might say.

I: Were medicines given to any of you?

R8: Yes, they were given.

I: Who received the medicines? I am not talking about the patient now. I am asking about you as a relative of the patient and your experiences.

R: Madam said that my husband had a cyst. They were about to start tablets for him.

I: Okay.

R9: They were about to start tablets for family members in the house. One tablet a day to prevent the disease from spreading. But they have not given them yet.

I: Not given yet?

R: No.

I: One tablet every day. When did your patient’s diagnosis take place?

R9: 6 months ago.

I: No, no. I wanted to know how many months ago it happened.

I: Then no medicines were given in these 6 months?

R: No, nothing for us.

R8: People came for a survey. They gave tablets. A survey was done in everyone’s home.

I: Okay. At your place also, were they not given by anyone?

R7: It happened just now. It is only starting now.

R7: She is having trouble because of those tablets.

I: Then did you tell this here?

R7: No. I came now, and then auntie said that earlier she was 48 kg. She eats a lot. She is physically strong also. Non-vegetarian food is compulsory for her. Her father said not to avoid it even on Tuesdays. That is why non-vegetarian food and fish were started for her regularly. Over the last 2–3 days, her cough has worsened, and she has not been eating on time. After taking tablets, the food is not being digested, and she is vomiting. Nowadays, she is facing a lot of trouble.

I: We will talk about this later. Right now, we are talking about the patient’s relatives, not the patient. You tell about yourselves. Many of you got tablets. Did you take those tablets?

R8: Yes.

I: For how many months did you take them?

R10: 3 months.

I: You took them only for 3 months?

R10: 3 months.

I: Were the tablets given to you?

R: They were not given to us.

R: They were given to the patients.

I: Okay, for the patient. You took 3 months. Brother, did you get them?

R6: No.

I: Sister, you?

R: I did not get them.

R: Because they said there was no necessity for you because of your condition.

R: They were really good. They used to call themselves and ask if there was any problem. At that time, the weight was 49 kg, and now it is 54 kg. That is why I say this.

I: Wait, we will talk about this later. We will clear all your doubts at the end. Sister, you did not get any tablets. You took them for 3 months. Okay, now those 3–4 people who got tablets — what was told to you by the ASHA worker, nurse, or sister? Did they tell you why you should take these tablets?

R10: They said it was for immunity so that it does not become weak. They said we would not get infected by contact with the patient. The disease should not happen to us again. Because of this, our meal intake, diet, and movement outside should not be reduced.

I: What else did they tell?

R8: They said it should not spread to us. If it has happened to one person, it should not happen to another.

I: Were investigations done for any of you? Such as sputum or blood tests? Was everyone’s sputum test done?

R8: Yes.

I: Was anyone’s X-ray done?

R10: No, only sputum examination was done.

I: Okay. Were you told for how many months the tablets should be taken? You got them for 3 months.

R: It was told for 6 months.

I: Not for the patient, for the relatives.

R4: It was told for 3 months.

I: Okay. Earlier, you were talking about your experiences in society. Do you want to share those experiences as relatives of TB patients?

R7: Leave aside relatives. Doctors should also understand a little and cooperate. The main thing is the doctor, because the patient depends on them. How much can patients tolerate? If others say something, it may not matter, but the doctor should not behave like that.

I: Okay.

R7: They say, “Wait aside, stay there.” That should not happen. It should not be one-sided. This is my opinion.

I: Does anyone else want to say anything or share any experiences?

R: We admitted him to Bharati Hospital. Even at night, they did not come close to him. They did not touch him and behaved distantly. The same thing happened there too. He used to cry a lot because of this behavior. Finally, after 15–20 days, we brought him back home. After that, treatment started at home, and later at another place where they took proper care of him. We used to send tiffin from home. Mother used to prepare the food. We did not allow him to go outside the house for 3 months because there were small children at home.

R: Why do doctors behave like this? Why does this happen?

I: We will talk about this later. But do you want to share any experiences or difficulties you faced, and what should be done to make things better?

R5: Sometimes we do not get tablets on time. There is a 15-day delay, and then we have to buy tablets from outside. The tablets that are supposed to be available here are sometimes unavailable for 15 days.

I: Any more difficulties? Do you want to talk about them or share any experiences?

R: I did not tell anything to anyone because he had holidays and was at home only. Now his school will start after June. How should we take care of him? Till now, he had only been at home.

I: Do you want to share anything else? Do you feel that if something is done differently, the patient will feel better?

R6: When there is a patient, we should treat him with love and care, just as a mother treats her son lovingly. If we treat the patient that way, the patient will recover better.

R4: This is my personal opinion. The patient loses confidence because of such treatment. Relatives demoralize the patient more than anyone else. In many places, relatives demoralize the patient more than the disease itself.

R2: After my wife came to know that 10 children had TB, she started crying. The doctor explained properly that this disease can be completely cured. This created confidence and strength.

I: Okay. Does anyone among you have high blood pressure or diabetes, or have any such investigations done?

R: No.

I: Okay. In your homes, there may be elderly people because these relatives are from your homes only, right? Senior citizens may have high blood pressure or diabetes. Is there anyone like that?

R6: My mother is there. But when our treatment started, we kept a distance from everyone. My mother and son stayed as far away as possible, and I stayed outside. While going out, I used to wear a mask myself.

I: Okay.

I: Are there any children below 5 years in your home?

R: No.

I: Do you want to say anything more?

R6: I wanted to ask one more thing. Everything is okay, but the baby is 2 years old. There is no problem for the baby, right?

I: We will talk about this after some time. Does anyone want to say more?

R: They gave me tablets so that the disease would not happen again a second time.

I: When was the case diagnosed in your home? When did it come to notice?

R: My daughter was in the 12th standard at that time.

I: Tell me how many years ago.

R10: One year back, in September last year.

I: At your place?

R8: Around 8–9 months ago.

I: At your place?

R: Around 15 days ago.

R7: One week ago. There was a small child.

I: At your place, brother?

R2: First, there was a small cyst. I noticed it, but I thought it was normal. Gradually, it began to increase and grow. I noticed it properly after 4–5 months. Then I showed it to the doctor. The doctor started treatment for it. After one month, the cyst dissolved completely. After testing, we came to know it was TB.

I: Brother, at your place?

R: Last time, during the cold season.

I: Brother, at your place?

R3: 2 months ago.

R4: 6 months ago.

I: Then is their treatment completed?

R: Yes.

I: Were investigations done again?

R: Yes, they were done. Nothing like that was found again.

I: Sister, you?

R5: 8 months ago.

R6: December, sorry, in November 2022. The course was completed in April.

R7: My son had stomach pain when he was in the 9th standard. We showed him in the hospital. They gave tablets for stomach pain. His stomach used to ache a lot when he was in the 10th standard, also.

I: Did he have trouble in the chest or stomach?

R: Stomach pain. Mild pain at times.

I: Okay. Was the illness related to the chest or stomach?

R: Stomach.

I: Near the stomach?

R: We went to the hospital. After doing the biopsy and removing the stitches, only then, after 6 months, did we come to know through testing.

I: Then he should have been checked more thoroughly to see whether this was present or not.

I: Okay. Those who got tablets, did you take them regularly for 3 months? Or did you take them irregularly? Were the tablets given to you regularly? Did you receive them every month or all together for 3 months?

R10: No. I used to get them in my daughter’s name whenever I collected his tablets.

I: Were they given collectively for 3 months?

R: Yes.

I: Who gave the tablets?

R8: Sassoon Hospital.

I: Okay, Sassoon. Did you take the tablets by going there or here?

R8: No, they came home. Everyone came. They used to bring them home every time.

I: Did you also get them? Did you receive them every month after coming here?

R4: No, from Swargate.

I: Okay. Did you have any difficulty taking those tablets?

R: No. They said if there was any difficulty, we should come and tell them.

I: But did anyone have any difficulty?

R: No.

R: The small child takes tablets from there, but sometimes vomiting happens frequently. Should we give another tablet after he vomits?

I: How old is the child?

R: 4 years.

I: Does anyone want to say more? Did the rest of the family also get tablets?

R: Yes.

I: Did everyone get them? Who did not get them? These 3 homes are there. You did not get them, right?

R: They were started only for the patients.

I: Yes, but they were not given to you, right? Only 3 members said they got them. The rest did not get them. Did everyone at home receive them?

R: Yes.

I: For 3 months each?

R: Yes.

I: At your place also?

I: Now we will stop here.

THE END!
