# Supplementary material for: Factors influencing tuberculosis preventive treatment implementation in India: A hybrid inductive-abductive thematic analysis using implementation framework
Source: PLoS One. 2026 Oct 14;21(10):e0359171. doi: 10.1371/journal.pone.0359171 (PMC13667825; doi:10.1371/journal.pone.0359171)
Supplement: Supplementary file 4 [file pone.0359171.s001.zip › Focus Group Discussions (Adult contacts).zip!/Focus Group Discussions (Adult contacts)/FGD_AC_4.pdf]

FGD_Adult Contacts_4

Date: 28.05.24

I: Have you heard about Tuberculosis? If you have, then have you heard about TPT? How can you prevent TB? Have you heard about how to prevent others from getting infected?

R-1: I have heard that if contacts get tablets, they will recover in 5–6 days or 8 days. Treatment is for 6–7 months; some cases have a 9-month treatment, and some also have a 3-month treatment. It depends on how severe it is.

I: Have you heard about this?

R-1: It has many types, such as lung TB.

I: Okay. Have you heard about how to prevent it?

R-4: No.

I: Have you heard about this?

R-5: No.

I (Asking R-6): Have you heard about how to prevent others from getting infected?

R-6: No.

I: So, as you said, what should be done so that others cannot be affected?

R-3: I will receive my brother’s reports today. Fifty years ago, my grandmother had TB. My mother said that such patients should be quarantined for 8 days after the start of treatment to prevent others from becoming infected. Such patients should wear masks. That much I know. Doctors have not said anything yet.

I: Okay. Has anyone diagnosed you or your family? How many times have they done it? If the patient has been diagnosed, then treatment has been started. But

have any other members of your family, including you, had tests such as chest X-rays, sputum tests, or blood tests?

R-4, R-5: No.

I (Asking R-6): Have you done any blood or other tests?

R-6: Yes, done.

I: Has everyone in your home done it?

R-6: One more thing was also given with that.

I: What was that?

R-6: My husband received that.

I: Okay, that means you did not receive it.

R-6: Yes, they said so.

I: You received it only in Nepal; nothing has been said at your current location?

R-6: They said that if eating and drinking are ignored and medicines are not taken on time, then it happens.

R-4: The information we want is not provided to us. When we came to the hospital and received treatment, nobody told the family members that they could also suffer from TB because of the patient. No such information was received.

I: When did it happen?

R-4: It has been a month now.

I: A month? Okay, are the medicines ongoing?

R-4: Yes, medicines are ongoing.

I: When did your husband and mother-in-law get it?

R-5: It has been a month. My husband started taking the medicine for six months, and he started it last month. Then we went to the village; after that, we came here and restarted it, so it is ongoing now. My mother-in-law has had it for 4 months, with 2 months left.

I: Haven't taken it for two months?

R-6: She went to the village, so she missed it.

I: Just restarted? How many months has it been since it restarted?

R-6: It has been a month; she has taken it twice.

I: Yes. (While asking R-1) At your house? How many months has it been for the TB patient, your wife?

R-1: The treatment started in February. A biopsy and an MRI were performed.

I: Okay.

R-1: Nothing was detected in the biopsy.

I: Okay.

R-1: And nothing was detected in the MRI.

I: Was it done for the chest or something else?

R-1: It was for the spine.

I: Okay, it was for the spine.

R-1: They said that a three-month course of treatment should be done.

I: So it has been three months since you started taking it?

R-1: After two months of continuous treatment, bilirubin levels started increasing. Then there was a short break.

I: For how many days was it not taken?

R-1: 15–20 days.

I: It was not taken for 15–20 days. Are medicines available now? Have you received the patient's medicine?

R-1: No.

I (Asking others): Have you received the patient's medicine?

R-2 to R-6: No.

I: Okay. None of you has been given any medicine or undergone any tests, right?

All respondents: No.

I: So you know this; just like COVID is a disease, this disease is also somewhat similar if everything is explained properly. But can you please tell me how to take care of it?

R-4: We do not get enough information, so we have no idea.

I: Does ASHA come to your area?

R-4: One ASHA worker comes from Varulwadi. She once came and said they come every month or every three months to check. She asked, "Do you have any such contacts here?" Our neighbors were so patient, so I told her. The patient did not say anything, even to her children, and later she expired. She did not take the pills on time. She was living right next to us.

I: Does someone like an ASHA worker come to you and tell you what to do and what not to do?

R-5: They should have given more information.

I (Asking R-6): Does anyone come there like an ASHA worker from the government? Where do you live?

R-6: Where?

I: At your place, around the house, for surveys, or to ask something?

R-4: No one comes.

I: Okay. Can TB be reduced?

R-1: Yes, it can.

I: What is your opinion?

R-3: My grandmother had it and got cured after TB treatment. At that time, they said that it could return.

I: Do you want to share your opinion or something that happened in your house regarding this disease?

R-5: No, nothing happened, so what can I say?

I (Asking R-6): Do you want to say something? Your husband and mother-in-law had it; do you want to say something? Did anything special happen during this disease or something like that?

R-6: The cough was present earlier, but it gradually reduced after he started the medicine. Nothing is happening right now.

I: Nothing is happening right now. (Asking others) Does anyone else have something to say or share any experience?

R-3: My brother has diabetes. His sugar levels were increasing, and he had a cough for a long time. Even after 2–3 months, the cough did not go away. He took the doctor’s medicine regularly, but it did not make any difference. Then we went to Pune to the doctor who treated his diabetes. The doctor started insulin because his sugar level was 350. I did not know exactly what the issue was; there was only a cough. Dr. Kanse at Alephata said, “Get an HRCT done and check the sugar levels. If there is a problem later, the cough will not resolve because

elevated sugar levels are associated with disease. If you are positive, then take treatment from a government hospital." The report has not come yet; it will come today.

I: Okay, does anyone want to say anything?

R-4: We do not want to trouble these people. We tell society that there is a cure for this disease. We do not want to trouble that person mentally; he should feel completely healthy and fine. If family members pressure him by saying, "Do not eat this, do not eat that, it can spread to you or others," then he feels mental pressure. If the patient is already taking treatment, then why should society trouble them? It should not be said that "you should not be in contact with him." The disease is being treated with medicines, so why should people say such things to them? We do not want to give them mental stress. That is all; I have nothing else to say.

I: Does anyone else want to say anything? Did you want to say something to someone?

R-6: Wishing everyone a speedy recovery and sending blessings to all.

I: Let us stop here. Thank you very much, everyone.
