# Supplementary material for: Factors influencing tuberculosis preventive treatment implementation in India: A hybrid inductive-abductive thematic analysis using implementation framework
Source: PLoS One. 2026 Oct 14;21(10):e0359171. doi: 10.1371/journal.pone.0359171 (PMC13667825; doi:10.1371/journal.pone.0359171)
Supplement: Supplementary file 4 [file pone.0359171.s001.zip › Focus Group Discussions (Adult contacts).zip!/Focus Group Discussions (Adult contacts)/FGD_AC_5.pdf]

FGD_Adult contacts_5

Date: 05.06.24

I: Do you know anything about preventive treatment for TB?

R1: Means?

I: This means there was a patient in your house. Do you know anything about TB preventive treatment?

R2: Treatment means that his 6-month course is now completed, and they told us that we should also take the pills.

I: When did this TB treatment take place? Was it 6 months ago?

R4: It has been 7 months now.

I: Okay. How many people are in your home?

R4: Three.

I: So, who underwent the tests?

R4: Both of us, and the children also.

I: Were they checked?

R4: Yes. We had no problem taking the pills. We had no trouble while taking the medicines.

I (Asking R5): Now, who is the patient?

R5: Husband.

I: Since when has the treatment been started?

R5: From the 12th date.

I: Has it started now?

R5: No, 6 months have passed.

I: Six months have passed. Have your family members undergone tests?

R5: Done for three people.

I: Which tests were done?

R2: We started those pills so that the disease would not happen.

I: Yes, but can you say the name of any test?

R5: No, unable to say.

I: But did they explain which test it was or what its purpose was?

R5: They said it was so that the disease would not happen. I underwent the tests and took the pills. My mother-in-law and two children also took the pills.

I: Means all have taken pills.

R5: Yes, every month we went together.

I (Asking R3): Who was the patient?

R3: She.

I: For how long has the treatment been ongoing?

R3: For the last 6-7 months.

I: Who underwent tests from your home?

R3: Her husband and son underwent tests. Pills were started for the son but not for the husband. The son took pills for 5-6 months; after that, he stopped. His treatment was under Dr. Kadam. Dr. Kadam gave a prescription and asked us to mention the doctor’s name while taking the medicines.

I: Which test? Name?

R3: Unable to say.

I: Means blood test or X-ray?

R3: X-ray was done. A chest X-ray was advised, and the reports were normal. But for prevention, the pills were started. She and her son were taking the pills only.

I: Did they say for what purpose the medicines were given?

R3: We understood that they were given for prevention now and in the future.

I (Asking R2): Who is the patient?

R2: Wife.

I: Since how long has she been suffering?

R2: For the last 6-7 months.

I: Has she taken all the medicines?

R2: Yes.

I: Have your family members undergone tests?

R2: Yes, they did.

I: How many of them underwent tests?

R2: 5 members.

I: 5 members, which means all underwent tests. Any idea which tests were done?

R2: Blood was taken.

I: Okay. Of them, how many started taking medicines?

R2: Medicines were started for three of us. One 1.5-year-old child was also taking pills.

I: How long have you been taking the medicines?

R2: We completed them. We took them for 6 months.

I (Asking R1): Who is the patient?

R1: Daughter.

I: Since when has she been taking treatment?

R1: Started from December 2022.

I: Have tests been done for family members?

R1: Yes.

I: Have all undergone tests?

R1: Yes.

I: Which tests were done?

R1: Blood was taken.

I: For all?

R1: For two of us.

I: What did they say to the two of you?

R1: Red-colored tablets, meaning calcium tablets, were given. Tests were done to check for TB. The reports showed no TB, so calcium tablets were started.

I: When the tablets were started, what did they say?

R1: Use a mask, maintain distance, and complete the treatment. If any difficulty occurs, contact us here.

I: Okay. (While asking R2) Have you, the contacts, or others experienced any problems or side effects from the medicines?

R2: No, none.

I (Asking R3): Have you or your husband suffered from any side effects?

R3: No.

I: Okay.

I (Asking R5 and R4): What about you?

R5: None.

R4: None. Eating plenty of food does not cause any side effects.

I: As you know about TPT, and all of you have undergone tests and received treatment, do you think that TB can be eliminated, or is TPT beneficial?

R4: None of us suffered from the disease, so it is beneficial for contacts to take medicines. Whether the reports are positive or negative, medicines can be taken by all so that no one gets affected.

I (Asking R5): What about you?

R5: I also think it can be treated by using medicines.

I: Can TB be reduced because of this?

R4: By cleanliness, it can be prevented.

I (Asking R3): What do you think?

R3: Same.

I: Does anyone want to say anything about TPT or the tests?

R: No.

I: Have you received medicines for 6 months?

R: Yes.

I: Means you received all the medicines and no medicines were missed?

R: No.

I: So, thank you all.
