# Supplementary material for: Factors influencing tuberculosis preventive treatment implementation in India: A hybrid inductive-abductive thematic analysis using implementation framework
Source: PLoS One. 2026 Oct 14;21(10):e0359171. doi: 10.1371/journal.pone.0359171 (PMC13667825; doi:10.1371/journal.pone.0359171)
Supplement: Supplementary file 4 [file pone.0359171.s001.zip › Focus Group Discussions (Adult contacts).zip!/Focus Group Discussions (Adult contacts)/FGD_AC_6.pdf]

FGD_Adult contacts_6

Date: 18.06.24

I: Have you heard about TB preventive treatment, or do you know something about it?
R: Heard about it.

I: Then, who is the patient?
R3: Sister.

I: When did the diagnosis take place?
R3: She has been hospitalized for 4–5 days now and has been informed that medicines are available here.

I: So, now the diagnosis is done?
R3: Yes.

I: Who lives in your house?
R3: Sister-in-law, brother, me, uncle, and sister.

I: Ok. Have you all undergone TB tests?
R3: No.

I: No one in your house has done the tests. Have they informed you about how to prevent it?
R3: No.

I: Have medicines been started for all the remaining family members?
R3: No.

I (Asking R2): Who is the patient in your home?
R2: Uncle.

I: How many members are in your family, and who are they?
R2: 7–8 members.

I: When was he diagnosed?
R2: Not diagnosed yet; treatment is ongoing.

I: How long has he been taking medicines?
R2: Medicines have yet to start.

I: Have your family members undergone diagnostic tests?
R2: No.

I: Have TB tests been done?
R2: No.

I: He is from your family. Have the family members undergone diagnostic tests?
R2: No.

I (Asking R1): Who is the patient in your family?
R1: Husband.

I: How many members live in the family?
R1: 3.

I: Have they undergone tests?
R1: No one.

I: Have all undergone a sputum test? Have they explained why it takes place?
R1: For TB.

I: Has any other test been done along with that?
R1: No.

I: Did medicines start after testing? And when was he diagnosed?
R1: No.

I (Asking all): "How do you prevent TB in a home with one patient suffering from TB?" Do you know about it? What do you say about it?

R4: If medicines are taken on time, they can be prevented.

R5: Same.

I: Does anyone want to say anything, share any experience, or mention any difficulties?
R: No.

I: Thank you for your participation.
