# Supplementary material for: Factors influencing tuberculosis preventive treatment implementation in India: A hybrid inductive-abductive thematic analysis using implementation framework
Source: PLoS One. 2026 Oct 14;21(10):e0359171. doi: 10.1371/journal.pone.0359171 (PMC13667825; doi:10.1371/journal.pone.0359171)
Supplement: Supplementary file 4 [file pone.0359171.s001.zip › Focus Group Discussions (Adult contacts).zip!/Focus Group Discussions (Adult contacts)/FGD_Part I_AC_1.pdf]

FGD_Part I_Adult Contact_1
Date: 30.04.2024

I: If someone gets contracted with TB, then it is curable, you know this right? If you take medications properly and eat properly, then it can get cured. The person can become completely healthy again. Right? Along with that, it cannot be ignored that there is a possibility that his/her family can also get affected. Right? Then for that, it is important that family members should get some medicines. Do you know that?

R: Yes.

I: Okay. What have you heard about this? What information do you have regarding this? Tell me.

R1: Whatever happened 3 months ago, we completed its treatment. Tablets were given at that time.

I: Okay. Given for how many months?

R1: Given for 6 months.

I: Then, at that time, what instructions were given to you? How was it at your place?

R2: They told us that we should not come into much contact.

I: Who got the disease in your home?

R: My grandmother.

I: Grandmother.

R: She said, “Will we not be able to manage?”

I: Okay. What more information do you have regarding whatever was happening with them?

R: The patient was brought here. There were no other issues. Utensils for eating were kept separately for one person only. No one else from the house was affected.

I: Okay.

R: But we followed whatever was told to us.

I: Were any investigations done for you?

R: Yes, they were done. Sputum was taken. Blood was also taken.

I: Okay. What about you?

R2: A blood test was done.

I: Okay. Whose blood or sputum investigations were done from home?

R1: Everyone's.

I: How many people from home?

R1: 3 people.

I: Are children included?

R1: They are adults only.

I: In your home?

R2: 3 people in my home.

I: Was everyone's sputum investigation done?

R: Yes, yes.

I: Was everyone's blood taken?

R1: It was taken here only. Everything was done.

R: It was taken here itself.

I: Was it taken here only?

R: That madam was there.

I: Okay. Do you have any information about why it is necessary to do these investigations and take medicines?

R1: So that we should not get infected from others.

I: Okay. Was this told to you?

R: Yes.

I: Who told you?

R1: Doctor.

I: You?

R: Hospital staff. We came by ourselves.

I: Are you from the same house?

R: No, different.

I: Different.

R2: They did tests after bringing everyone together.

I: Okay. How many tablets did you take?

R2: I took them for 5 months.

I: You?

R1: 6 months.

I: Okay. Who used to give you these tablets?

R: The sister madam from here.

I: Did she give them by coming here or at home?

R: No, they gave them here. Our tablets were about to finish weekly, so they used to call us. We also used to inform them by calling.

I: Your tablets were different, and theirs were different?

R: Yes.

I: Means relatives?

R: Yes, tablets for relatives were different and ours were different.

I: Okay. Did you have any difficulty while taking those tablets, any trouble?

R: No, no, nothing. One tablet every evening, one time.

I: Okay. Did anyone guide you regarding this, maybe ASHA worker, doctor, or sister, about for how many days you would have to take these tablets?

R: They used to explain everything by coming home.

I: Okay. Does anyone among you have blood pressure or sugar problems?

R1: No. Nothing. No one.

I: Did anyone else from the house have it?

R: No.

I: Then you took these tablets, you for 5 months and you for 6 months?

R: Yes.

I: Other people from the house?

R1: There are 3 people. Everyone took one tablet. Ours was different. Every evening one tablet between both of us, and the patient's tablets used to be different.

I: How was it at your place?

R2: At our place, she had a different room. That is why we used to give her food separately, and we used to give tablets every day.

I: How many of you used to do this?

R: We are 4 people.

R: Yes, brother and others.

I: Did they take those?

R: Yes.

I: Okay. The remaining 3 people in the family?

R2: Yes, everyone took them.

I: Did it ever happen that tablets were missed or something?

R: No, no.

I: There are many efforts ongoing so that Tuberculosis gets fully eliminated from India. You might have heard this in the news or on the radio. Then what do you feel? Can it happen? What is your opinion regarding this?

R1: It can happen.

R2: If we follow everything properly regarding this.

I: If we follow it. Okay.

R: This is not such a big disease. It will definitely get cured.

R1: We should ensure proper eating habits and proper intake of tablets, then anything is possible.

I: Did you have any difficulties regarding taking tablets?

R: No, nothing happened.

I: Taking them every day for 6 months — did you feel any difficulty or get tired? Did anything like that happen?

R: No, no.

I: After 5 months, did you stop taking them by yourself, or did the doctor tell you?

R2: No, the doctor told me. I was in Pune for doing MBA. I came home for one month. Then they told me.

I: You did not stop on your own?

I: Do you want to tell any of your experiences regarding this?

R: This treatment was going on properly. The treatment was proper. Now there is a 100% difference. No difficulty at all. We took the treatment properly and completed the course, so nothing happened. Since the whole treatment was completed, and we took the tablets and meals on time, everything became completely clear now.

I: Okay. Did any investigations take place again after 6 months?

R: Yes, I got them done once again. I took the doctor's consultation.

I: Which investigations were done?

R: I went to Sassoon Hospital and showed the reports to the doctor. They said everything seemed fine. After that, nothing happened. Now, even if anything minor happens, I immediately go for a check-up.

I: Were your investigations done again or not, like blood investigation or sputum checking that were done earlier?

R: Investigations were done.

I: When did you get the investigations done?

R: In the last few months, around one year later.

I: Means how many months after taking medicines?

R: In 6 months.

I: Okay. What about at your place? Your father?

I: Okay. Do you know anything about family investigations?

R2: Yes, investigations of family members were done.

I: They were done.

R: Even if you feel a little discomfort, you should get checked. Everything is completely clear for the patient now. No difficulty.

R1: Tablets were taken on time.

R: Now he does not have cough or fever. Food intake is proper.

I: Do you want to share any more experiences? You, brother?

R: No.

I: Okay. Thank you!

THE END!
