# Supplementary material for: Factors influencing tuberculosis preventive treatment implementation in India: A hybrid inductive-abductive thematic analysis using implementation framework
Source: PLoS One. 2026 Oct 14;21(10):e0359171. doi: 10.1371/journal.pone.0359171 (PMC13667825; doi:10.1371/journal.pone.0359171)
Supplement: Supplementary file 4 [file pone.0359171.s001.zip › Focus Group Discussions (Adult contacts).zip!/Focus Group Discussions (MO)/FGD_MO_1.pdf]

FGD_MO_1

Date: 18.04.2024

I: Sir told that Tuberculosis prevention related guidelines are released in 2021. Then you might have heard about this. You might be knowing; then what information you have? We would like to know it.
R: Actually we are giving TPT TB Preventive Therapy to close contact to those whose sputum index case have sputum positive pulmonary TB. We give mostly to them. And we give INH 300 mg for the 1st 6 months because now only it is available. Best available treatment and it is for 6 months. We won't take contacts till we are taking index case. And 1st upon before starting whatever symptoms are there we want to do TB screening before. If found negative, then we can start otherwise we have to investigate them if they have symptoms then they have to give. I want to say just this.
There is a shortcut in this. H + B and Plus Rifampicin and if they are resistant. Suppose. We can add Rifampicin. We are giving to sensitive TB patient contacts otherwise we don't give.
Found. I say whatever it may be. If there is resistance, there are changes in it. Which are advanced.
I: Does anyone want to add more?
Ma'am TPT Tuberculosis Preventive therapy before Tuberculosis or after?
R: Most important is we should know the symptoms. What for what. Symptoms like cough for more than two weeks, fever at night, food most important, loss of weight, loss of weight, loss of appetite. Means we should do the surveillance for the people having cough more than 8-10 days via ASHA. Is there a cough? Are there patients with cough? We can do survey.
R8: Then we can do adding in that. Loss of weight, then adding adding, they have a cough for more than two weeks, then attention should be given to them. In those which are contacts, 2000 date, 30 reasons then then their sputum microscopy again, True NAAT etc and if a multidrug resistance patient is there then may be contacts' then little bit then may be they can get resistant. It is that.
R2: I have one female case, means of active reproductive age. She has two small children. Earlier she used to live in a nuclear family at Mumbai side. Now she has shifted to her maternal side. TB means the tab;lets are again started for her from 1 month, she ate that there and then her household contact. Then what happened when she was at her in-laws place at that time also her contact came. Aurangabad to Mumbai side and now she is here. Then whom to give TPT, it should be given here also and there also. Means contact of a TB patient for 1 month or 2 months. It will be better if you elaborate how to decide whom to give TPT. Really it is a question whether to give at both places because her brother also has children.
I: We will talk regarding that at the end. At this time it is more important to know your opinions. Okay. Does anyone want to say more regarding guidelines? Namrata, do you want to talk about this?

R10: We try to start TPT to close contact at first possibly. People's mindset is such that we won't take treatment unless I get some symptoms. And how is it to start without parents if it is biomedical. Means compliance is issue in TPT. Now how will happen because of this

I: Means if he is not ready to come, then

R2: If we convince them. They used to leave in the middle after starting, there are chances. Because anyone is sensitive in case of little kids.

I: now you have come towards implementation then we discuss regarding this. Then how to do implementation and then we will have difficulties. Then what things should be done? You told some things while back during TPT implementation like screening. But we will talk in detail.

R: Yes ma'am

I: Then tell me what things to be done during implementation, which steps should be taken?

R3: First of all, it is important to educate our whole staff, mainly ASHA workers and we can include Anganwadi workers because they are more close to children. If we educate them then it will be more easy. Means there is a problem with children like if they are in household contact, then it will be easy to tell them to give or not to give. We can tell during vaccination also because many outlet stations happen at Anganwadi and second thing is if political interference is increased then that will also be easy or if influencer people in those villages spread that it is necessary to give this, then it will be easy to give. And if these things are happening or they are saying no, or why all these things will be easy.

I: What about you sir?

R4: Whatever people who had TB or they had complications, awareness should be done in their relatives or other patients and we should give confidence. Means I feel it is necessary to pursue them what is prevention, and it is important.

R2: In my observation, awareness about TPT is little less. We have done so in case of TB. Check 2 weeks before and you should check sputum. As much as this is imprisoned in people's minds, THAT much is not observed in case of TPT. Means agree that it is started now, but we have to impact. Many people don't know how to do prevention. They know what to do after it has happened or whatever it is. But many don't know about TPT.

R6: It doesn't happen much here. If we involve teachers then if education is there

R3: Means it was done for us similarly if done for teachers then it will be easy. Because it is told in school because if we go to each student's house or there are no side effects of this, it is better for us. It will be good.

I: Anything more? You did not say anything

R5: It should be like whatever opinions were put up just now. The first and most important thing is awareness. TB disease and it;s treatment and caution gossip on that. We search TB patients but instead of searching if we give proper treatment to whatever close contact then household won't happen. And alternatively it can happen through each other. Means awareness will be through education, from us means if media is there then via that. This will have benefits.

R8: And to reduce the Goal of relatives, eradication means we in prevention means should I tell, if we give 100% TPT in a proper manner in prevention, then there are chances. It is our role.

R7: If elderly people, little children are at one place then chances of infection are more for little children. They are made more aware of that. Fever, telling in a way whatever your children are. IOt is necessary to tell these things properly to ASHA for that.
R8: If there are side effects of that, then it is necessary to take them in confidence that some trouble with that. It is necessary that proper screening should take place for contact. Do they have any illness, comorbidity? What is their situation? It is necessary to screen these things also via medical officer.
I: Now you all are medical officers. Each one of you has a specific area. Then will you be able to tell how many contacts are traced in that area. Don't need numbers but can tell in percentage. Approximately how many contacts will be traced?
R1: Mostly we try to do 100%. Mostly household are done but their workplace, etc. are more according to my opinion. Means I feel till now I started TPT for as many as from that there are in their place also. We are covering the household in that but maybe we miss around them.
R2: In our guidelines also, we say close contact but workplace is not included anywhere. In guidelines also, there is one thing to give them or go and convince them also, or you give information to them. And second thing is we are doing eradication, which means we are doing prevention also and giving treatment also then somewhere it will run simultaneously, for that we will have to make guidelines because we are giving TPT somewhere things are missing.
I: how much percentage of contact is there in your area?
R2: Household done. But the workplace, as I told my patient used to live there. At that time, she took medicine for 4 months then there was a gap in her compliance. Now she has started again. But now she is at her maternal place. Her contacts are here also. It is somewhere left.
R3: It is the same here.
R4: Household are traced but some migratory population is there their follow-up is taken rightly.
I: Sir, how is it at your place?
R11: Households are done but there are difficulties in the migration population. Now extrapulmonary are more than pulmonary at my place. Pulmonary are not there now. But there are more than that at our place. (13:43 inaudible)
R10: Screening is done 100% but actually patients are diagnosed then diagnosis is done at our p[lace. Some come from private means from Taluka hospital. Then we start treatment then their post mortem is done. Like means we don't have a diagnosis etc. Actually modern techniques are there. Due to which we can detect on the spot. Means if done microscopic then its rate is flexible. The quality of sputum somewhere and the problem of transport. Sputum culture means it should be at Taluka level at least. Means such a big machine should be there at 2 places in Taluka where the test can be done last.
I: How is the situation at your place? How many contacts are traced at your place?
R5: Whatever are diagnosed from that they are migrated means somewhere. Half go to Mumbai. After that they came.
R6: means proper contact tracing is till 80-90%. I feel there is difficulty somewhere in Treatment, follow-up.

I: Is INH is given here for 6 months.
R: Yes, Rifampicin is not there with us.
I: It is not
R9: 3 doses. They should be done after 6 months. The second thing is plugin is not there with us everywhere. That is why tablets are there everywhere with us. Yes tablets are there. We give dose to children with tablets.
I: How much is the percentage in children?
R1: contacts or adult contacts. This is one and the second is 6.5 children.
I: Then how much contact tracing of them is done and their treatment good?
R1: It is done for them also. People in the house have more tension for children than adults, that it should not happen to them. Because of that it happens for children. We don't have syrup so we ask them to give crushed tablet. Till now I have not come across exactly less than 1 year or 2 years like that. But mostly 100% done for children.
I: Okay, how is rest?
R3: Yes
I: HIV-TB co-infection contacts
R1: Madam how it is mostly HIV-TB coinfection get treatment plus TPT with ART at their ART centers only. That is why we mostly don't have contact with them. They are not even in records. Not there they should give like that.
I: Are there contacts with other comorbidities like diabetes, hypertension?
R1: Actually frankly speaking we did not ask contacts till now. At least I did not ask if they have diabetes or hypertension even if they are adults, I will little try to ask from next time.
R: Actually the diabetes patient has expired.
I: We are talking about contacts.
R: Have not done for contacts.
I: Are there comorbidities in contacts? Or is their investigation done? Be it BP, Hypertension or diabetes or anything else. They have Hepatitis Disease, something like that or it happens?
R: At PHC means whatever means in contact of the patient they might be limited.
R11: There are not much contacts of Diabetes, hypertension. Means you are saying high risk.
R6: If they are along with that then they come in the NCD program.
I: Means is it done is my question. How is the drug availability?
R2: There is only one treatment of INH. It's availability, it's stock. After contact tracing it is available everywhere.
R: Yes central through yes. Yes, regular supply.
R1: Sometimes they come 100mg (instead of) 300 mg, then people have to give 3-3. That is why compliance is reduced a little bit. It should be available in both strengths.
I: How much follow-up and monitoring is done
R10: Follow-up is done. But symptoms. There is a different team at PHC for that.
I: Do they conduct house to house
R: Yes

I: Is there manpower for that everywhere madam?

R2: Yes manpower is there

R: They do up and down. Yes madam sometimes manpower is less

I: Is there manpower everywhere? No scarcity anywhere?

R11: ASHA workers are there and they work based on incentive. They do

R3: They have incentive. Means incentive based follow-up and it is incentive based.

I: Now we will come to challenges.

R: Yes

I: You are saying the staff is adequate.

R2: Means it is, but it's not like that. It is not adequate for what others said. Means no risk for ASHA workers. The staff is less for diagnostics, rest for follow-up or for their mobilization, to pick them up, this happens. Because it's not for X-ray. Because X-rays are not available. We have to call for a van for X-rays.

R3: There is a van, but it happens that the contract has ended or it has been given to private or its contract has also ended. Then if we go to RH, then the machine is not working.

R8: This diagnostic center has a proper base problem. This is the only one national program and I feel that diagnostic centers should be made. So that our (20:34 inaudible) we should get categorical confirmed cases. Then it is very less achieved. Our functioning is necessary. Then it should happen more. Then the X-ray is done. We do X-ray. Means even if it is abnormal at last it will come in CBNAAT. Till it is not done, proper diagnosis won't happen for our patient. It is there. Means we can achieve our target goal. It is 30, it is 25. Microscopy is also very less. Then their routine routine. Whatever their people are there and they are proper at our palace. Going too far (21:00 to 21:35 unclear)

I: How much is given to private from you?

R: We give it all to private.

I: All at private for testing?

R3: Not for testing. We do X-ray. RHTC, RCHC, PHC, SPH, RH are nearby for X-Ray. RH is there. We all have shifts there.

R8: All this is with us. In that we do one more private MOU for X-ray and they give X-ray to us. While doing X-ray, we do X-ray for those who have symptoms, our 4 symptoms. And how many done, how many abnormal. Even if we take abnormal, then after doing CBNAAT such X-ray (22:22 to 22:34 unclear)

R9: We do case finding. And then we do a sputum positive X-ray for suspected from them. We do X-ray wherever symptoms are there. And if they feel something, then we go to a private doctor. After that treatment is also told. In that we do CBNAAT. Only one minute.

R8: In that, sputum microscopy shows how much X-ray at us, sputum done for how many people, then how many people were negative after that, then it is important what advice was given to that patient. Means you should not leave that patient. It is necessary to do diagnosis of him. After that some advice from a physician, culture. Means it is necessary that you should at least do a culture of diagnosis. Madam means that patient is left in a half way madam. X-ray is done, money is spent on him. Means that patients don't go to proper treatment. That patient should be there. As many medical officers are there, it is the outcome of that patient. Even if it's abnormal for that patient, they leave it there only. Something should be done with that patient.

R3: Afterwards madam, diagnosed or suppose whose POD is done or more lungs pathology or other diseases are there. If their diagnosis is done, then patient cough, but or if we get tip then we

will give an online consultation, but an expert is there. But patients do not go there is the problem. For that if we get a facility like online consultation or something then we can carry that there itself. Now we have online consultation but MBSS are sitting there. So for expert advice like a chest physician, then it will be better for us. So we can do it on the phone or send an X-ray or other things. Their investigator if we get like that then that patient also there itself if we tell Sassoon for physician.
R8: Yes. like that. This abnormal situation.
R10: If a patient comes here then, even if the X-ray is abnormal then when treatment will start, till when to give and only one machine is there and when it will start is not important (25:12-25:15 unclear).
R3: And the patient's transportation problem is a little bit.
I: How is the functioning of X-ray?
R8: It’s toolkit is not there. Cartridge not available. It was not for at least 3-4 months, 4-5 months. One thing is, it was serum and what happens. It is done 100%, but sometimes it evaporates.
I: What
R8: It evaporates.
R9: We send moderative sample 100%. It is for pulmonary, rest nothing. Much scale and the second thing is madam, if it is sent, then patient has to do (25:34-26:00 unclear)
R: We are giving TPT to pulmonary right
I: We will talk regarding that
R: Because extrapulmonary is not diagnosed.
R2: At our place it is diagnosed because of a big hospital Sassoon or a big private hospital. Then that doctor tells to go to a nearby hospital and do it from there. Then we give. There are (patients of) abdominal TB, Spine TB also. Volumes of TB, quats are not diagnosed. At our place the diagnosis is done at the tertiary care centre. They come to take medicine at us.
I: Whatever you are talking about is regarding TB case confirmation. If contacts are coming then is it confirmed or not if contacts have active TB or not. Is it done?
R9: Means we check sputum. Means once the patient is positive then sputum and X-ray both, if they are, if not then no.
I: What about diagnosis?
R: 200 were taken from sputum. Some are there who do TB work. Means all do for TB. But frequent work, all are reluctant.
I: Are there any contacts of drug resistance?
R1: No contacts, patients are there.
I: How the treatment was given to contacts of patients
R3: Mostly what it was only husband and wife living in the village, means not the whole family.
R2: At my place a single lady is there, she does not have children.
I: Then in your case of husband and wife, do they have drug resistance?
R3: No no. Only one. They have different room in the farm and family here in the village, isolated like this.
R6: Most probably they are isolated. Because it happened to people, cough for so many days, fever. Social stigma for one of the patient. They themselves go to the side or people push them to the side. Contacts from their home should not be known. Suppose if some lady comes to her maternal place and it happens to her?
I: What about a pregnant lady?
R1: Resistant to which drug or if INH sensitive then we can give INH positive to them.

I: What more?
R1: No ma'am. Recently only one case came at my place which was Rifampicin resistant. It is not also otherwise for CBNAAT. I don't have it at my place.
I: Are there any cases of drug resistance at other places?
R: Normal cases are there.
I: They might be normal but are they pregnant etc? Are they Rifampicin resistant or multidrug resistant?
R6: Multidrug resistance are there
I: Okay. Then? What about their contacts?
R6: Contact tracing not happened
I: Rest what at your place? Is there anything else?
R: Rifampicin resistant was found. We do surveillance to check if it is rifampicin resistant or not
R: Patient cases are there in each house. Diagnosis is done in the city. But generally in each house.
R11: At my place drug resistance has happened there. Treatment has taken for 4 months.
R10: It happens that theories are closed or come after being diagnosed at INH Center.
R: Recently no drug resistance as such.
R: It might not be now.
I: Even if it is not drug resistant and now you started treatment for contacts. TPT. Then what is the compliance and in what proportion is treatment outcome. We are talking about contacts. In what proportion in contacts, how much percentage means it is real;ly started?
R1: Mostly 100% start. But I don't have a follow-up of who takes till 6 months. Now I will try to take 100% from ASHA. Till now nobody told me madam that I took for the whole 6 months or left in between. I don't have feedback from ASHA or I myself did not take follow-up with anyone.
R10: So I ask ASHA whether they went to the house. Second time takes that. The patient says yes. Did now. We will talk to ASHA in this month.
R8: We ask photo like you went, ask for photo after taking weight. Do not show face and send us the photo.
R: We give medicines for month wise
I: Yes. Do they come
R4: No, we give through ASHA. Give for one one month. We take weight etc after that. If we give card like TB patient's follow up for TPT then it will be easy for us. It will be easy for us to ask the supervisor.
R: Is there a timeline with us?
I: We will talk about this. Rest, what are your opinions means treatment starts in how many contacts and how is their compliance? And is their outcome, treatment outcome is investigated? You must be doing something?
R11: We go to ASHA only for contacts. Patients come for 1st visit and after HP has started for them, then monthly follow-up comes from ASHA only. Because patients don't come. They say nothing happened to us, then why should we come?
I: Then do they come for compliance or not?
R11: They come. ASHA does follow-up, then they come. When contacts are there for a visit, TPT comes and they leave. We take one Aadhar Card. There is more stress on MPW than ASHA. Efficiency is 90%. That something happens. If it is 100% even our local. TB happened to us or not. There is one benefit if MPOW is that if treatment is happening and is it 100%.
R2: TB patients himself come after medicines are finished or if they have some trouble. Like that even one of TPT's did not come till now. We are saying compliance and end of treatment, follow-

up does not happen. If cards are given like TB patient's cards. If given for TPT, then we can also ask. Means we can also remember that it is there, that's why it should be looked after.
R6: Actually it is normalized. TB happened. They feel that okay I have TB.
I: Do you want to talk more regarding this? We will talk about difficulties. While starting TPT, while implementing, as you said for compliance. It is not going if not looked after. What do you feel, what more difficulties are faced or can be faced?
R11: Suppose one active patient is there and he/she has expired. Then according to guidelines immediate contacts relative though we to them (35:29) though recent.
R3: After we call means expert say we are also MBBS, why are you asking us?
Us you us
I: What other difficulties faced?
R1: Mostly what happens, TB's stigma is still more in tribal area that index case themselves are scared to come to PHC. Plus if we tell them that ASHA will visit by coming to home, etc then also they are scared. Means it is more on the medical side. Then it happens that they themselves don't come then when will they bring contacts to us. So in that case most contacts are missed.
R: That index case is also not ready to tell. Because one such case happened at my place. That madam I will collect medicines by secretly coming to PHC in the afternoon time. But please don't send anyone to my house. I will myself collect medicines every month like that. Then contacts are far away. This happens. This is big. Still stigma is a lot. Index says no to come then contacts will be far away. Many times notification comes from private that heavytake is in private.
R2: Then if we do through ASHA that then they have to do entry through Nikshay, at that time also people do not come many times. This happens. No, we don't want to come to the government. If we go to the Government, then everyone will know. This happens. So, their contacts are also missed. I don't feel that they are giving preventive therapy in private. So, this is one of the big challenge. Stigma is one big challenge and means the patient is not ready to accept it themselves. Then how much their contacts. Then it might happen that I am getting a cough, I won;t check. Means it should not happen that I have TB, after that it will happen to my house/family members. Then I won't tell I have symptoms of TB. Negative impact like this can happen. I feel TPT awareness positive should be done before that.
I: How many patients' entries are done on the Nikshay portal which come from private?
R1: No, we do it by our capacity. And how is it, Nikshay has Aadhar Card and bank account details. It doesn't happen without that. So at my place 1-2 cases such happened two months ago. Notification came from a private and got to know that sputum positive are there in this lab. And what happened is that, there is a girl means unmarried girl. Her Mother father is like that No No we don't want to disclose and we don't want to give her anything. So that girl did not come to Nikshay till last. They did not give her Aadhar Card, did not give bank details.
I: Private treatment took?
R1: Private treatment was taken sir, but it did not appear on Nikshay. This was main and one more was like this. But we somehow convinced them. Mostly this happens more in case of unmarried females.
R8: Those who did not come, from that one member compulsory that private notification or TB's patient which is cured. Take, so that awareness will be created in people. This will happen like that. Whatever happened. Took rifampicin (39:15 to 39:21 Unclear). Completed of TB. Treatment is there means he so that awareness should be there in people. TB's patient came after completing treatment means cure and can sit amongst us and that girl was married. And she came and was

sitting here. It was earlier and they are coming here at some proportion. Sometimes what happens is that educated people come.
R2: Educated people come. Little bit. We find literate, much awareness. And we should not ignore. That patient comes to our place, special treatment because of that one patient miss.
R8: They say they have taken treatment. Means they don't shy away from being TB patient. I am cured.
R8: Treatment complete means such in 2 cases. This is my observation. ANM working in private was at my place. Means she was diagnosed there and came for only treatment to me. And afterwards she sent sputum from home only. Means we were taking during treatment but afterwards she did not take in Nikshay. She said no because she was unmarried. Because the saying of her house/family members was we don't want to do in the village. Being ANM means being working in private she took treatment from us. But afterwards did not contact.
R1: What happens that only one comes from a household in contacts. I am really telling only one comes. They will tell the names of four and then their weight then they will write. This happens. Because all are not ready to come. The one thing is at first they are scared because of the index. That is why the rest else don't come. They don't come in front, in short we ask for symptoms someone from the household means. In them we ask for whom we are starting. They don't have this trouble like cough, weight loss for the time being only. That patient is actually not in front. Means beneficiary is not front actually. This happens. It happens that only one person gives information of everyone.
I: Does the same happen at other places?
R5: Yes, the person patient takes treatment at private for one month. We did this at Private, send ASHA (42:14 to 42:38 unclear). Even if people say them that they have to take treatment for 6 months
I: Will you tell me the experience of your training? Training took place, right? First time where it took place, when?
R3: Did not happen for TPT.
R8: It happened for TB. Not for TPT.
R: It happened for RNTCP.
R8: Ours took place for RNTCP. We were together
R1: Official training did not take place sir. But at one time, came to PHC as an experience that we are starting this. And they gave chart etc in our OPD and that's it.
I: That information is very nice. I thought you give training to people.
R1: Sir I have worked at Male also at National Health Institute.
I: We met some time ago.
R1: I also feel the same sir. So at that time earlier we were giving to HIV contacts only. Know the guidelines from that time only. That is why it was a little bit easy.
I: Has someone's training happened?
R11: Not happened for TPT. I read a PDF at that time of TB.
I: Was the print of PDF given or what?
R: Instructions were given. One thing was we have to give.
R3: It was told in a meeting that we have to give. Sir also came.
I: Which MO was there from Walne earlier Darade.
R: Yes Darade sir told. At that time he was taking. It was something like a block meeting.
I: ITR has started. At that time this was not there. Actually we were seeing print. (44:25 to 44:30 unclear)

R8: At first Taluka MO, CHO, Lab technician also pharmacy doctors also and at first their screening took place.
R: Was it given deliberately because of less work.
They keep block wise meeting and their Baramati and in short something good (44:57 unclear)
I: Are there any more difficulties in TPT?
R10: What happens madam, when they go home from here, patients also sometimes speak more. Like they are doping favour. Then ASHA also gets disturbed. That we have to go daily, do that. I just want to say this.
R8: the one good thing is that injection had to be given for small things also. Injectable made less now. ASHA and Anganwadi. Drug to ASHA. It is a little bit necessary to give training to ASHA because they will also run into problems. Already they are at high risk. This is one characteristic.That is why it is necessary to take a little bit of care and awareness.
I: Whatever you started all this, the objective is for elimination. What will be the difference of TPT seen for elimination. Your frank opinion. How much will be by new strategies.
R3: It will be quite a lot sir.
I: You tell me about TPT's utility or elimination. Good or bad whatever it is.
R2: Spread will be restricted first after TPT is started.
R10: Means closer contact is there, suppose it happened from him then also means if he goes out, if he is working then there will be little bit benefit for spread. Like we say, prevention is better than cure.
R8: Now in COVID also, our means incidence rate will be reduced. Because of that. Means for incidence rate, will go to eradication. We will go there. But for that 100% screening is necessary. That is why follow-up is also important. It is important to train all. Awareness is important. Means the precaution should be such means we have worked on COVID. Like in vision to eliminate again in COVID vaccination done by Sagar. It happened. It might have gone, that is why this will also be better. Have done study for COVID. All have experience. And someone's is that we have come for treatment. It is not like that (we) don't want to take treatment.
R8: Awareness regarding respiratory diseases is increased after COVID. That is why this will be better. Means I feel it will be a success.
I: We have to do this in the allotted time only next year. What is your opinion regarding this?
R: have done COVID sir. One-two will be
I: What do you feel? Will it be done by 2025?
R2: It cannot be. It cannot be.
R3: It is right. According to WHO 2030. 30 is said then also awareness is increased as I said.
R: Quite a lot of guidelines have to be revised a little bit again. Awareness, guidelines, compliance. Overpopulated are there that is why Compact area. That is why I feel that elimination won't happen till 2025.
Crowded area have to be targeted a little bit. Means Mumbai.
R: Proper microscopy means tell a little bit about me.
R3: Yes for diagnosis again if the patient did this. If CBNAAT is run properly, Falcon tube's sample properly transportation and all this happened. Then more detection will be increased.
I: When do we get the results after we send. Just curiosity.
R: Sorry
I: if we send tube to RH, then when will we get result
R1: Actually it gets in 1 hour.
I: When do you get

R1: Sir, get reply mostly. Mainly, don't go early. The topic is this.
R: If the sample is run till next day that day only if it is positive then anyway they call immediately.
I: Is it not in 48 hours?
R: Sir if it is negative then don't get mostly. We only have to call what happened to sample.
I: Means there is no difficulty there.
R: No sir
R10: No, it happens sir. It does not take place early. Suppose the main morning sample has to be taken suppose. Suppose we send that. That patient doesn't give. I only send one person from here suppose. If they say no, today our machine has load. We are doing other PHC's this this. Then it won't run. Again have to go to take that sample. That one is there repeat difficulty.
R: It is very difficult to get everything together. We have one at our place. His posting is MBBS. And he says that this happens to most of the work at their place. Machinery. And their lab book which is there. Means that madam, and there that to the lab. (49:41 to 49:45 unclear)
R10: We have problem like if that madam goes to rural for three days then we have to sit for three days for a microscopy. And the second thing is the loan which we have done X-ray machine, it will come once in one Taluka.
R8: All activities in 2-4 days. Will do an X-ray. If 50 done one case, if an abnormal 20 came, all will be sent in 2-3 days only, right? Means sample and we also don't have a second option. Have to send it at that time only. Afterwards the patient is not found. People don't cheat.
I: What about PH?
R8: I feel now new techniques are there. Have to work a little bit in diagnostics little bit.
I: If a little bit of work is done on diagnosis, what do you feel what changes should be done in strategy so that we can achieve our goal of elimination?
R2: How is it in our coffee that once that patient comes positive then have to trace immediately. We have to send his name to the nodal officer. Like that it happened to that also.
I: Review?
R2: Yes, review right
R6: Now I am saying that till now it has become successful.
R8: But have to say that if we take operational data. That day Zoom meeting is there. Different meeting should be kept for that day. And main means the one for whom we are giving.
I: You asked a question that is there anything like this? We have kept the level for this Gold level, silver level, bronze level and if your agreement is best in these levels, then you have to claim. Your Taluka or district district has to claim that we do very well.
R: Ok
I: If we look then DTO and medical college together go to the houses and confirm whether these claims are true or false. And if it is then they give Gold points to you. In our Satara claimed Gold or Bronze but we cut marks at the time of inspection because it was little less.
R: Okay. As it is done as you said. And three districts were eliminated in Jammu Kashmir.
I: Ok
R: But this year Sangli district will come first.
R1: You keep quiet. There is a scheme like this. And this can happen and this can happen in reality, three are examples of that. Means I told. And whatever are there at least we have a drug supply main problem. Now there are no drugs from March month.
I: Not to everyone
R: Sir this is a setback to the elimination program.

I: You know, what do I feel? If the problem of medicines is due to bad luck, then it is a setback. But this thing is in our hands only.
R: Yes sir
R2: Having said that to eradicate
I: It should not have happened to us at this time
R3: They ask review to us. But if strongly review of specially MPW is taken, do you take follow-up or not? Field worker will give a lot of improvement on this thing. We ask in meetings how many patients are found? What happened? But if for their team more means don't want target oriented work then it will happen properly. Because they are not asked any questions anywhere. That is why it is just like this.
R11: They don't let do entry in the Central Government's Nikshay etc. That's not how it's supposed to be. It should be checked. This is important. Gold level means Kashmir got it.
I: Yes. To Kashmir. In three districts Gold and Bronze to Kashmir.
R: Can we apply their strategy here?
I: Is there no one from Satara Sangli? But have you gone to people from Sangli?
R: no sir
R11: Right now we Active TB patient finding again. Their contact tracing, we are doing whatever discussion on this, do mostly. The main difficulty that will come during elimination will be the migrated population, that will cause difficulty. Till we give them treatment for 6 months by keeping them in one place. If treatment is not given for 6 months to contacts. It is not given to them. Then it is very difficult to achieve this 100%. And whatever migration is there in India it is interstate, interdistrict means they will be main challenges for elimination.
I: Approximately how much percentage is in migration?
R11: 5-10% happens relaxedly. And Daund where sugar industries are more there people go here and there at 4 month. Or when Taluka to Taluka happens. Many such Taluka are there in Maharashtra for Sugar production, for construction, MIDC area. Challenges are there at such places. Even the process is a topic. Seven migratory population were found in one day at places like Bhukum. Those who were taking treatment, Newly diagnosed and people who left called defaulter or failure are found. The same condition can happen in the case of PHC. Those who left treatment halfway then it can happen again. This is one factor.
I: does anyone want to express their opinion? Challenges and what strategies can be applied?
R10: Awareness, because mostly to patient COVID related
R6: Those who have elimination information should be transferred siui meter.
R11: If the Government wants to run this program then the private sector has to be involved in this. In that, whatever technical staff of lab is there means it is technician or diagnosing person, more training should be given to them. We have to increase technical manpower. Here the condition of PHC is such that they don't interpret, they don't do anything. Because of that it is known later on that positive patient is positive. That is why there is a difficulty even if have to refer. It is difficult till we don't increase technical manpower.
I: How many PHC are there at your place. PHC or Pune district, it was told that we will give 3 Crores every year. Now I don't remember. They take on motorcycle or in the Pune district.
R: It is not
I: Their counseling is also done. Is there anything in Pune district for counseling also? Have you seen? What Government has started?

R: One NGO told the patient which are in private in Pune district. Their sputum checking, their entry on Nikshay, to see their HIV status, to see their diabetes status, see regulatory. Called from NARI for active case finding.
I: It is different for NARI. This is an NGO, a contract is given to the NGO. That varman or something, but now I don't remember the name. Then it is expected from them to do this work. Then we will take their number telling DTD afterwards.
R11: What happened was that, the posting was given at places where samples don't run to collect sputum. His work was to collect sputum from PHC.
I: Correct, and to transport/return again.
R11: Then what happened, he brought but his payment was not issued so he left.
I: Okay, we will see. It's intricacies are not known.
R11: This topic is also important. That if we appoint such and their payment is not done.
I: ASHA tell appointment means you transported a sample this much money.
R11: We used to give lot of money for one transport, one sample
I: yes like that. His interest is gone. We should leave.
R11: We left that topic.
I:: Does anyone want to present any point?
R9: Now we spoke about prevention and elimination. Then they are said ASHA, Anganwadi, ANM their union say that, health officer. Because if we say only ANM, then their work is only till introducing. Even in our program we say ASHA, Health officer/servant, and ANM. What happens after we say this, name happens, In that Health servant should be said instead of ANM. this is there. In that we only did screening. A little bit in the paramedical program. They should come. That is why demand is from them. Do this, do that.
I: What more?
R6: Till now timing, staff etc all training. What are high guidelines? It should be read. State guidelines, Central guidelines
R1: M'am just to share one experience. I have worked at PICU also, sir. There is one program Focardi running.
I: Yes
R11: In that Kinikar ma'am etc. They had done one study. In this was there that we our residents and nursing students, those who are newly joined, BSc nursing. So according to them, whether there is a latent infection or not and over a period of 1 month it was developed in how many patients and those who have developed we were offering TPT to them. So their opinion was that even our medical students means residents also, this much means we say that they were resistant that formal identity should not be there and those who were positive in that they latent TB infection newly diagnosed. Two of them suffered from TB but then also if this much resistance is there in medical people, then it is a big challenge that how much can we do in general population.
I: Thank you

THE END
