# Supplementary material for: Factors influencing tuberculosis preventive treatment implementation in India: A hybrid inductive-abductive thematic analysis using implementation framework
Source: PLoS One. 2026 Oct 14;21(10):e0359171. doi: 10.1371/journal.pone.0359171 (PMC13667825; doi:10.1371/journal.pone.0359171)
Supplement: Supplementary file 4 [file pone.0359171.s001.zip › Focus Group Discussions (Adult contacts).zip!/Focus Group Discussions (MO)/FGD_MO_2.pdf]

FGD_MO_2
Date: 30.04.2024

I: Have you heard about Tuberculosis Preventive treatment Guidelines? Do you want to tell anything? Do you have any information?

R7: We already give Pulmonary Tuberculosis Treatment to Sputum positive patient after the RNTCP extrapulmonary are also there.

R3: It depends on sputum examination and x-ray.

I: So this is case detection and about that, right? Have you heard anything about TB Preventive. You tell whatever you have heard.
R3: The one program about adult BCG is launching
I: Okay
R3: In that, adult vaccination is done. BCG adult vaccination is done. All is going on.
I: Training done.
R3: It started
I: Have you heard anything else? Has your any training done regarding this? Do you have any information? Give whatever information you have?
R1: Preventive 2HRC etc. (we) Give Isoniazid, Rifampicin. We take confirmation according to CBNAAT, X-ray.
I: What are you talking about? Case related?
R1: Case related
I: Should we talk one-on-one because we won't understand audio recording.
R2: TB's contacts are there. There is one contact register with us. Then we give tablets to them according to that register. For every 2 weeks like give tablets for 6 months. Isoniazid form register
I: How do you make it?
R2: There is a separate register at PHC.
I: No. Means how there is a list of these people in the register. How do these names come?
R2: We take all these names through ASHA. ASHA does a survey by going there. Who comes in their contact. At the start they go home. We do testing of those who have symptoms from them. We start TB preventive Therapy for those who are negative and start TB treatment for those who are positive.
I: ARe they household contacts?
R2: Yes, they are household contacts.
I: Sir, do you want to talk about anything regarding this?
R3: Some contacts are from household only. We give preventive Isoniazid tablet to positive patient also in the family. If the confirmed sputum positive case is there. Earlier we had that give till 5 years only. But now it is removed. Generally we give INH tablet to those who are all in contact
I: Okay. Sir, you were saying something some time back.
R: Yes, regarding preventive.
R7: You said that avoid overcrowding area, using a personal protective mask. Now the new launched BCG vaccination for all those who are above 18 years. Preventive measures these.
I: Sir, what do you want to say regarding this?
R: No.

I: Okay, was there any training conducted regarding this for you?
R7: Yes, were conducted for TB for us, conducted for RNTCP.
R3: Adult BCG means that was the only point in that.
I: Okay. Have you read any guidelines regarding TB Preventive some from Government, some books, some guidelines like that. Okay now you told some points like contact tracing. Will you please elaborate it, that which things while implementing stepwise. Will you be able to tell.
R4: Madam TB case is detected. After detection in them whichever age group are there, different different age groups then their group and according to that different different doses are there. We use masks like sir told or to prevent, isolate outside like. And in our system we ask ASHA workers, MPW our down staff to visit from time to time. And if symptoms are increased or screening is completed we aware that you take images or don't be scared, complete the treatment.
I: You put a point of screen. Then how is it screened? Which test is used?
R3: We do a sputum test live if it is softbik.
R4: Otherwise X-ray happen everywhere.
I: We are talking about contacts, not cases. Does sputum of contacts done everywhere or X-ray of every contact?
R3: No. We give treatment contact systemless. We start treatment even i there are no symptoms.
I: Then, is X-ray done for everyone?
R3: It is not done. It is done for those who have symptoms.
I: Sputum is done for whom?
R: if it is negative and if they have a problem, if they have any symptoms we do an X-ray.
I: Is sputum done for everyone?
R3: no. Not for everyone. It is done for those who have symptoms. But treatment is given to everyone.
I: Okay. Are there any contraindications regarding this? But other than that are there any like if these things are there then this person is eligible or is not eligible. Or there are some contraindications. Are there things like this?
R: Eligible means not only symptoms in that. Some are diabetic chronic diseases, some kidney disease susceptible or malnourished children, weight to weight loss or having some comorbidity or HIV positive patient. These are more susceptible for TB. Such some.
I: What is done to such people then? Is screening done or X-ray is done and after that?
R3: Treatment is continued. we start prophylactic
I: Okay. What is the compliance of this? Is anything investigated? Are they taking? Do you give drugs every month? How do you give three months collectively?
R3: They are monthly. Through ASHA worker.
I: Then when do they come to PHC or not?
R: Patient comes
I: Contacts?
R4: If there are symptoms, then only. Otherwise not.
I: Okay then drugs are given through ASHA through only. Okay then does any information is taken if they take or not?
R3: Staff looks there. Means it can happen by visit. (They) see means after completion then empty packet is found. After that the next packet is given.
I: Does this happen everywhere?
R: Yes

I: Or there can be difference also. That some different things are happening. Whatever it is, you talk freely. Don't feel like. Is there any discussion regarding counseling?
R7: Means we do counseling before starting the treatment. How to take, is there any trouble? Because what happens after, some people cannot digest tablet, they vomit, etc.
I: Are there any side effects because of tablets?
R3: All this is done to confirm the case for starting counseling. Means what precautions should be taken.
I: Sir, do you want to say anything regarding this? Sir, behind; do you want to say anything regarding this? Do you want to share any experience regarding counseling?
R9: This after diagnosis mostly. Are they taking medicines properly?
I: You are talking about whose counseling?
R: Cases, confirm case.
I: Is anything done for contacts?
R: TPT is provided to contacts.
I: Okay. Is any information provided regarding them counseling? These things happen. Do you want to say anything? For those who are not ready to take TPT (12:13 to 12:20 Unclear). What can be the proportion?
R9: They are not ready at the starting.
I: Approximately means not absolute numbers, tell approximately how many don't get ready out of 10? How many get ready?
R9: 50%
I: What are the experiences of rest regarding this?
R4: Most of the get ready but not that much. They start. 20% are not ready (4-5). Otherwise rest of the people take.
I: How is it at your place?
R3: How is it at our place that patients come as scared after they have TB. There is a fear. That is why we do a little bit of counseling of contact who come with them. We do a little bit like take precaution, little bit like use a mask, they get ready.
I: Okay. Availability of drugs, what do you want to say regarding that?
R7: There is a shortage now. But no.
I: Is there any problem in case of medicines? Shortage is from how many months?
R2: It has been two months.
R: Sometimes not. now list (13:33 to 13:40 unclear).
I: Has everything stopped regarding that or at your level?
R4: Some specific cases, madam, some specific PHC have problem.
I: Okay
R3: It is in lister track only
I: How is it at other places?
R2: It is not at our place
I: Okay. We drew a conclusion that it is at some places and not at some places. Now you gave medicines. What do you want to say regarding monitoring? Regarding contacts, now you gave medicines then their monitoring. Do you want to say anything regarding this? Do you want to share any experiences? Do you want to tell any information?
R: Is it close contacts?
I: Yes, close contacts
R2: If they have symptoms then their testing is done after six months.

I: then is the follow up taken for six months?
R3: Follow-up should be taken. It does not take place that much. It is through ASHA workers only means if it is three, then only tablets are given. Regarding staff, we have less staff. Here no sister for PD, then when will go to places. It happens that is why their visits are less.
R4: Generally STS of TB program visit and our MPW visit and it is not like that contact will meet on that particular day or he is at home at that time. Then if they have any symptoms, they come to PHC.
I: Okay.
R4: And there are not that many cases. 10-12 each cases are there. Their contacts are majorly known, because it is written on the treatment form we give. How many children are there, how many adults are there in their home contact. Their age conclusion is there. Based on this update is taken in a meeting. In that full discussion is there. What are their treatment stages, monitor one of the patient or we also tell that visit once. Everyone will get practice. We tell ASHA also.
I: When did this preventive treatment started?
R3: Approximately many years took place. Only there was an age limit. We used to give till 5 years earlier. Earlier we used to give to little children. Many years have passed.
I: From when you used to give it to adults?
R3: 5-6 years have passed.
I: Were there any adverse events? Has anything found under anyone's PHC? Or any drug reaction took place or do you want to say anything regarding what to do in such a case?
R7: No. No adverse reaction. Only minor effects.
R3: Sometimes the tablet is not digested, they have vomiting, acidity. They say every time that they have vomiting, after eating. then we say that we take after food etc.
I: Okay.
R3: From our place multivitamin syrup
R7: or anti or antacids etc are given
I: Do you want to share any experience regarding this? There is a contact means case contact and treatment started for them or not started. Whatever it may be. Can you share an experience like this? ANy experience at your place like this regarding this. Any different case, do you remember that. Would you like to share such an experience? Any pregnant women came at your place that case contact and pregnant women like this or any other condition in contact. One of the women can be pregnant right?
R: Yes
I: Okay you say there are registers of these as you say. Then can you say how they are maintained? are there registers everywhere different different
R3: Our patient form is there. We use a register or form.
R4: There is a form. There is a case sheet. It has two copies. One goes to the health center and the other goes to a place where it is. We give one box of medicine collectively or give as they come 1 monthly.
I: And are there forms for contact also?
R3: No. No not for contacts.
I: we are talking about contacts.
R4: there is only numbering. How many contacts are there, what is the age. It is written on a simple register. It is written in sisters notebook that we told this person on this this date like that.
R3: There is nothing like special. There is no register as such with ASHA. They have a notebook or they write in a regular book. There is nothing different as such.

I: Is there any participation from the private sector? What is your vote? What is your experience?
R: Skoda company provides food. Nutritious food. Nutrition food. In food packets. And peanuts, oil, that oil.
I: Then is it to specific TB patient
R: Yes
I: And is it given to their contact also?
R: It is not given to contacts. To cases
I: Is a food packet given to the patient or family?
R: To patient
I: Does patient use THe food packet which is obtained for himself or they share
R: Did not ask such question
I: Okay. Your such experience. You might not have asked but have you heard something
R2: they must be sharing
I: Skoda company gives at your place? At other places?
R: No at whole Taluka place only
I: Whole Taluka?
R5: It is in Shirur also
R3: And now our all for three department patients means sponsor means use food packets through our department. There is a family of a TB patient for the last 5 years. It is given to all.
I: Is it seen what happened to these people? It is given for contact. You are saying you give for every month, then you give month month as such. What next? Have they taken tablets or not? Or is it investigated whether it is changed or not? Is anything investigated? Afterwards if said for contacts, a patient comes every month.
I: I will ask different question. Suppose everyone has 10 patients. Everyone’s 5-5 contacts, they experience some 50 contacts. Is it? Is TB really developed to anyone? Tell experience of perfect with contact is not happened
R4: Everyone’s common experience is huge. After 5 years 10 people’s 50 years experience. We are giving to contact so that it doesn't happen. But theoretically it will not happen that it is 100% prevented. 1-2 can be out in that.
I: It can happen. But what are experiences? You just share your experience. Did you find something like TB found after going also.
R3: It is not found in contact. Not at all found.
I: Do you feel challenges can occur in this. Regarding contacts like Screening them, giving treatment, follow-up, compliance, what difficulties can happen? What problems can be faced?
R4: Difficulties. If we give medicine to them, their resistance is 20% or 25%. Anything don’t happen to us.
R7: That’s it. Only if there is no guarantee that they will take. they will say for full treatment at start and they will even go for an investigation is not like that.
R3 Nothing happened to us. Eat tablets, if we send for X-Ray at a higher place, they won’t used to go.
I: What do you think? What difficulties can be in this?
R7: They will take tablets or not. I am doubtful if they take or not. Because what it is there is a stigma if we say stigma. When there is a TB to self, at that time also nearby it is known that he has TB and to pursue close contact. And what happens suppose he doesn't have TB i.e., close contact and if he is taking treatment then without reason it happens that people don’t know that. Close contact is a word between us. If someone is taking treatment, then others feel that he also has TB.

He comes and go in hospital, it is okay if there is privacy. But some things cannot remain private. One of the things is out. We say close contact, he doesn't have TB, But if he is taking treatment, then misunderstanding happens. Because of that resistance, if he will take or not next or much problem is on that. This is one of the reason for not taking it.
I: What more can be there?
R3: Social stigma can be there, that Baba what will happen, this is known cause.
R7: Nothing for those who are confirmed, but close contact means to pursue him, to give treatment is very difficult
I: What do you think what more difficulties can be there? Okay. Government of India has said that we have to eliminate TB till 2025, then what is your vote regarding this? How much it can get eliminated in one year? You people are working on field.
R6: It is challenging.
R7: The special programs were also implemented in between.
R3: Survey of 1 month happens. Many cases were found from that. And now adult BCG will help then that have consent, that is why the big question is how many people will be ready. It is to be given to 20% people but they won't be ready at our place easily to give BCG at this early.
I: What more difficulties do you feel that they should be negotiated and how? It might not be 25 but it can be 30 or 35, can TB get eliminated by that time.
R3: Counseling and survey. If survey is proper and house to house and if sister i strained then no. of cases can get detected early.
I: Okay, what do you feel sir?
R1: Counseling is necessary. But the BCG vaccination is in less number of people's mind that what can be side effects and not.
I: What do you feel sir more, what should be added?
R2: just like how we did COVID vaccination widespread like that IEC etc, it will happen for this also. To get it eliminated through vaccination, many times pulmonary is not seen. Directly extra pulmonary is seen. Primary pulmonary is done/ has happened. Pulmonary is done. Goes to extra pulmonary.
I: It will get eliminated through vaccination. What do you feel more?
R6: Continuous monitoring is necessary.
I: How it can be done? Their follow-up, how much done. Sir what do you feel
R8: Madam survey and monitoring is necessary.
I: Sir, what do you feel? What difficulties are there to eliminate TB?
R9: True case finding survey.
I: Sir, what do you want? Irrespective of the points which are told.
R4: In awareness also for rest infective disease or infectious disease prevention the as much as IEC or as much as awareness or as much as stigma is there it is necessary to reduce that. What will happen because of that then we say today that this week we ask patient that he will tell how much it is. If we say by giving a little bit of stress he says the truth. It is from two days only. This is the truth. This is nothing. If it is a fever, they are listening, watching on TV. They know. Fever comes at evening, don't come. We know the patient is lying, it is necessary to screen 100%. But now also there is no acceptance that I have TB or I can get TB.
I: Sir, do you want to add anything in this
R7: I feel there will always be hidden cases. It is not guaranteed that 100% people, all people will come to the hospital or Primary center hospital because ASHA will go everywhere. Now some slum area are there. Some will spread like this by coughing. We don't even know. At these places

some hidden case percentage we cannot tell. but 2-3% will always be there. That is why we say that all our efforts are continuing till 2025. BCG vaccination, timely detection, treatment, etc. But I feel some % people will always be left out.
I: Sir, do you want to add anything to this?
R5: They should come for follow-up. Sometimes what happens is that we say tablets to them, but they don't come for follow-up. And don't know if they take most of the cases.
I: Do you think TPT will be useful for TB elimination? For this whatever Preventive treatment for contacts is there, then any use or any changes in this, anything needs to be added, this is too much told do you feel like this?
R1: Proper treatment should be taken for preventive.
R3: no treatment is right, but taking proper treatment for 6 months is necessary. It is necessary to monitor that. Concentrate close contact. To concentrate on contacts.
I: Does all contacts come? At least on paper in list.
R3: Yes, they come in list. and take live. If they will take or not is next thing but they take medicines per month.
I: What is the percentage per month
R3: Normally they take. 5-6 contacts are there per case.
I: Yes, then there will be visit to them by going to home. And do they collect medicines per month?
R3: Yes they go there.
I: Does ASHA go there or contacts
R3: ASHA goes there. No contacts. Our staff gives. We distribute at the sub center, they take from there. Means ASHA distributes at house. Yes if it is at house or if sub center is nearby then these people take it by coming there. It is a must to go at home for close contact. ASHA or NM has to go. Everyone doesn't come to PHC.
I: What do you feel about how much % comes?
R7: The one-two comes with patient, that's it.
I: Do you want to share anything more regarding this Tuberculosis program related. Any experience at your place?Some case or any experience like that would you like to share? Is anything memorable whether related to case or contact? Any case any such experience would you like to share? Exclusive of this how many positive patients will you have approximately positive. Suppose how many positive HIV will be there approximately in PHC. Hardly 1% means approximately 30-40 in whole PHC or even less than that.
R2: Sir we make that testing compulsory in ANC. Those who want to do in ANC.
I: How many are registered in one PHC approximately? If it is 30 then you say how many will be there?
R7: Not more than 4-5.
I: Exactly what? Approximately 4-5 okay. From that how many for treatment?
R3: From that ICTC Treatment. Actually whenever TB patient come in TB halter HIV testing is compulsory. If they are detected then they are confirmed for ICTC. Confirmed for ICTC. Testing is done at PHC also.
I: Alternate of that, it is okay that your patient is tested for HIV. But what are your experience for HIV patient for contact or giving them preventive treatment. Whatever 4-5 patient are there in their house. TB happened in HIV positive patient's house. There Will be symptoms.
R3: We give if there are symptoms. No prophylactic as such.
I: 4-5 are less. And there is a cross referral TB and diabetes registration.
R3: Yes

I: Is it done 100%
R3: Diabetes is completed in NCD.
I: But is it checked for contacts that if they have diabetes or hypertension investigation like this; is done for contacts?
R3: Not for contacts
I: If they 30+, is it mandatory or voluntary
R: For mandatory. It is done otherwise randomly
I: Is it done everywhere?
R2: It is done
I: And if it is positive and Random Blood Sugar found 140, 150 then what is done next
R: If it is above 150 then they are called for fasting
I: Do they come for fasting
R: Yes, it is at every Subcenter because of CHO
I: How is it under CHO
R3: That is why ASHA are trained in doing sugar (testing)
I: Do they wait for this much time
R3: yes they wait. They have tension from all this. BP Apparatus etc is given to ASHAs. They do during visit at any place
I: Okay is there enough manpower at your place to do this?
R3: Not at my place. My state is such that 6 sisters are there permanent. There are 5 vacancy are there. Madam only 1 sister is working and 2 from NHM. 3 sisters are from Waghi PHC.
I: Is any training done for this staff regarding TPT? To trace contact, how to do, which information should be given, what has happened for ASHA, sister under you? It is not done for you but under them not done for anyone? You have got instructions. That’s why these guidelines came regarding that. We give then how to give? What to give? How do you see?
R: The dose has already come.
I: Then, is line listing done at the end for contacts. Means approximately, one number is drawn, that how many can be there? Have you traced them? ARe these things done?
R2: 4-5, all including Mother father.
I: DHO must be doing MO’s meeting review, right?
R2: Yes
I: Then, this might be one of the point in DHO’s (meeting). Okay, preventive treatment TB positive will definitely be there but is it there for preventive treatment?
R2: Yes it is there, in every meeting. District
I: Is everyone’s experience same? If there is a meeting at Taluka (36:07 to 36:11 unclear). Sir, will you be able to tell how many resistant tuberculosis were found among you?
R: 2
I: Approximately 2 MDR from how many? Rest about you?
R: MDR are 2-3
I: 2-3%?
R: 2 cases are there. 2 cases are there.
R3: They are not at my place
R: They are ready at my place
I: What is done for their contacts?
R2: Same
I: Same means?

R2: Same treatment madam.
I: Is the same process CBNAAT done?
R2: Everywhere at taluka. Yes means it is available
I: All this cartridge etc?
R: Yes at RH. Means no problem as such. Our Jagtap is there. Jagtap is our STLS right?
R: Yes
I: Who carries sample there?
R: Patient only goes there
I: Patient’s X-ray etc.?
I: X-ray also takes place and that also. No deficiency of X-ray. They do that.
It is online only right?

THE END
