# Supplementary material for: Factors influencing tuberculosis preventive treatment implementation in India: A hybrid inductive-abductive thematic analysis using implementation framework
Source: PLoS One. 2026 Oct 14;21(10):e0359171. doi: 10.1371/journal.pone.0359171 (PMC13667825; doi:10.1371/journal.pone.0359171)
Supplement: Supplementary file 4 [file pone.0359171.s001.zip › Focus Group Discussions (Adult contacts).zip!/Focus Group Discussions (MO)/FGD_MO_3.pdf]

**FGD_MO_3**
**Date: 15.05.2024**

A program was started means for contact of Preventive Treatment Patient.
I: Have you heard about this?
R3: Regular program started now, but we used to give before that also.
Now it is started on completion basis. Before that we used to give as per the drug availability or whatever it is. I just remember that we used to give before that also.
I: What would others like to say?
R: Used to give to the pediatric age group. The house which he belongs to, to that age group, is preventive. Means now we have started INH. From 2 years, and before that it was according to Cat 1 and Cat 2.
I: It was for patients. Now we are talking about contacts.
R: Okay. Now we have started INH, according to weight and according to age.
I: You are talking about whom? Case related or contacts related?
R: No, related to contact
I: Is any training done of you related to this?
R3: Training is not done madam. It was told in starting TB trainings which were done. For preventive treatment by weight, you can calculate accordingly and give dose to patients till 6 months.
I: Sir, is it done at your place?
R: It is not done. Doses are given. Seniors only do CBNAAT, TrueNAAT.
R: For Pediatrics, according to weight age and for adults 300 mg per daily dose, like that given to contacts.
I: Okay. for contacts
I: Madam, at your place?
R4: Same
I: Here sir, is any training done related to this?
R: I have heard
I: When did you hear?
R5: This tuberculin skin test, IGRA and to do that 115 countries.
I: Okay
R: This much only
I: You?
R6: I have only read in textbook.
I: I s such training done after joining here as medical officer
R: Till now no
I: Okay. Sir, what about you?
R12: Training is not done. Booklets. Majority charts, etc came
I: Charts related to what?
R12: We do our IEC. In that majority of charts at our place where there is PHC, reading there and whatever updates come, according to that. It’s training is there but till now ours is not done. TB’s one training is there, it is not done yet.
I: Is training done by anyone among you?

R8: No
I: Okay. What are its points? Can you tell, what all things or points are told in our TPT? TB Preventive Treatment, especially for adult contacts, what things should be done? Your formal training is not done as you say but whatever done in RNTCP at the starting or as he told IEC charts came, some information obtained from that. Then what all things are there in this? What needs to be involved, needs to be seen or something like this? Madam, can you tell something?
R11: Madam regularly their screening means is there any history of cough, do they have cough. Do they have something else like other illnesses like diabetes? There is a necessity to do regular screening of all these things. And instead of cough till 14 days, examination should be done if there is diabetes and other complications.
I: Sir, what can you say?
R2: Are there any patients at high risk.
I: What should be seen in high risk? Can you tell in detail?
R2: The diseases you said cancer etc, diabetes, smokers etc are illnesses. (4:15 to 4:24 inaudible)
I: Does anyone want to talk? Is there any more sequence? Isn't it so. Feel free to discuss.
R4: Mostly after screening first before starting HIV ART. Suppose if it is positive. Each other's experience. After that ART like this
R4: Your question is that what all should be done before starting for contacts
I: Yes
R4: Basic blood investigation should be done. Because we give INH till 6 months. (This is) liver toxic drug. So basic investigations should be done LMP, IMP, CBC. So that after making sure their liver function is normal then only we should go ahead.
I: Then, does this happen?
R: No
I: First point you said screening and liver function test and other blood test should be done. Is this happening or not main?
R5: Symptomatic screening is done.
I: Okay.
R: Blood tests of contacts less
I: How is the situation in PHC at your place? Is the screening of contacts done first
R13: Yes, it is done
R6: Screening is done and we call them through ASHA. By coming to PHC if their cough is more, then by doing a sputum test.
I: Okay
R6: Whoever has come to take tablets, we tell them to keep far if there are children at home. Or if they are suffering more, then bring them here. Like have to create awareness.
I: how is it at your PHC? Is screening done?
R12: Yes screening is done, blood tests are not done.
I: Blood tests are not done
R8: Same, symptoms based are done madam blood test and not directly. If complaints like cough, cold as such if said clinically then like how we take normal TB patient
R10: If such symptoms are seen in someone from a household then X-ray for him and for the rest of the things and if sign symptoms are there, if sign symptoms are not there then investigation is not done.

I: then blood test is not done but is sputum in such cases?
R9: yes we do sputum.
I: Is it done at all places?
R9: Yes, Sputum is done madam, but there should be a 10% quality rate. This doesn't happen. Majority times quality sputum doesn't happen. Quality rate should be 10%. This doesn't happen.
I: What is the approximate percentage?
R9: It is different each time. This is not fixed as such. Range approximately sometimes 100% in the range, but it comes out negative.
I: Members of the collection come. Are X-rays done on positivity.
R: Happens
I: Happens for everyone for contacts?
R4: No, not for contacts
I: We are mainly talking about contacts instead of cases. You are saying sputum is done for everyone, in that it will come positive or negative, it is next.
R3: It goes for testing.
R12: Generally take place for contacts, not done for children. Sputum, because it is not given. They don't come.
R11: Testing sample by Gastric Labard
I: You are talking about pediatric patents
I: Are there any contraindications or eligibility is seen for testing? Or regarding starting treatment. What will you tell regarding this? First screening is done, testing is done. Right? As you say. Then treatment should be started next then are there any contraindications or who is eligible? For whom should we start, for whom we should not. Or related to drug is their availability or not what
R3: Basically, the big problem is drug availability.
I: Is this problem of drug availability came now or is it from earlier?
R13: No it came now only. Recently some months ago.
I: But before that I feel the drug availability problem has been there from 4 months. But does it used to come for contacts?
R4: Yes
I: Was INH there?
R4: We give INH
I: Was it given to patients?
R: Yes they used to give
I: What is your experience regarding compliance?
R3: Patient take madam. After telling, we are giving this so that TB doesn't happen; patient take. Because stigma is there in TB;s this and this drug is there so that it doesn't happen.
R13: There is a patient in your house. So if you take this for at least 6 months then chances of happening (getting TB) are very less. If we say this, patient take. Those who have an active patient, people from their home themselves ask that we want.
I: Okay. How much percentage is there in this?
R: No, means, it is seen they are taking regular.
I: What at your PHC sir?
R: They take.
I: Is scenario same that patient's contacts are taking and

R: Contacts are there
I: At other places?
R10: one case is there. They are illiterate. They don’t have that much seriousness either. In that three children are positive from their mother.
I: Okay
R10: Vijay Medical van used to come to do research on them in between last year. Their experience was such that she didn't look at her children. If it is happening to them then let it happen. Then look.
I: Means she did not take children any time?
R10: When they were positive, high grade fever was there, coughing was happening, then it was not getting well, at that time approach and from there onwards all these things started.
R: Such a case is there. It is not such that all are giving.
I: Yes, this only. I said that there are varied experiences everywhere? That is why I am asking that at your place because the population is different at each PHC. Their scenario is different. Then it will definitely be there.
R3: The time is gone in doing counseling. But this much time needs to be given to the patient in doing counseling.
I: Who mainly does counseling to patients.
R4: At OPD only. It is done. Yes, means doctors do, nurses do. Doctors only do.
I: Everywhere you do counseling or is someone designated.
R12: ASHA also do their place.
R13: Symptomatic patients are there from tribal place. Whatever people are there those people are fever symptomatic at that time when they are active that time only they give. Other times they don’t give. At such times ASHA are there for medications. They do majority counseling. Right at that time that you come. Because this thing will happen to you again and again. It will keep spreading. If we show fear then some people come scared. 80% of the population is covered at our place proper INH Therapy.
I: And for how many months do they continue? For 6 months or more or less?
R: More are not there, but less happens
I: What is your experience?
R9: They complete their regular. They Take
I: Do they come to take
R9: If gone out of town there is a problem. It is told by contacting where it is gone at that place transfer is made there or if nearby PHC is there then by communicating the efforts are made to have continued treatment.
I: Does the patient's contact come or does the patient come to take drugs?
R9: Sometimes patient come and tell us we are going from this village to another village.
I: Do they take while going or do they give wherever at village where they go
R9: No, they do there only where they go. They take there only.
I: At other places, means does the patient himself come to PHC to take like give me my drugs or they are given house to house through ASHA or something else?
R4: Some patient comes but ASHA tells continuity afterwards. If it is not possible for ASHA then delivery to the patient. Mostly relatives come. Contacts only come.
I: What at rest places? What at your place sir?

R5: Patient come if their contacts are not able to come. We have supervisors also.

**R13: We have one patient, madam. He has a phobia. He has TB but has anxiety. His relatives come in socialism and take (घेऊन जातात). Means there are such cases whose relatives take and go.**

I: This is about what you are saying regarding patient treatment. This I am asking about contacts. Whatever the contact's treatment is preventive treatment. Do they come to take their drugs or what?
R13: They come
I: Do the follow-up of these contacts is done and it's monitoring is done any form. What will you tell regarding this? Like our understanding is that he comes and takes. We feel It is going through His relatives or ASHA but in reality is there any system because of which we will know that his 6 months completed, he came two times.
R3: What happens is that treatment is continued, meanwhile the investigations which should be done like is there everyday's effect on body or what, it is not seen. Means it is not done from my side. Pharmacies close this after completion of 6 months (confusion I or R). No It is not monitored if this drug has an advance effect on that patient or contact.
I: Okay. At other places?
R13: Like we confirm if a patient comes. Regular trailing should be done. It is not done. Continuation when failure is there means if weight gain is proper
I: Sir we are talking about contacts. We contact. Tell in your manner if contact has completed 6 months or not. How do you take assurance?
R11: In reporting.
I: This topic is not coming in front regularly. Right? Testing system
R11: They have follow-up but follow-up is not being taken from the up system. That is why regular follow-up is not done for contact
R3: This much importance should be given to that. Like now TB patient's HIV testing, diabetic testing, their DBT benefit, all these things have rigorous follow-up. This should be 100% but that does not go proper. And second thing is training is not done madam. That is why the exact protocol should be followed for contacts before starting INH. Means everyone is doing how they feel as per their protocol. Now it should be remembered. NFT is done. It is not remembered because if there is protocol. We follow protocol for TB patients, this is not for contacts.
I: How many contacts come and take drugs behind every contact, what do you feel?
R3: If a joint family is there, then contacts are also more.
I: Tell one estimate. Roughly what is the percentage? If there are 100 patients then how much percentage can be contacts and from that how much come to take drugs? Or we will suppose 10, if 10 patients are there approximately how many contacts behind do come
R4: At least 3 are there behind 1 patient. Minimum 3
I: Yes. How many come?
R3: Madam, it is continued after telling once means they take after starting INH therapy.
I: Okay. Is there the same experience everywhere?
R10: Madam at our palace those who suffer.
I: those who suffer
R: 100% contacts don't come

I: Yes, that is what I am asking. How many come? Suppose 10 patients are there, means you are saying now 3 behind 10 patients, means 30 should come. Then how many come approximately?
R4: 50% come approximately.
I: Yes, it is okay
R4: It can be less from that also.
I: Yeah, we want approximately only. And these 50% came to take drugs then do you feel they really take 6 months regularly.
R: It's follow-up is not done.
I: Yes, follow-up is not done. But approximately you might be having some discussion and meeting with ASHA, then how much estimate, how many might be taking roughly?
R8: 20-25%
R11: Loss to follow-up is significant. Nowadays medicine is not available. That is why nothing like this is there for past 4 months
I: We discussed about this in the morning only. Has any resistant cases found at your place and contacts like this. Is their testing done?
R3: Testing is not done madam.
I: Contact's testing is not done?
R: Contact's not.
I: At that time how many patient became resistant due to bacteria TB patient MDR patient
R4: 2 came at my place
R: 1 came to me
I: Okay. Are such contacts coming?
R4: Their contacts now started at Talegaon only. They are mostly at higher facility. And because there only there with us. Their approach is very less. They come only for registration.
I: Means you don't have information regarding MDR contacts. At your place sir?
R9: They mostly refer only. STS. STS tell them. If a nearby medical college is there or Sassoon etc is there, they are sent there only.
I: Okay. Do you feel there is private support for this?
R4: No No
R9: They are sent to our Talegaon MIMER.
R3: No. Not in contact. If felt suspected of contact then we send for CBNAAT sir. Rest there is no support of them as such.
R: We send maximum to Aundh for CBNAAT.
I: Is such happening?
R: No madam. The entire team is there only. No even if it is there, practically support means we don't get feedback what next for us means for CBNAAT that much
R: Madam, they cooperate. Means if we say do investigation. They don't avoid us suddenly. It can be different at each place.
I: Okay. Is CBNAAT is being done
R3: Yes
I: Is TrueNAAT done? After that is TrueNAAT done. Is CBNAAT done for every patient or only those who have resistance.
R11: no no. Every monitored patient. TrueNAAT and HIV positive patient. Every patient.
I: Wasn't there a question of cartridge availability at all places?

R4: Ours CBNAAT is being done. Don't know about rest places.
I: At your place?
R: Compulsory
I: Sir, is it done at your place?
R12: Yes
I: Regularly?
R12: Yes, regularly
I: Where do you send sample
R: To MIMER Medical College
I: To MIMER?
R11: Maval Block's all to MIMER
I: How much time does it take to get a result?
R12: Approximately 1 day, 2 days, It takes 1 or 2 days.
I: Okay. You gave drugs. Then any adverse reaction came/was seen etc or adverse events happened, if such thing happened. INH is given to contacts and some adverse events happened something like that
R1: No No
I: Or drug interaction etc has happened. Or already treatment has started immunosuppressant or a situation like this has occurred? Anyone's experience?
R1: In patients or in contacts?
R12: It is in contacts. The color of urination changes in contacts. This is the problem. If it comes, then this only. Rest not as such.
I: then do they contact PHC that this is happening
R12: sometimes happens sometimes not. They tell when they come in the next cycle to take drugs?
I: Counseling is done in how much amount, that these contacts should take treatment (20:14-20:16 inaudible).
R12: Supervisor take madam. They go to house and counsel. Then those patients also send many times.
R: I don't know post name. STS.
STS is separate
R11: There is one separate for TPT. OC. They have done. There is an NGO. But we take absolute on that. We have also told Doke sir whatever is happening related to TPT. Means we have appointed a total NGO.
I: Not PPP actually that not TPT Private Public Partnership
R11: Yes Public Private Partnership. It will be genuine if private related to the Government. Whatever their appointment is not good.
I: Okay whatever these strategies of TPT, what do you feel how much they will be useful for TB elimination? We have to eliminate TB till 2025, this has come through central. What is your opinion regarding this and whatever these initiatives TPT. We are asking you this as an expert because you work in a field. And this policy which you have decided that TB will eliminate because of giving TPT, what will be your comment on this policy
R: It will
R4: It can happen. But how many years is the doubt

I: That is what we have to ask.
R1: Suppose if they isolate patient, the majority contagious policy will also reduce. Rest can be MDR, XDR, they will get cured. Whatever there are factors of integration this can be one of them.
R9: We cannot tell it's percentage, etc
R11: This will contribute to some extent to some percentage but this alone difference is not sufficient. And like how malaria was reduced, there was an entire full fledged army release. That way manpower is very less. This is very ambitious that it should be eliminated till 2025. Don't know how far we have reached but counsellors definitely and the active case finding for that doing current year (current system) is falling short.
I: Okay
R3: Definitely means we have a major issue madam. You are giving one whole department for malaria and this being a major issue, for this you do work in existing system. It has many aspects.
R11: Basically there is no dedicated trained staff for sputum quality check. On one hand you take malaria NT, LSO over someone. We have to force, make him understand with love that do. There is lot of waste risk from taking quality sputum. The examination is not done means clearly the sputum which are coming, it's quality examination ios not being done. Majority times, the STLS who is there is supposed to check 10% of negative. He has to sit and test the sample. Means he is not able to do his work. And whatever sputum sample we miss in that means canti (not audible) etc is there, it is not seen in that and focus is only on sputum pulmonary level. What about extra Pulmonary? We don't have anything for diagnosis. We literally have nothing. Extra pulmonary's contribution is also equal. Means that's been falling / causing rise in incidence for sure. But the rise that is expected it is also not getting detected because of lack of manpower. Trained manpower and whatever manpower is there their training should be done. Combined is also not done. THOs, MOs, TB supervisor, your lab technician, everything Whatever PH are there. You should address the complaints/difficulties of At least ASHA representative at PHC level, sitting together for this TB elimination.

**R12: TB elimination program on field, I feel there is more trend aptitude towards numbers. तरीका**

Even if sputum is 2, but the quality of patient which we need.
R9: The symptoms should be asked. It is sidelined and all numbers come down, this is done just to complete the target. Nothing second to this.
R12: Active surveillance, passive surveillance effective OPD and OPD's even if this. It is in the picture. Not even here till whole f=grassroot same picture is there. It is done till end grassroot. That is why quality is hampered somewhere. And the trend is not going.
R13: And where there are pockets, where we find this more, we are falling short of reaching our system there. Or we are falling short of breaking the belief, taboo there. So the counseling process is also at a lower level where no one has absolutely reached or it is not there. That community still resists
I: Can you tell such specific groups?
R12: Katkari group is more at our place. Keeping follow-up through them and to convince them is a big challenge. They don't do. Somehow getting them to the hospital even if we are talking return TB, even to bring them for pregnancy is one of the big challenge. They come for pregnancy through investigation. They absolutely don't come for TB.

I: Are there more specific groups?
R11: Whatever migratory population is there. And mostly the pockets who spread are of them only.
R3: High risk and all health related or it is not a question of the health department. If social economic standards are raised then these things will happen somewhere. How is it we are throwing from upper means these things should come from basic. Many problems are related to your diet, your nutrition, your economic status, many things are dependent on this. If get good food, get good things then why will bacteria will incubate in your body or spread infection. There is a primary complex in the lungs of all of us, but we don't get.
I: You people are quiet.
R: They are just pass out. Fresh pass out all.
R4: I feel in migration mining area which is there. There is a big mine on Nagar road. There is more in my pocket. Maximum TB patients of mine which come are from mine. They live for hardly 2-3 months. After that, where do they go, what happens to them (nothing is known).
R3: Means earlier I was in villages in the MIDC area. Now sir is there. There people in the village have made/built small small rooms to give on rent. There is no custom of ventilation. That is why patients are found in pockets which are there. And even if a patient is found and you give treatment, again he will go there only. The solution must be found on this.
Gram Panchayat also deals with these things. Are they building a home or what, something should be structural. In that, is this house or bathroom? Yeah, there will be a small window, from that how much ventilation you will get or what. Even if 1 patient is there, he will create 10 patients.
R4: It is same in the Mining area. There is so much dust. There is so much dust. That you will get 100% patient. With pneumoconiosis. Because of mining there due to dust. TB pneumoconiosis is very common and not only common, it is dangerous. Much crowdy area that is much. In small small 10 by 10 room, 4-5 people live. 5 very less, at least 7-8.
R3: What is basic root cause of all these things. If said only about TB then put in health related. On health department. It is not like that. If we see then many departments can get involved, but it does not happen. It is of health people, health people should see work.
R11: I think somewhere the level of motivation and convincing of health personnel is also less.
I: Okay
R: Means when I was in Ratnagiri, at that time the percentage of MDR used to be more as compared to regular cases. I call it as sitting on atom bombs, when will it blast, when will our future generations get affected, we don't know. Whatever now as much as is less, those vaccines whatever all sudden done, responsibilities to eliminate, we have to must do but manpower must be there.
I: What strategies you suggest?
R11: Dedicated staff should be there
R4: Dedicated and continuously they should get training
I: What more strategies you suggest sir? What more changes should be there so that may be possibility till 2025
R4: It will be out, but elimination might not be there in next 5-10 years, at least control might be there. Till those stages slowly slowly screening and diagnostic facilities are there for control are very less.

R3: Now if we have to have an X-ray then from where it should be get done? Not everyone has this facility. The patient gets skipped, he doesn't come back.
R11: We give X-ray free, madam, but for that patient to come to that X-ray facility availability, madam his number is coming and going. We have to pay transport charges Rs 100 to 100 Rs to him. If we pick up drop then their daily wedges are gone. It is known.
R3: And madam quality. We are doing it at RH madam. They won't ever give a clear cut diagnosis. Never. Only films come. Film is also not there of them. They send it somewhere. Then from there they come with it on the back page. No one gives a clear cut that it is a pulmonary cox infective etiology. What should we understand as infective etiology? Should we understand it as pneumonia or something else or should we understand it as TB. What can we do at MBBS level? We are sending patient to you for what that you give diagnosis to us. If no diagnosis then the patient has to be sent to private again. Like go, spend this money 2 Paise and come with those X-ray.
R11: What can be done? The government is willing they can give an X-ray machine and X-ray technician at PHC level. We do, but at RH, it is very mean 50-60% machines are not working. If the machine is working, the technician is not there. If a technician is there, then light is not there. Sir is laughing, to sir (31:03 to 31:13 inaudible)
R4: For research many kids at F1BC nowadays. Now I don't have an option. Then we have to send it to Sassoon. Month goes sir to get a report in hand from FNAC. One thing is basically when we say Sassoon then there itself quickly no there, feels negative
I: For the first time already
R10: That labour has taken money in basic advance madam from (his) owner. They don't leave his day after taking money in advance and the owner doesn't leave him without getting work done. Even if life is gone it's okay but the work should be done today only, but fill His all system facility are closed, he won't come even if we go behind him many times.
I: Guys, you discussed little less. Suppose MDR's in your PHC, suppose 10 patients are there in your PHC, in that is there 1, is there 2, how much according to roughly percentage. If we suppose 10 per PHC patient are there. Nowadays how many treatments are taking from that MDR, how many - 1,2?
R: 1 or 2 are there
R: At my place 2 from 16 are there
I: Rest on an average?
R: 10%
I: The objective behind asking this question is what do you give contact of these patients. Now suppose 2, 3 patients are MDR positive from 16. We have discussed from long time. Give INH to contact for 6 months. Right? Now then in our house in our PHC MDR's patient is there. What do you give to his contacts? You only tell. Do you give INH or not? Or you give something else?
R4: Only those who came for resistance. Their contacts etc are not done.
R3: How it happens, sir suppose if we say Maval, then those patients are monitored from MIMER. SPH are there. How is it that your PHC patient has come to us, registration is done from our side, patient monitoring is done from there. The patient goes there only to take drugs, means everything is going on from there only.
I: Do all MDR's go there only?
R: Means our follow-up is not done as it should be. Drugs come to PHC very less times.

I: Does anyone want to share any experience?
Do patient on regularly dialysis come to you?
R: No
I: Are there patients on dialysis or not?
R: For known case or other
R10: No no. There is no relation of TB. My staff is only of dialysis, means he is there, he does regular dialysis. Health assistant and HIV patient, because they have many benefits in preventive treatment to treatment guidelines.
I: Does anyone want to share any experience?
R1: regarding this, like how madam told propapine (puff) (inaudible) and (34:29 to 34:31 inaudible) should be given. He should be given Rs. 500 as lost daily wedges. Like we gave pick up drop free to them now with breakfast lunch and Rs 500 as lost daily wedges.
R1: This should be with approach. Otherwise the money which will be for direct, patient will at least get that. If it is given, then target based approach. But other side we are not able to give X-ray. We are not able to do that, but the calculation of output which is done, it is calculated on the base of target achieved. It gets very difficult. There are 100 reasons why the target is not achieved, then that in this X-ray component. At least rigorous analysis and there should be some provision progress, means it’s time to put more funds. Now the one who sponsors Nikshay Mitra’s initiative, they put on 800-900 official groups.They are doing good, but now by doing this TB eliminate. Food basket TB patient
I: Patient or family
R4: Given to those who come here. Patient is there.
I: But is it directly used by patient at patient that very
R: It’s monitoring is not done
R11: That is why what we did, we modified. In Maval, dates, ghee box. Which is protein rich
I: Calorie and protein dense
R: We give it to patient only. In food basket
I: Food basket
R: it is going from deposit sir
R: instead of whole family taking part in that, we give it to the patient only.
I: What are your experience while closing any TPT means TB Preventive Therapy
R8: I have one suggestion. Now suppose we are in a remote area. Suppose if there is one patient or of extra pulmonary TB or felt or suppose of pulmonary or if he gets extra complications then we refer the patient to Sassoon from here. And after going to Sassoon there is already so much load. That it is not like a patient will be taken in 1 or 2 hour. The patient has to roam full day. Or what I am saying is we near near or we have a private hospital nearby, it is also like that institute or hospital big big means the government should tell them or they should target such that whatever patient are these at their level something treatment for free or investigation should be done. So that if a patient goes to Sassoon one day, they are given a date on first day, that day , on that day they go there with expenses. Go by bus PMT, too much crowd. Then this day will go. All are labourers maximum. One thing we don’t get is a holiday. Think of us. If we want a holiday for one day then how much hardship we have. Accordingly they also have the same. Then too much time goes. Then if something like this is made available nearby so that if we make a phone

call or one chit is written. Like we did for sonography. ANC's MOU, if something like this happened related to TB then it would be very good. This much X-ray also not done, X-ray facility not available
R11: At the time of diagnosis if a monthly bundle of masks is given to the patient instead of asking to wear a mask. Mask price is 15 to 20 Rs. If we provide a packet of mask along with medicine. But the use spread of pulmonary TB is a little bit air borne. If that mask is given then he will at least use it. Means system will have work.
I: Cost is also not much
R11: Cost is not much. That should be added.
I: And if we take in bulk, then it will drastically reduce.
R3: Sir, that is what I wanted to ask, the preventive treatment which we are giving INH is a slight liver toxic drug, where its study is done. We are giving it to contacts then its toxicity is monitored somewhere. You have any topic, so I felt to ask
I: There are some such studies. But if we look at the cost benefit then still it is beneficial. But from first this madam is asking are its investigations done, then for that I ama asking because we should identify toxicity early. But unfortunately it don't happen anywhere. Did pregnant women came in your contacts. Active patients should be of TB, their contact should be pregnant, Did anyone came like this?
R3: Not like that sir.
I: It should be given to them also. It is safe even if it is. Even infants and it should be given to them also.
R11: Should we exclude those who have history of alcohol
I: Little bit. We should ask history in neighbourhood, if the wife is there then ask the husband. It will be a perfect estimate.
R: Then they should not be given at all?
I: For that first do test, Liver function test, wherever it happens there. Does anyone want to say more?
R9: The private's, now topic is being discussed. Madam the data HIMS should be followed properly. What is our command on this. From there also all diseases should be filled. Nikshay is there. Apart from Nikshay we fill private data MS/HIMS. Whatever points are in that then they should not at our place okay.
They should do on their basis because treatment also should be given to them. If suppose they convey a case , then see can we take a little bit of private.
R3: But without private contribution the elimination is difficult sir.
R11: We will need help of private practitioners. Sir over the counter means prescription of medicine is well known. If the diagnosis is done in a public facility then supervisor, 2-3 people visit then those who can spend then they take treatment from private. And which drug are followed, the seriousness is not to supervisors nor to private practitioners. Private practitioners. Their uniformity is not done ever. Training is done when, when they have read no idea, then it will change, then brand will change, this is there, this not. It will go on like that. So many medical stores are like that. Such information is given. It is not get from private practitioners. Though is some
I: Guidelines
R: It is clause

R: The one thing private practitioners are supposed to inform, but it doesn't happen. Notifications don't take place. That is why if their training is centrally arranged then definitely there will be help.
R3: instead of that if the government has strategies be should be uniformity in draft. Everywhere whatever will get that should be same. And actually what happens is that drugs which are there are costly. What and how are they. They go to private by fear. Then they cannot get it again and they live in between/middle. In that they get to relax.
I: Are such cases happened at your place?
R4: Many are there. They go to private, take for 1-2 months then it is not affordable to them continue then they come here. And their outcome regular checkup that is also not known. And they themselves also don't do. I think so treatment recorders.
I: then are CBNAAT etc testing, such things of such patients are done?
R11: if they come to us, then it will happen. Drawing/extracting information is difficult.
I: Practitioners
R: You have to be very critical with medical
R: Pharmacist
R: Those who have pharmacy. They don't have any regulation on them anywhere. At least it should be that whatever TB medicines you have sold their one record they should give to someone. Otherwise you will lose this thing.
I: do patient tell that they went there after coming
R: Yes, yes.
I: We took these tablets
R: Yes, they do tell. Some come with a roll, come with a whole file. Whoever come to us, come like this only
I: Then what about who don't come
R: not about them. Many significant part are there. Because how much we say, there is a lot of social stigma attached to Tuberculosis.
I: Okay. Thank you guys. I have a small request, all these guidelines are given on our website. Whatever questions I ask, be it MDR

# THE END!
