# Supplementary material for: Factors influencing tuberculosis preventive treatment implementation in India: A hybrid inductive-abductive thematic analysis using implementation framework
Source: PLoS One. 2026 Oct 14;21(10):e0359171. doi: 10.1371/journal.pone.0359171 (PMC13667825; doi:10.1371/journal.pone.0359171)
Supplement: Supplementary file 4 [file pone.0359171.s001.zip › Focus Group Discussions (Adult contacts).zip!/Focus Group Discussions (MO)/FGD_MO_4.pdf]

FGD_MO_4

Date: 24.05.2024

I: As sir told you know Tuberculosis related RNTCP program. Then as a part of this, the Government of India has launched Tuberculosis Preventive guidelines in 2021. Then have you heard anything related to this? Can you tell anything related to this? You can tell whatever you have heard related to this.
R: Anyone have not heard
I: No problem. Has any training been conducted for you since you have joined?
R6: Tuberculosis related NTP training was done in 2-3 years for Cardia.
I: Okay. Someone else's something?
R7: Mine was also done 3-4 years back
I: Okay
R7: Schedule was for two days.
I: Okay. What was the topic in training?
R6: Madam the one was RNTCP‘s conversion is done into NTEP and after that line of mode of treatment, pediatric doses, other dose and what changes are done in treatment. Means they have told about TB in detail.
I: Okay. Then was anything told about prevention in that training?
R: I don’t remember.
I: It's okay. No problem. What information do you have regarding prevention? You have just passed out. Then is anything told to you regarding this in your curriculum. (1:47 to 1:51 not audible)
R: BCG Vaccine is there, cross ventilation (.... Not audible). Close contact
I: Okay. Rest what else? And say something. Tell your opinions madam regarding tuberculosis prevention. Your opinions.
R: Sir told
I: Yes, told
R2: Nothing different.
I: Do you follow him?
R2: Yes
I: What do you feel?
R3: Same
I: Anything different? Okay. Have you done Pediatric prevention training? Do you remember anything related to Pediatric prevention? What is that?
R: It was told. Pediatric prevention means at that time only one thing was told that madam they that if there is little kid in house then the patient who is already in their contact they also should be given INH therapy 3 months. Means those who don’t have TB but family members who stay along with. Them along with 3 months. Same even if a little kid is there. It is told to give INH therapy to all.

I: Okay. We will keep little kids' aside. Adults only, regarding adult about what you are saying little bit we. We will talk regarding whatever you know, whatever you remember. Then you said one thing regarding adults that give INH therapy for 3 months. Do you feel some things should be done, any investigations should be done, is there any need to some things before starting this treatment?
R7: Regular tests should be done. Lab tests should be done, weight should be done. We start INH therapy according to body weight per kg.
I: Okay. What will you tell in a regular test? What investigations should be done?
R7: Lab investigation, TrueNAAT, CBC, NATRFT then cholesterol
I: Something else?
R4: We will do sputum, will do X-ray
I: And what will you confirm from that? What or what will you rule out or what?
R7: Means does he has any symptoms of TB.
I: Suppose if he has symptoms, then what will be done? This madam was saying something.
R4: That only that sputum examination. In that also, if it is negative then suppose if it is positive then we start our dose which is in new case
I: Okay. And if it is negative then?
R: If it is negative then for preventive measure
I: We can give or it should be given?
R: It should be given
I: Okay. What are you saying madam?
R5: Symptoms such that patients have. Can be TB preventive, generally if something like this is seen then investigation needs to be done. Then sputum positive, negative whatever will come. After X-ray investigations come positive then we will continue treatment for patients. Plus more advance treatment like TrueNAAT, at last we refer to RH
I: Okay. Does it happen here under your PHC? The patient has come for tuberculosis treatment but their contacts are there. You told investigations now, right? (You) told different investigations. Then are these investigations done for the contacts under your PHC?
R7: Yes, they are done.
I: Which are done?
R7: We do ARP
I: Okay, rest?
R7: CBNAAT
I: Not for patient, for contacts of patient. Yes their relatives' etc
R7: We do.
I: Is it done at your place?
R2: It takes place
I: Madam, at your place?
R3: it takes place
I: Sir does it take place at your place?

R4: Yes
I: Okay, suppose 10 patients are there under you. I will give one example. Generally, how many contacts of 10 patients are investigated? I don't want an exact number, tell approx.
R6: Those who are staying nearby.
I: Okay. How many will be them? If there are 10 patients then takes place for how many contacts approx 2 are there or 5 approx
R4: Mostly big family is there in the village. That is why 5-6 people are from home only.
I: Okay. Your experience
R7: Sometimes we exclude little children, take rest. We investigate quite a lot
I: Okay. How many contacts are investigated per 10 patients?
R7: Approximately 2-3, 2-3 per patient
I: At your place?
R: 2-3
I: At your place?
R: 5-6
I: Is there a different opinion of anyone or different at your place? Is there anything such different under your PHC?
R1: Not specific as such Madam. It varies according to family.
…..Inaudible
R3: ASHA workers are there. They have targets also.
I: Okay.
R3: They collect sputum targetwise.
I: Do they target contacts also?
R: Yes, contacts also.
I: How much is the target approximately?
R7: It is population wise sir. We decide as per village population.
I: Okay. Means they have also given the target of contacts. For sputum examination. Only for sputum or is there anything else also.
R3: There are others also.
R: No only related to Tuberculosis.
I: I am asking for sputum. Only for sputum.
I: Is it given everywhere? Do they have knowledge of how to do sputum collection?
R3: And they should have. What to tell patient that how to collect that cough and containers are given to them.
I: Okay. Where is the testing done of sputum examination?
R3: It is done at PHC level.
R7: DMC is there at PHC level at many places. It goes to STD mode wherever it is not available.
I: Okay.
R: It is available at all places at PHC level.
I: Okay. What is the condition of an X-ray?

R3: X-ray is not there. One thing is we function. We do at home (inaudible). Means designated center is given. We sent it there for an X-ray.
I: Are they TPT through designated at your level?
R7: No no TPT through (only).
I: Okay. You spoke about CBNAAT also some time back. Then where is it given?
R: At RH.
R3: Even if it is negative in sputum, if they have that symptoms then we send the patient to RH.
I: Okay. Is this service available at RH?
R: Yes.
I: Is it throughout the year or at any time?
R3: It is throughout the year. There are experts who test and all.
I: Okay.
R1: We send it to Sasoon.
I: Then, do the patient go there now? Basically, do the contacts go there?
R3: You told go to RH. For us if contacts come till PHC. Now sometimes they don't come and if they approach us then there is a facility of our van. We all message 1-2 days prior for X-ray that all these patients are to be send for X-ray. CHO are there, Community Health Officers or ASHA are there, Community Health Officers are there. They know that who are the patients in their community. Then they collect them, then we. There is a facility of van. Then by collecting in that van send it to SDH in an ambulance. They take.
I: Okay. Is there such a facility everywhere or any difference at your place? You said it is sent to Sassoon. Then how is the picture there?
R1: It depends on the patient. There is one patient of mine. He lives at PHC camp. He lives isolated. He goes himself. There is no need to give him a van. Nothing else. He himself goes there even if medicine is not available. He lives existing that is why has to go.
I: Okay. Now you spoke about patient. What is the comorbidity?
R: HIV patients.
I: now will you tell about their contacts of that patient
R1: He lives there isolate. Family is there, but it is not there
I: Okay.
(10:55 to 11:10 inaudible)
I: Okay means his family stays somewhere else. As you say.
R: They did not come to us till now.
I: Okay. Generally, what do feel approximately how many come for testing, patient's contacts?
R7: No one comes. Messages need to be given to them through ASHA that come and do testing.
I: How much percentage do come? 5,10,15 how much percentage does come after you give a message? You gave a message through ASHA that come. Speak, The madam at No. 4, you are not speaking.
R4: Means even if we give a message then also 10-15% come for testing. Most don't come. Like 25-30%.

I: Their saying is 10-15, at your place 25-30. It’s okay. There can be variation according to PHC. What is the situation at your place Parsi.
R5: They don’t come. They absolutely don’t come.
I: Okay. Then what do you do after they come for testing?
R4: Symptomwise examination plus lab testing.
I: Okay.
R: After that if they are positive then we will start treatment.
I: Okay.
R: And after that, we start preventive.
I: Okay. Are drugs available everywhere?
R: No, they are not many months back.
I: From how many months there is shortage
R6: Approximately 2-3 months passed.
R1: They came now a little bit. But not there for the past 1 month for that patient. That is why there was so much pressure. It was for 15 days.
I: Okay. Now 2-3 months was there regular supply before that?
R7: Yes
I: Was it for contacts? Was INH available or not? Sir, at your place?
R6: No.
R: Okay. Then was INH given to contacts or not. Means tell your experience under your PHC.
R7: We were giving regularly till drugs were there. After that shortage took place.
I: Yes it is earlier. Tell your experience.
R7: Drugs are not there, before that regular means we registered patients’ contacts. We prepared Excel etc as per how many contacts. We give by calling patient in PHC through ASHA
I: Okay. Sir, at your place?
R6: It should have been there. There is a problem because there is no medicine even for patient. Because medicine is for him. He is a patient from our side only. His weight is I think around 60. According to weight dose 4 tablets per day for him. This much dose for him at a time means we have to give strips also of 1 month to him. Then much shortage happens.
I: Okay
R6: we give weight wise. So because of having to eat more medicine we have to give more strips to him. That is why shortage happens immediately. Then a problem happens.
I: What is at other places? Madam at your place?
R4: We used to give till medicines were available, but currently there is a shortage from 2-3 months.
I: Were you used to give top contacts also?
R3: Yes, as per the testing.
I: Are you used to give? At your place madam?
R: Registers are prepared as such till it was available. All. If any patient comes
(Inaudible)

If it is there then refer then going till Sassoon.
I: Okay
Madam at our place one month of shortage of centration medicine shortage and patient suffered a lot. Means, some going to Sasson. Medicines were not available. Not much.
I: Now you tell before drug shortage. You said you used to give to contacts at many places. Then how much percentage generally used to take regularly. How many people might have taken for 3 months, can you tell?
R6: Ma'am Patient's relatives say they take, but we cannot trust blindly.
I: Sir, at your place?
R: They take for 4 months
I: Okay. Madam, what at your place?
Or is counselling really done?
R: CHO, ASHA Worker at ground level.
I: Okay.
R3: They almost take. Now we cannot tell 100% fully. In that they take.
I: Sir, at your place?
R1: Not given to any contact. 7 months took place, don't know before that.
I: Okay. From then, not given to contacts before that. Is there any support of some private practices or medical colleges in this?
R7: If a patient is diagnosed in our area then they notify. Rest medicines etc means if one of them comes then they refer.
R6: They mention on their reference sheet that start after visiting your PHC at this place means such a message is through them. If this case is detected then the patients are from here only. But they are detected there. In private it is such that you give start drugs for them. Then that sputum etc all is done there only. No, even if it is done at medical college then also they notify there. And then they say take your treatment at your PHC.
I: Okay. Then do their contacts come?
R3: No, we have to convince them i.e., contacts.
I: How many people? Now suppose you took counseling efforts for 10 people and as you said through ASHA, through CHO, then how many people are really ready? That say we will take.
R: Contacts?
I: Contacts
R3: At least 4-5 contacts come from 10
I: Okay. What are your experiences? At your place?
R: If ASHA do counseling in contacts etc then they (come) to PHC at least till 20%.
I: Okay, what is your experience? Where are you from?
R: Karand.
I: Okay. What is your experience?
R2: If we counsel patients, observe, follow-up with ASHA continue then I feel that patient comes.

I: Okay. Are there any pregnant women under your PHC who are cases, contacts? Is this happening or are there like cases where there are contacts from their house only are pregnant women or as you said does someone have co-infection, some have diabetes, they are on dialysis, is there something like this? Did this such case come to anyone's area?
R6: It did not come till now
R3: Diabetes etc is there
I: Okay.
R7: Or HIV etc can be there
I: Are these cases or you are talking about contacts
R7: these are cases. HIV, TB co-infection or diabetes and TB.
I: Okay. Which drugs do you give in such cases? What do you do?
R: We give drugs. If there is HIV, TB co-infection.
Infection and drug it's also
R7: Those who are coming to you at PHC not their Theirs is started. Started for theirs only
I: Okay. Then they don't come to you.
R5: They don't come for follow-up also or they don't come for anything.
I: Okay. At the rest of the places any drug interaction or any side effects took place. Did such a thing happen with anyone or some resistance such cases of tuberculosis or rifampicin resistance came or did anything such came or not? Or is there such a case from your place? Suppose if such case is there then what should be done for such contacts? Can you tell?
R3: No, mostly we don't do. We directly refer for MDR.
R3: We tell higher sample Aundh
I: Not even for Bhor?
R3: No, direct at Aundh
I: Is there any investigation done for whatever these contacts are there after giving drugs. Does this happen
R6: Not
I: Okay. The Government of India has declared that we have to eliminate Tuberculosis till 2025, then what do you feel about that? Will you tell your opinion?
R6: Actually my opinion is that at least 100 are there at this PHC. Madam, one CBNAAT machine should be available there also. Because of that time duration increases at SDS. There is no compliance of patient.
R7: Patients don't go there. There should not be any difficulty in giving TrueNAAT machine in 100+ OPD. Because after having 100+ OPD, there much patient's medicine is available there itself. If there is a machine then there is much benefit.
I: okay
R7: Because it does not happen that each patient gives sputum. They leave after taking bins, they say they will bring, some come some don't even come. That is why sputum is damaged in transportation. That is also one issue.
I: Okay. Madam, what is your opinion regarding this at your place?

R4: Means we should get even if we have 100+ OPD. Because no patient means even if sputum examination is there then also no. Some go to higher center, some don't go or some's whole treatment is also not completed.
I: Okay. What more do you feel regarding this?
R4: Vaccination.
I: Okay
R4: Adult vaccination. BCG
I: Is it come at your place of ICMR? No means right now ASHA sister and means did this that you do a survey of whatever beneficiaries are there.
R: It is happening.
I: Okay. What is your opinion madam?
R3: It is okay till test madam. It happened. Treatment took place. Shortage came after many days. Then these medicines are also costly. Do we have to order from outside? It is that much loss of patient also. There will be financial loss also.
I: Hmm
R3: Mentally loss also they are not fit physically. Such a shortage of medicines should not be there.
I: Okay. What is your experience regarding contact? Is anything preventive given to contacts and if there can be any participation of this in TB elimination or what more should be done or not, what do you feel?
R3: better counseling
I: Okay. Madam, what is your opinion? If you are seeing symptoms of TB, if patient comes to us, and even it is detected
R2: We give treatment to people from their surrounding and they take. Whatever I have observed till now, the problem is that there is still a taboo if TB (someone suffers) in a village. We should not have contact if they have TB. There is one family. They are also in much attachment that in their contact, that they are in counseling. That all these things. Then this only happens that after you have TB whatever that person is his/her mentality is disturbed little bit. Their counseling should be done. I feel counseling is important. Treatment and all these things will keep happening. But giving mental support to him is also important. Then there is fear in their coresidents also.
I: is it still there?
R: Yes. After saying TB, the mentality of people at the village place is such that their point of view to look at TB is a little bit different. Such things are still there at village place.
I: Sir, what is your opinion?
R1: Elimination goal is achievable, but whatever we are doing is okay. But whatever ASHA workers are there at base level, they should spread more awareness or more patients are detected and that much earlier we are doing vaccination, giving treatment, taking follow-up, doing all these things. But when awareness will increase then only patient will come to us. Medical officers are not going to search each patient. When a patient comes to OPD, we see that he/she has symptoms. After that treatment keeps happening 100%. Elimination is there but along with that more awareness should be there at base level.

I: Okay, you put forward the point of awareness then are there expertise in this? Is their training done that much? Can they do this much? Do they have that much trained manpower? Do you feel such?
R1: Madam it is a basic thing In awareness that do immunization. After that BCG vaccine is there. It is done itself.
I: Okay.
R1: After that information regarding cough that whatever symptoms you have, 7-8 days took place, 2 weeks took place, cough is still not stopping. It is productive. What should be done, give a visit to the PHC, they should tell the patient this.
I: Do you feel ASHA are trained in all these things?
R: Yes. These are basic things.
I: Do you feel they can tell?
R: Yes.
I: Okay. Then where is it lacking? They are trained, there is awareness in the community, then what is the gap in between? What is there? How can it be filled? What do you feel? What strategy can you tell, that suppose this sis done then it will be useful, or if it is not done then it will be good. Whatever it can be you can tell regarding this
R1: It is a whole hilly area where my PHC Malshe/.Panshet is located. There are no ASHA in each village. Most population is spread. Then such people are there. I feel ASHA cannot reach till such isolated families. There are somewhere community gaps which
Il: Is there more opinion of anyone that what should be done which may be help in elimination? Can you suggest something?
R6: the one thing you said regarding CBNAAT. If detection rate is increased then it will help in elimination.
I: Okay
R6: Because of the sputum sample, we used to take it at AFT also. It will come negative maximum times. But the TrueNAAT test at PHC level if CBNAAT is available, if it is showing positive then our time is also increasing than AFT and detection rate has also increased that elimination. But indirectly. Because many times it happens that AFB after taking AFB their sputum sample , in quantity in rate negative maximum rate it doesn't happen that we send a patient immediately for an X-ray and he/she goes and came back after doing X-ray. It doesn't happen madam because people first see how it will be adjusted in daily routine.
I: What is your opinion madam?
R4: Yes, detection rate should be increased. And ASHA in every village they are trained also, but they are not convinced. Some are in contacts, but they don’t come till PHC, that is why their detection is also left (not done). And whatever next treatment we give
I: Do you feel it will help in TB elimination after giving preventive treatment to contacts.
R2: Means those who are strongly positive or current one is X-ray positive and one is in his contact. If we give treatment to them, then the rate will be decreased.
I: Do you feel there should be some improvement? Do you feel some changes should be done?

R1: Medicines should be available at PHC.
R2: The most important point: Medicines should be available. Because patient get irritated they don’t take medicines also and he gives priority to his work. They feel TB is there so it is okay. Then patient’s mentality is shaped such, means side patient’
I: What is your opinion madam?
R7: Medicine should be given on priority basis.
I: Okay. Does anyone want to share experience regarding Tuberculosis preventive treatment or regarding contacts or overall regarding Tuberculosis program. Would you like to share any different case from your place as such or you can put forward if you have a different opinion. Sir, you want to something regarding this?
R: No madam
R8: Madam Velha block all. I am at Ambavale PHC. Sometimes medicines are not available there and most people are old aged. In that nollen will take an envelope. The envelope will come late. In that defaulter chances are more. If defaulters happen then most spread and when treatment starts for the patient then most of the times his condition is out and if it is told to admit the patient, they don’t go. Pure heart patient don’t happen because of the patient. One thing is whatever patients of Nikshay are there giving for them because of Nishay+Social commitment, this is there. We are on such patients only. Such patients area adopted. Immune deficient component are there at our place. Proper diet, high protein etc is given to him for 6 months. They mostly
I: What will you say about contacts? What will you tell regarding preventive treatment of contacts
R8: Madam we start only but they don’t take proper, they should take that. And they should do whatever it is there. They don’t have seriousness of this disease. That is why in society, they
R: Vaccination is there, BCG is there, I feel it will happen.
I: Does anyone want to talk? Want to share any experience? Okay we will stop here. Thank you!

THE END!
