# Supplementary material for: Factors influencing tuberculosis preventive treatment implementation in India: A hybrid inductive-abductive thematic analysis using implementation framework
Source: PLoS One. 2026 Oct 14;21(10):e0359171. doi: 10.1371/journal.pone.0359171 (PMC13667825; doi:10.1371/journal.pone.0359171)
Supplement: Supplementary file 4 [file pone.0359171.s001.zip › Focus Group Discussions (Adult contacts).zip!/Focus Group Discussions (MO)/FGD_MO_5.pdf]

FGD_MO_5

I: Madam, have you heard about TPT? If you have recently joined and heard about TPT in academics, you can also tell that.

R-3: Regarding that, I got dual responses, both positive and negative. 70% positive response. In this, I had the experience with a TB-positive patient who is on treatment. Currently, he is taking treatment for four months. I received a call from him saying, "Sir, there is news or information that there is a vaccine through ASHA workers; when should we take it and we want to give it to my relatives." It became a positive, and in the negative, it will be like COVID. Many people have joint pain or cardiac problem due to COVID-19: "So what will happen?"

I: Madam, have you heard anything about TB prevention?

R-1: Avoid spitting in public places. If the patient has co-morbidity, we should take care not to come in contact with TB. If there is a person in the house, stay away from him.

I: Home isolation should be done as much as possible.

R-1: Avoid going to public places.

I: Have you had any training?

R-4: The workshop has been done.

R-9: Zillah Parishad training has been done.

I: Can you tell me what points were discussed in that workshop?

R-9: It was expected that the Hon'ble Prime Minister's objective is to eliminate TB by 2025, so now we have decided to do TB vaccination.

I: Are there any other points you have done training so?

R-4: Now, we give TPT to infants to eliminate TB in advance. But they cannot achieve the goal as much as they want, so what is the effect if we give TPT to adults, too? For this, as many cases as needed in adults, so new method in adults,

if we give TPT, what effect can it have? This disease program comes for that. So we can achieve it little by little.

I: Apart from BCG vaccination, are there any issues?

R-2: We were giving INH prophylaxis.

I: Can you please provide more information about it?

R-2: If sputum-positive patients come, their close contacts are identified, and INH prophylaxis is given to them according to their weight. The program is carried out assuming they will not get TB again.

R-5: Madam, there are 4-5 criteria among eligible or eligible beneficiaries. In that:

- BMI less than 18.5 then
- If the patient has hypertension, diabetes, comorbidity
- The patient suffering from TB with a Minimum of three months
- Contacts of patients and the elderly above 60 years of age.
- Highly immune patients can be prohibited if they are suffering from HIV.

I: For what reason did you say these points?

R5 and 4: For adult vaccination

R5: Eligibility, we have also discussed….. like pest control

I: Earlier, they talked about INH prophylaxis. Will you say something about testing, when to do, whom to give, and when to start?

R5: Are contacts required to take INH?

I: Sputum-positive close contacts

R5: Only positive close contacts

I: Ok. Does testing happen for such contacts?

R5: X-ray and INH

I: How many do any other test

R: We do sputum test

I (asking to R1): Does sputum examination happen at your place?

R-1: Nor for contacts

I: Where does it take place?

R5: We do but those who have signs and symptoms and in contact. We see only on X-ray if sputum is available then sputum samples also taken

I: Are contacts X-rayed? Let's talk about contacts only. Any other tests can be done in your area excluding X-ray and sputum.

R: X ray and sputum test we do but not for contacts.

R8, R6: Not both

R6: X-ray we do

I: Ok, then you refer patients to RH

THO: After contact with the X-ray centre it will become free.

I: It is at the PHC centre. All are going to private for X-ray.

R: Yes

I: How do you do sputum testing or not?

R4: We do at *Aptale* PHC. At our PHC it is possible but some online problems occurs, so it stops.

I: Is it nearby

R4: Not here

I: Where it is?

R4: HDFS, not at *Narayangaon*

I: How many contacts can do sputum and X-ray? E.g., In your area there are 10 patients then how many are getting traces and how many can do sputum and x-ray. After that how many get diagnosed TB positive.

R: 8-10

R10: 10-15

R8: 4-5

I: Means there is variation like 4-5 and till 10-11. Then how many contacts get traced, those who are showing signs and symptoms?

R9: Close contacts means mother-father, husband-wife, children's.

I: This means those who are in contact with extra time. Then, can you tell me how much it is in percentage? Means in 10 patients 4-5 or 8-10 contact will be possible. So how many get traced like in percentage 100%, 50% and 10%. It can be shown through symptoms. So please tell the approximate percentage.

R4: 100% for contacts depends upon how many members in the family.

I: Does the relative come to you or how is the procedure to do this?

R6: ASHA worker takes follow-up time-to-time

R: MPW is also available

R6: Survey also happens

I: Is there any registry system available to keep a record of contacts

R: No

R4: Record of INH prophylaxis is available, indicating when the treatment has started

I: Otherwise no?

R4: No

I: For how many contacts does INH prophylaxis start

R4: If the result shows sputum positive, then 100% it starts.

I: If contact is sputum positive, then no INH prophylaxis?

R: No, no. If the patient is sputum-positive, we start INH prophylaxis for all the contacts.

I: To all contacts?

R4: Yes. The NGO was working on that, so it got a pop-up. 100% it is.

I: In what ways has the NGO contributed?

R4: They come up with patients to PHC, and after taking the patient's weight, they help start or initiate treatment.

I: Did NGO goes to your PHCs

R6-9: No

I: Which system at your PHC, how’s the ASHAs performance about contact tracing or treatment.

R2: Its good

I: Ok. What is their involvement?

R2: It actually starts to search patient. Nowadays symptomatic patient directly come to ASHA. By doing survey ASHAs find out patients and do their sputum and their X-rays. Also supports in the treatment once treatment get started.

I: What is the role in between contacts?

R2: After tracing contact, ASHA and MPW sends contacts for investigations.

I: Do contacts take INH? First of all, do you provide it to them via PHC? How is the process?

R2: NGO has taken that initiative.

I: Then that medicine came from PHC or NGO, or the patient came to PHC. How was the procedure?

R9: NGO supplies to them, and they came to PHC

I: Did the NGO provide drugs?

R9: Yes

I: You can tell the conditions of PHC for the distribution of medicines, whether it came from ASHA, Ngo, or the patients themselves, who bring it to PHC. Please tell the real scenario so that we can know. There might be varieties in this.

R9: I am new to PHC, so I don't know more about it.

I: Are you from which PHC?

R: …..PHC

R: Actually, sir, we didn't keep any follow-up

I: It's okay that you don't have an idea. How long have you been there?

R8: I will be completing three months now. MPW distributes medicines.

I: Ok, do they provide medicines directly to the home, or do relatives come to collect them at the PHC?

R8: Relatives come.

I: Does any patient in your area have resistant TB MDR?

R6,7: Don't know

I: Anyone who knows that there is a patient with MDR TB

R5: There is one patient under me who is under treatment for MDR TB.

I: Ok. Have you referred that patient?

R5: Yes, we referred them to our higher center at Aundh. The treatment has started from Aundh only, and we are taking follow-up of it. Also, MPW is taking the follow-up

I(asking R2): Are there any MDR patients in your area?

R2: Previously there was one patient in one family, we started INH treatment for that patient.

I: What you did to the contacts of that patient.

R2: Did sputum examination with those contacts. The private referred some contacts to us, but no diagnosis was found.

I: Ok, does any prevention treatment was given to them?

R5: INH was given.

I(asking to R2): What about the case in your area.

R2: They received INH prophylaxis from Aundh, and here we did treatment. For NDR the treatment was from Aundh.

I: Does any patient who is pregnant.

R4: No

R5: No any reporting.

I: Means no any pregnant lady came. If any are found then what will you do? If a patient is positive and someone from their house like sister, sister in law is pregnant.

R5: It causes stress to them and we will have to refer them to higher centre as MO don’t have that much knowledge that the contact will get effect or not by treatment. We think that specialized doctors can be better for such cases.

I: Does any cases of co-infection like HIV or diabetes hypertension.

R4: Previously it was but not now. HIV TB was there as per previous record.

THO: We were taking HIV.

I: What you did with the contacts of such cases, have you given any preventive treatment to them or not?

R4: No

I: Does any participation of the private sector for contact tracing, investigations or treatment.

R4: Patient findings, they came to us after starting treatment but not for contact

I(Asking to others): At your PHCs or your areas does participation of private practices.

R6,7,8,9: Not any

I: Any percentage that the contact taking INH. First of all how many months it is given.

R: 2 months (most of respondents replied 2months)

I: These people take these medicines for 2 months from you, but at actual how many months they consume it.

R3: Mostly they complete treatment as ASHAs taking follow-ups and we are asking simultaneously.

I: You give INH to contacts for two months but they might miss it. So what is the percentage of contacts that miss out the medications?

R1: 10-15% might not take it.

I: Does any test can be done after completion of two months.

R3: Suggests sputum

I: It happens?

R3: Yes, but didn’t come willingly.

I: Drug availability?

R5,8,9: Currently available.

I: We have asked this question as we are going to INH. For that we have to check the availability.

R7,4: Available

I: This situation happened currently or is it from previous

R4: INH is not provided regularly to us.

I: Ok, now the government of India has decided to eliminate TB till 2025, then what is your opinion about it as you all are expert in the field.

R2: I have little bit experience but I have worked 12 years in a tribal area. Previous years TB proportion is higher in some area but now in the same area 100 x-rays if done as a onetime activity, in that area there will be only one patient we found. Means TB proportion is reduced. Middle time expensive treatment was done and due to COVID contact is less in person, hence spread has reduced or other treatment may be received. Patients' social economic condition also developed also high protein diet advised. Previously X-rays were not free, now it's free and coordination of private doctors is good. Thus TB burden is reduced nowadays. This is possible to reduce TB by taking good initiative.

I(Asking to others): Your opinion.

R1: It will be done.

R5: Quite difficult as we still get patients. If one patient came in contact with more than 25 persons, so it can affect them too. Hence to eliminate TB till 2025 is less possible.

R4: Discipline approach is important in this. One patient can cause others, so if give proper treatment then we can reduce TB. Will have to look into the BCG vaccine, and also need to teach them for personal hygiene.

R8: Programs will have to be schedule

I: Will you think that you can remove TB

R8: Not completely

I: Challenges?

R7: Cases will find

I: But what is the problem?

R8: By my opinion its not possible. Spiting and cough is also the reason, our health system has to take follow-ups time to time. People has to be motivated. Patient has cough we asked them for sputum and they says no we don’t have disease, so they have to prone.

R7: Increasing population is also one of the factors. Complete eradication is not possible till 2025.

R6: Proper awareness and guidelines will reach the patient then it is possible. In tribal area it is started, 100% awareness will take time.

THO: Poverty is the main reason. In tribal, it was not done till now not for diagnosis and treatment.

I: What changes will you recommend in the strategies of the government of India for TB elimination till 2025. Any suggestions.

R6: ASHA or staff has said that people saying they have complaints for COVID 19 vaccine. So awareness is important. If THO office or TB officer can arrange such programs then it will be effective.

R7: Fear and eradication, otherwise TB is curable.

R8: Education for ground level and follow-ups after program

R9: If signs and symptoms are shown then patients should have to show, so that it can be curable.

R5: TB awareness is must, then proper treatment for active patients also to watch on contacts and provide them a proper treatment. This will help us to control TB.

I: Completely?

R5: Yes

R4: As human resources are lesser, there is one person who is controlling Taluka. Hence supervision is less. Dot provider payment for ASHA is not credited time to time. They all are labour, so it affect on treatment. So, these points are also measurable.

R3: People don't get messages that the TB is curable. People don't says that they get infected. Even If they take complete treatment, they don't say, also did not use protective instruments like mask. So awareness is important.

I: How much is the percentage that they don't even say that they got infected.

R3: 25%

THO: The proportion of children is also higher. Those who have labor, they also have some rules; most of them are underage. There is no awareness in that case, and working hours are up to 12 hours. So it impacts immunity.

R3: I have recently experienced one person undergoing a survey, who asked whether he had TB or not. He said yes, that he has TB. Then his contacts were traced. After that, he called me and said that people learned that I have, and they are all coming here. He was speaking for half an hour with me.

R4: No patient who is coming in front and saying that he has TB. This is the situation.

R5: I just want to add something to it, in our Marathwada area awareness is very less. One of my relatives is a TB patient, his family members try to feel him alone. No proper care, no food on time and kept the patient in one room only; so how will he develop his strong immunity. Proper diet or protein he didn't get as no one came closer to him. I quarantined that patient in one room, I did help to that

patient and talk with that to support the patient. Also said that I sat with the patient nothing has happened and guided them to help the patient.

R3: There was one patient who was well educated, well settled and never visited me as he thinks that everyone in the village will get to know that he is diagnosed with TB and had got tablets from us so he completed treatment from Pune.

I: Anyone wants to share any experience? No compulsion.

THO: People don't want to spoil the things, so go to private directly. My main purpose is that the government should have to provide free medicines, so that everyone should get it anywhere.

R2: Previously we started CB NAAT. In Ambegaon there was one machine later on it shifted so it was difficult for them to reach central place Manchar or RH place. So the number of machines or collection centre or manpower will be increased then it will be better for diagnosis. Previously X-ray availability was not there then we think that to wait for 15 days and do 2nd sputum after that X-ray. This stopped now and this is the good thing. We have to increase X-rays and diagnosis could be done through radiologist as it can mismatched with other general practices. There is unavailability of radiologist at RH when required. By providing extra funding to private these facilities can be availed. If a high quality nutrition kit will be able to provide then it prevent the disease to contacts. In rural areas patients will get payment.

I: That has been credited to their bank. Right?

R3: It do late, it can happen on the day of diagnosis.

R4: It happens after completion of treatement.

R3: It happens after a year.

R4: It credits complete payment but it should be credited on time.

I: Does TPT guidelines helps to eliminate TB

R5: Yes, it can be after vaccination

R6: If vaccination will be increased it will show a positive effect and decrease the number of contacts.

I: Are people ready for vaccination? What is your experience about it? You did surveys.

R5: 30-40% people are ready now but when we actually start vaccination the percentage will fluctuate.

R4: COVID vaccine news are ongoing, so it can impact.

I(asking to R6): What is the scenario at your site, are people ready for vaccination.

R6: Same scenario. People are ready but not sure for 70%

R4: I think educated people will be ready, especially from urban area.

I: Tribal?

R4: A little bit of a problem will occur. In urbanization people are already aware about diabetes, BP and they take care of themselves, thus they will be ready for precautions.

I: Anyone wants to share anything more.

R4: In child TB, malnourished children have more proportion of having child TB. They have extrapulmonary TB, so symptoms are not visible. They didn't get proper weight wain. We mainly focus on CBC, ECG, so there should be proper protocol to do compulsory TB chest or any other diagnosis should have been done. For not gaining weight this might be the main reason, but it is diagnosed late. So, the protocol should be formed that it can be diagnosed earlier.

I: Ok

R2: Now the point has come for child TB, so I want to share that BCG vaccine can be done at the time of birth. But most of the time our manpower thinks that why to do, this is a pack of 10, then why to use it now.

THO: But it is happening sir

R2: No, its issue of packaging

R4,3,THO: If it is possible to make it as a single dose, so it will be used.

THO: Our warnings are like that only one came, it should be go forward

R4: 10 dose can be minimized to 5 will also be fine.

R2: Wastage will be prohibited and availability will be there.

I: Percentage of periodic TB?

R4: Mostly

I: But how much?

R4: 4-5 out of 1000

I(asking to others): In your side

R: No. Mostly it happens that grandmother or grandfather has sputum positive, and child is at the age of 1-2 years but symptoms not visible at that time. After they reach to the age 3-4 years then they shows symptoms. Till this interval grandmother or grandfather got cured and later on family members forget that someone in the house suffered from TB and children got distracted.

I: Any other point. So, thank you everyone.
