# Supplementary material for: Factors influencing tuberculosis preventive treatment implementation in India: A hybrid inductive-abductive thematic analysis using implementation framework
Source: PLoS One. 2026 Oct 14;21(10):e0359171. doi: 10.1371/journal.pone.0359171 (PMC13667825; doi:10.1371/journal.pone.0359171)
Supplement: Supplementary file 4 [file pone.0359171.s001.zip › Focus Group Discussions (Adult contacts).zip!/Focus Group Discussions (MO)/FGD_MO_6.pdf]

FGD_MO_6
Date: 02.05.2024

I: As you know TB Preventive Therapy, an important guidelines came into existence under the National Tuberculosis Elimination Program in TB elimination program in 2021. First of all we will have discussion regarding these guidelines. We will start from here. Then do you know about TB preventive Therapy guidelines? Will you be able to tell regarding this?
R1: We start Isoniazid therapy for high risk contacts or relatives of TB patient in TB preventive therapy. Those who are high risk contacts or those who came in at their risk or those who are probable can acquire (a disease). We start Isoniazid treatment as preventive therapy for them.
R2: Means if small children i.e., below eight years of age are in contact with those whose treatment started, we start the way as you told. In that, TB Preventive Therapy was already started for many patients who can be positive in future.
R3: That's what I wanted to tell, we give this to children above six years of age, they are considered as contacts.
I: Do you want to tell anything
R4: If we keep an eye on time, if it is diagnosed then we can control at an early stage before it will reach lungs before hyposis happens. It can be controlled as early as possible.
R5: The investigation should be done for those who are in contact or small children. If they are positive, then we will be definitely giving treatment but if they are negative, then we can give preventive therapy.
I: Do you want to add anything
R6: There should be early diagnosis. If some are positive then we can give treatment and if negative then isolation etc
I: Does anyone want to add anything or does anyone want to tell anything or tell important points in this. Means just he told diagnosis, we rule out active TB or want to add any more points. Then we will move forward. I want to know whether you had training regarding TB Prevention when these guidelines came at that time
R1: No
I: No training as such
R6: No
I: Training
I: Means when these guidelines
R5: For RNTCP
R3: like we discussed these things, took place for RNTCP, but not this
I: Not like that
R3: Not special
I: Then at that time from where did you get this information
R2: we got to know this information means from the program.
I: Okay from the program. But was there a separate training as such or within program only
R2: Those who come to us like TB Unit people they tell such guidelines came. They tell us at that time. Discuss with us, at least once a month.
I: So you got to know at that time. Now we saw information about the program in general. Now we will talk regarding implementation, we will discuss means how it is implemented. Then you tell

one by one in detail what you do exactly? Means what you exactly do TB Preventive therapy. What do you do for household contacts? We will start from here this time.
R6: There are household contacts, there will be daily contact, but they should be kept isolated. Means actual patient should be in different room, isolation should be given plus tests should be conducted for all those. Rest contacts should be given treatment.
I: Means what is done at your place if there are patients?
R6: First we do a diagnosis of whoever is there, if there is a fever, we do an X-ray sputum test, if someone comes positive, we call contacts and do their tests. After that positive, negative. If positive then same treatment and if negative then negative.
R5:HIV testing should be done. Again as per their status and if there is some infection, etc. We do HIV compulsory and Blood Sugar Level, Hemoglobin these tests should be done.
R3: We also go to PHC level, symptoms etc. Sputum after X-ray. At PHC level, we do sputum for those who come to our OPD. After that X-ray. We do monthly review through the full staff which we have. We try to encourage people who have cough for many days i.e., those who are TB suspects to give sputum. Or other doing sputum at our place or preparing DMC or types of X-ray or there are referral units. We make an effort in this way.
R2: For new case, at OPD level like our we like we know that there are TB suspected cases like sputum, fever till 2 weeks, loss of weight. We tell these things in OPD or give whatever we message through ASHA. While doing this, if there are suspected cases like this, if there are patients like this, we call them in OPD. Generally, people are fear a lot if we say TB, they don't come out, are afraid, don’t talk about anything, etc. So it is necessary for us to find such patients. Some patients take treatment in private also but they hesitate a little bit to come to the Government. We give much treatment to such through our system. Sputum collection is a big topic which we take on OPD basis and suppose if it comes positive, it is necessary to isolate it as possible as. The reason for this is it can go from one to another through the respiratory system. So it is necessary to create awareness in the family and rest we do sputum through the system often. We also do X-ray in that. In that there are two things: pulmonary and extra pulmonary, it is necessary to focus on that. Because we don't see these things the majority of the time. Many things are detected in X-ray. In private also, many cases come to us, happens or of extra pulmonary, start some treatment etc., we should focus on that also. Starting two months whatever protocol is there is the treatment received accordingly. The benefits they get, whatever they get. They get MTC for food. It is necessary to see if they get those properly. In that he should think positively the more you can motivate and create awareness. There are some threats. If we discuss those with patients in a good way then definitely in future we can symptoms of TB
I: What do you do for household contacts?
R1: Whoever are suspects in household contact we tell to collect sputum. We have a dedicated DMC at PHC. After that If positive is found. The most important thing is the sputum we collect should be of quality. In that case regulatory of ASHA also and sputum of that kind is expected. Many times many people don’t give sputum due to stigma. There are common complaints from many places that they don;’t give. That’s why it is important to create awareness regarding this (through) ASHA or we also should go personally and motivate. And we give therapy according to age and weight to whichever contacts are there as per regular guidelines. We should start isoniazid. The more and more contacts which can culminate in severe form. We can prevent that in the early stage. The adult BCG vaccination is started as a part of this. One part of it is because

we have to eliminate TB as it is objective. The signs are seen that this will also be one of the effects.
I: We have covered many points. But I want to know contact tracing i.e., you have one index patient at home then what is the tentative percentage of contact tracing?
R3: It is 100%
R1: Yes,
I: Is it 100% everywhere?
R3: If it is not at least not the case but after diagnosis it happens majorly. It happens because of that.
I: That means contact tracing is 100%.
R: 100% everywhere
I: It’s not there. Means no diagnosis What is your experience, i.e., means if there is no diagnosis means not a case means after diagnosis most of it is there. Till now we used to do screening or rule out TB. Do you know about investigations like the IGRA test etc. that belong with active TB. If not, are there other tests, if symptoms are not there no symptoms at all in household contacts then is there any test or not to detect that. Then what do you do at such times means household contacts means some have symptoms means what do you do next. Contact tracing is 100%. Follow-up. Does anyone want to add more and whenever TB Preventive Therapy is started for someone, what is the eligibility? Your experience related to it. Eligibility means before starting, which patient from household contact? How do you decide to start TB Preventive Therapy?
R6: If there are symptoms, sputum positive
R4: If sputum is positive, X-Ray positive
I: Means now for TB Preventive Therapy
R: Contact?
R6: For small children
R1: Isolation method. If sputum positive, then for them also. And if they are older than 6 years, then I don’t remember for them or not for them.
I: Does anyone want to add anything?
R1: I am not sure, but it has Rifapentin. We ask eligibility means before starting if a patient is eligible for TB Preventive Therapy. Sputum positive means those who are positive. First means eligibility the confirm positive patients, tracing in their contacts
R: Okay. How do you do with their contacts? Now you know, you are handling in that house. You have a list of contacts. In that whom to give TB Preventive Therapy whom to not related to that means is he/she eligible for TB Preventive Therapy
R: What do you exactly mean by preventive? (voice unclear)
R: Preventive means TB patient is there in house household patient, it’s household contacts will be there.
I: Ok then, what do we do with them
R: At first, we will do their screening. We will start after deciding their eligibility and all.
I: We are talking about eligibility, means does anyone want to add anything? Can you talk about TB Preventive Therapy or what is your experience?
R7: Actually, the first criterion should be positive patient for prevention. It should not have active tuberculosis because if active tuberculosis is there and contact is for tracing, then there will be therapies for both. First we separate it out then we make sure that patient is not positive.

I: first we will listen to them and then we will come to you. We categorize patient like the one who is in contact tracing, i.e., if its known case of tuberculosis patient and it's in contact of XYZ patient then we confirm that this patient should also be not positive for active TB. Because if we categorize both the therapies between active Tuberculosis and only contact tracing, then they are different. Because of that, if we see, the criteria for our patient is cough should be there for more than 15 days, fever if sweating is there, otherwise we consider things like BMI. If it is there in that, suppose if the patient is malnourished then we can start. Suppose if a patient is aged, they have chances of Tuberculosis, then we should consider them also. Just look at that, that is not positive. Because if positive and if we give preventive therapy then these both will be mistaken. Contact tracing and positive patient are different things. Means active TB should be ruled out first.
I: Also, does this TB Preventive Therapy have any contraindication? Do you know anything about contraindication? Means we saw whom to give but we should not give to whom?
R6: Pregnancy
I: He/she is saying that we should not give in pregnancy, what do you think?
R: Actually No. There is no contraindication like pregnancy as such for this.
I: He/she told one contraindication Active TB, other than that is there any means do you know?
R: No
I: Then, do you do assessment and counseling before, when you have to start TB Preventive Therapy? How do you do that properly?
R7: Actually many times we see that Rifapentine doses are high doses. Due to that, there are things like nausea, vomiting many times. Then the patient is like that. Whenever there is one of the patient in that family, then already we know the things like weight loss happen in TB. After that, due to high doses of medicine, recurrence of these things can happen in this. Because of that, the patient's relatives say no. Even in my PHC people refuse to give sputum. If it comes positive, it is our fate, and if not come, it is also our fate. It is for the patient. Due to this, even if sputum is seen, they say no to sputum tracing also. We even have many pending X-rays. They say no to X-ray also. In that how it happened to the Corona vaccine. If we live, it's fine, and if not, it's our fate like that. That's why it often happens that they neglect while taking medicine, unless we do a lot. Means no means there are consequences. There should not be complications of TB. So, we give medications for prevention. For that we have to tell them, because of these TB medicines persistent TB should not happen because we know that even though lungs are the primary cause many times that should not be active TB undetected. Even Malaria we say means TB brain we say meningitis etc that presentation of patients is after a long time. And many times it happens that it is seen in patients after 60-65 years, so they say now what is there in our life. So take medication again and continue. So, we see all these things at primary level, then we try such in patient's counseling like. There are 13 patients in my PHC. Then 13 patients are positive. The rest must be undetected patients. That is why, we tell patients that you will have to take whatever these medicines till 6 months. That is why you will be fine with whatever complications will be there afterwards. Other than that, your family in the house, whatever little kids who go to Anganwadi or pregnant women. Because after treating these people we can have complications. Because we know that if we take Isoniazid and Rifampicin dose then because of the kid it can happen to the fetus. That is why we prefer that apart from the patient, their relative don't face any trouble. Little

babies or pregnant or aged people are there because to console these people, people have to hustle here and there. Because after Indapur, Baramati patients have to be sent directly to Pune. Because the patient, instead of spending 1 Lakh or 2 Lakh, take benefit of whatever you are getting from the government. We have to convey in this language only to the patient.

I: What do you do in counseling means (counseling etc for) actual implementation counseling etc

R1: The most important part is stigma. We take follow up of patients through ASHAs to remove TB related stigma. Whatever may happen, patient should come to us. If sometimes they don't come then we ourselves bring that patient by sending team and do counseling by one way. Like it has happened to this person, maybe you will be there tomorrow in that lane, so if you take treatment on time only. There is a change in quite a lot of people,. Some people take. There may be defaulters at many places so, we cannot do this sin them. But we take quite a lot efforts. In that counseling is important, means it is necessary to pursue them. Even to suspects that there will be long term effect of this on your body, so you should take care of yourself.

I: Does anyone want to add anything?

R2: In this, the important thing is to give confidence to patients that TB can get fully cured. Many people fear that quite a lot of mine is over. But other people say this is a fully curable disease. If full treatment is taken carefully then it can get 100% fully cured. It is necessary to tell If we take that treatment partially then what are the side effects. If medicine is taken according to whatever protocol, then it can get 100% curable. TB should not happen to others from him. Then you have to take yourself, how to keep little kids, pregnant women away from him. In case of your diet one fear is reduced and in initial months if weight is gained then he also gets confidence because of that medicine. Either way there are so many medicines that he gets scared by seeing only how to take these many medicines. We can reduce this fear of him by counseling etc. Then he also gets confidence that if his weight is increased, the more confidence is created in him. In this way if a patient is treated by doing (continuous) counseling then we can tell others also that earlier this patient was like that. Now he is fully cured. His illness can be fully cured. In this way. doing counseling to them. If pregnant women are there then taking their care. In the case of little children, there is other that extra pulmonary if little awareness is created then it can happen.

R3: Means this TB counseling, similarly those household contacts who are there more in TPT counseling will get in home only. Don't have to go outside. This is one benefit to them.

I: Do you want to add anything?

R5: Counseling

I: No, we are talking about medicines of TB preventive therapy. Let's talk about their availability. How is the situation? What is your experience in your health facility?

R7: Yes. All are available.

R3: There is a problem now. Last time Saheb said to us that he did adjustment.

I: Are they available now for TB Preventive Therapy? What do you usually use?

R3: INH

I: Then is this available?

R3: Yes

I: Everywhere? At your place also? And Rifapentine
R1: Rifapentin not available
I: Now these relatives of patient are there, you start TPT for them then do they follow up. Does their monitoring, follow-up etc take place properly?
R3: Yes it takes place.
I: It happens through who?
R3: Mostly ASHA Worker. ASHA Worker, MPW.
I: Are they home visits?
R3: Yes. They are home visits.
I: Do you want to add anything more? Now we know those guidelines. But what do you feel what is the feasibility of these? Means is this all feasible? Means we discussed all TB Preventive Therapy like contact tracing, eligibility. Ruling out then after actual counseling we start TB Preventive Therapy then they taking actually then the compilation of TPT then their monitoring & follow-up all this. Then what do you think is this feasible? Is this being implemented?
R: Yes, it is taking place
I: Now we saw what are the guidelines in general and how you actually implement it. Now the challenges in it. Now we will discuss when you start TB Preventive Therapy for any household contact then at the start at which which levels and what what you do?
R7: Patient refuses for sputum collection. Means the rate of household contacts is less at our place. Means literally one patient is there. My ASHA worker was telling means if we even go to a TB patient's house they won't even let us stand there. Something or the other happens so it is difficult to monitor. My Shevgaon is always below in this indicator. Because how is it means corona has impacted a lot madam. Because of that patient sir we have an issue. What to talk about that madam. Our time sir also knows. Means how is it one patient does this to even stand. They are TB patient. The second criteria is that the patient is no more patient now. Every patient is a doctor. Mobile happened a lot, that from small children to all. They say that even after coming to hospital madam we ask what is your presentation? What is troubling you? They won't tell. Give me an injection, put saline, give me that. Let us take the BP of the patient first. Patient what more whatever it may be. That is why we face difficulties and will keep facing them. The patient refuses for sputum that you for screening. For screening
I: What do you feel?
R1: Important is awareness in people. Means the information should reach them that we have TB Preventive Therapy, they should also know. Our team is there. But it should reach them. Many sources are there. Our team takes follow-up from time to time. Many times other programs also start. Various programs of TB are for our ASHA worker, ANM so the patients from there should be aware, awareness should increase in them that we will get benefit from this preventive therapy.
R2: This is an important topic that patient should have information. Just now as stated, patients should know various things from various sources. TB is not new now. But it can get fully curable and they don't have much knowledge about its complications but it is necessary to tell them that preventive medicines are there we can save them etc. It is necessary to tell them that there are

diseases like TB, Malaria in small children. In that to select sputum. There is a problem of sputum collection everywhere from starting. How much we take efforts patient don't get ready in positively. Counseling should be in a better way. Other programs are ongoing so in a way we are keeping watch. But we are not getting the results we want. And now we also have expectations that it should happen. And all these things are challenging.

I: Do you any

R4: Awareness in patient's relatives because they will behave carelessly. They say anything like doctor nothing happens to me. They don't understand anything. Their mentality. They have to go for work. Those who go to work daily leaving all this and travel what about them? Even though it is said nothing happens to us means these people have carelessness. Awareness should be created in these people. After a patient is actually diagnosed we give medicines and do counseling.

R6: Talked about medicines. Antacid etc so that there are no side effects. If there are no side effects they will eat with interest.

I: Now, do you want to say anything?

R5: Challenges. There is a problem in counseling also. In short regarding contacts.

R7: Many times there are problems because of migration. Many times it happens that started for one month. Be it TB. After some time we say patient contact tracing doesn't get this happens many times. Have to roam here and there for patient.

I: Is there adequate staff available for all this?

R5: Yes. ASHA Worker.

I: Everywhere? And their training is also done?

R5: Yes

I: And contact tracing also takes place properly as you said? And those which are vulnerable groups like pediatric, pregnancy or HIV. Are there any challenges in this? Which TB Preventive Therapy you give to them? What are the challenges? If we tell earlier, in little children, do they accept or have you seen such patients in pregnancy? What is your experience with this preventive therapy?

R2: Mostly not

R3: Pregnancy after counseling

I: Any problem with little children?

R7: When I was in internship there was one patient. One HIV patient was there, means he had many diseases like from tuberculosis to all. The patient was in a good facility, still he was like everything is happening to me. Fungal infection happening. Taking Any medication which was not suitable. The patient felt now I don't want anything. Many times it happens that TB patient and HIV patient go together. If immunity is reduced then taking Tuberculosis treatment or HIV treatment, these both are higher therapy. Even though we give for 6 months continuation or tracing these patients, telling them is good. Counseling or many times other illness occurs with these two diseases. There are challenges in such patients.

I: Is the supply of medications regular?

R: Yes means TB Preventive Therapy Isoniazid all are regularly available. All are available Weightwise Pediatrics.

I: We will talk about its compliance. What is your experience? You start TPT for household contacts, is there compliance for medicines?
R2: Yes, they take after we tell them. No difficulty of them.
I: Do you want to add anything? And side effects means when you start Isoniazid about its side effects what is your experience?
R2: Means if we awareness patient and tell first that it can happen in this way, effect on appetite. If we tell them at first only and properly almost then they won't even refuse.
R3: And these things are going to happen. Nothing much. Mild nausea is going to happen due to medicines. There is nothing severe.
R1:Means after starting nothing like that means we tell them previously. Means if it happens. Because the patient is not scared if only. R6: It is somewhat risky because they will get to know
I: Now we will talk a little bit about drug resistance. What is your experience regarding that?
R7: There is drug resistance means now MDR XDR, all about immunity and these bacteria. Every antibiotic acts on genetic level Means Rifampicin, Isoniazid all these transcriptions we tell after start which are all on these levels. Those who consume new antibiotics like changes occur in the on genome. Like there is in HIV we see that it changes right away. Likewise we see in TB. Like it is seen in *Mycobacterium Tuberculosi*s. We see atypical mycobacterium in that, that's why many times drug resistance happens and many illnesses come from TB. Like we talked some time ago, the body creates something new to destroy in one disease like HIV, it's because of that.
I: Does anyone want to add anything? And all these treatment outcomes means complications again treatment outcomes are at this level or any challenges
R7: means recurrence rate that we say 1.5 million people will die every year. Means the treatment we give that prevention. The patient is very clever. Like I said some time ago, one can tell by looking in the mobile. After we take this treatment then this is happening, this is somewhat difficult to tell. As per our medical faculty, we always talk to PHC in a positive way, tell many things along with that, then it is difficult to trace if they really take treatment for continuous 6 months means there is a problem in case of compliance.
I: Does anyone want to add more? And what is your experience with those who are patients from private sector
R1: Reporting. In case of reporting we have even if someone completes treatment from private sector reporting is not done. This is the main issue. Rest nothing else.
I: But how is the acceptance of this for TPT? The patient's private sector means of household contact, does counseling take place in them or not?
R6: But medicine Rifampicin. We tell ASHA workers to visit households. But we don't know if Mod Auxina takes place or not.
I: Do you feel anything different regarding the patients in private.
R6: People in private should tell us. Then we can do counseling by going home. Private one cannot visit by going to households or cannot do anything. We can send ASHA worker. In private, burden means one regulatory. If PMC conveys us then after going home. If they make at least one call that this patient is there.

R3: If it comes in review of ASHA then only. If it is missed from them and if it is complete from private then mostly and mostly they don't tell in private. Even ASHA means. If they had done the check up in private.
R4: And if they have taken treatment from private then don't tell in public due to stigma. In their family only.
I: Does anyone want to add anything?
R2: The treatment of patient is ongoing. Many physicians tell us to start etc. It is not like that we have a problem that we get a good response. Rest of the things are there follow up, etc fully. In that only such people who afford follow-up.
I: Will you tell your experience means the implementation they are telling?
R8: I was listening. Sensitization of physicians was done. In some places if we see, in other blocks we see, they feel that our regimen is right. Government's routine. Both these things. One Patient and the economy are their shellfish. They feel if a patient goes there then little bit it can happen. They tell take this treatment. We give it to patient. Then they don't come to this Taluka. They don't come there if we specifically see because we are sensitizing them frequently. Then this sensitization is very important. Because if we pursue them. No notification is there but why don't we tell people. One level family has a taboo that we should not tell about TB patient in our house. Their mentality was not there before. In that, if a private doctor supports even a little bit they don't come to our system. Everywhere name will be there or people will get to know if this is what happens. I don't think it is different like that. There is no problem in treatment follow-up if there is no problem at all. After notification remaining quite a lot follow-up also happens. Because at the time the patient comes to the system. Even if it is left from us then also in STLS from a health worker does tracking. Patient notification is only important. Rest all take place properly.
I: Now we have discussed challenges quite a lot. Then how can these challenges be overcome? You all told what the challenges are. What do you feel? What can we do in that case?
R5: If someone is taking treatment in private then we can tell the same medicines are available in government. You get that free. Means I shifted one patient to government treatment who was taking treatment in private. Means his hemoglobin was less. He was treated with Iron supro injection from PHC. for Hemoglobin. And whatever difficulty they had medicine. I made him understand here itself and he took the whole treatment properly. Means we do counseling for them like that.
I: What is your experience?
R1: That's it, counseling. Because the way we keep doing counseling to them because in private It is like that the expenditure is more than their income. Tablets, then X-ray, then one of the. Test in the lab continue for 5-6 months. If we tell them by counseling then the same things happen here also. A little bit of money goes but mostly your 90% all is recovered. If we do counseling for them and if a little bit one positive thinking means those which are poor people all this can happen. Their benefit can happen. That see Baba treatment can take place.
I: What is your suggestion or what can be done or your experience?
R3: Sputum collection should be increased so that investigation cases should not be missed.

R2: Whatever is told financially should be persuaded to patients. This medicine if started in private then what is the expenditure and in Government same treatment, same drug. If it is cleared to them that the quality of drugs is good and from now onwards if something has to refer to them or the next facility or It is very good in Government so that they can be treated properly. If some complications occur or repeat infection is there in 4-5 years then the next expenditure for inspection it is necessary to tell this in a good way. Because challenges are lot. We have to run a program and it is necessary to think in a positive way. That's why I feel that we have to accept whatever face things will come in case of treatment or screening. And it is necessary to support all our staff. That this disease is fully curable and that it can get cured because what happens, sometimes we are tired. The program has been running for many years. People think that things are the same. Like when polio happened the same dose was given. Treatment is also given for many years. Leprosy is there. Leprosy has been so many years, still the patients of leprosy are found. That is why it is necessary to update knowledge of staff like we update in training.
There is one challenge that awareness is an important thing in TB preventive. We saw in the Corona period that people did not know in the Corona period earlier. But the time at which mass happened that time people were out to take vaccine. Earlier there was stigma for 2-4 months. But till there all with responsibility means cumulative is one part. Awareness through one part. And second is private facilities. Then many times the patient gets reported after treatment is completed. The timely reporting is important. Means we can start preventive therapy after we find positive active TB. If we get to know afterwards maybe our team's follow-up also comes in it. If its reporting also done to us it is small challenge but it can be rectified.
R7: Mass education should be there. You go to any level in school we in 10, 12th we did not know about TB like disease. Information in the basic system. If we can include some in books or even camp like we take eye camp, monthly cycle (periods). We take all these things at the school level like that if different health related programs are launched or one day that school or if it happens at that level then people also more. It happens that when little children tell parents then it is remembered immediately. That is why I feel all these things should be in mass education. Right from school to all.
I: Any suggestions?
R8: Suggestion is now our all is system oriented more. Means as told counseling is important part. Now 2-3 systems are oriented that now we how proper medicines should reach patients is the responsibility of our system. The second point is the lab technician which we have means we say elisoper they are the majority of the malaria department. This is the real problem. Then what happened to them that their mentality is not like that still like TB is also our program. That is why the hesitation which is happening for sputum check Actually main notification is the only weak part. Rest all we are doing is tracking all giving treatment. Private also notifying now. We are giving money to them. We are also doing X-ray from village to village. It is necessary to give stress to this part frequently. As much as sputum will be checked there because one part of the people are hesitant to take that. The second point is we all also now tell night and morning once the patient goes from here, gave sputum containers to them, his point of view changes. That's why

taking a spot if I feel patient is symptomatic because don't know if he will come tomorrow or not. Because the majority of patients are poor and are from here only. That is why if we use container word, they don't come here. Means majority they go missing. Spot should be given. This is necessary for diagnosis. Because if we take a spot, the mobile number comes, the address comes. Means we can trace the same tomorrow. It is important for notification. And at those places like we did DNC at PHC. If whether sputum examination is done these are system oriented subjects. There they should conduct a meeting of lab technicians frequently. What they have to face because once they start checking then notification increases. Most weak point is there because what happens even if they fix slides then who will take that at our place. Transporting to Thane, then there comes a topic of transport. And it is lying there only at interior PHC. Then it happens like that only. If this one drawback is removed then it is running successfully. Because ASHA are also taking follow-up patient to patient. We are tracking from Nikshay so it is not a subject of getting missed. The diagnosed patient is not missed but the diagnosis is wronged.
R5: One thing we will say that TB detection TB patient the one topic comes in extra-pulmonary and early investigation.
R8: Because of that these people are missed. They are not available anywhere at our place. This extra pulmonary one topic comes that is a problem for diagnosis. And investigation is not available at our place. That is why these people are missed.
I: Now we saw challenges and strategies to overcome challenges. Now we have come towards the end. Now I want to know what is your sentiment (□□) towards TB elimination. Can TB get totally eradicated? What do you feel?
R8: Yes it can happen.
R5: But not the target of 25. Public awareness. Counseling of people on a large scale. This is the main part and awareness should be there regarding that. A positive approach should be there.
I: can it happen by 2025.
R6: No. No. It will take at least 2-3 years.
R8: If we take lots of effort then it can happen. It can not happen. 2025, 35, 30 it will keep happening. We will say this is an endless process. At the time when the target there will be the last stage near like Polio we frequently increased frequency of that. We caught targeted population. The TB Mukt Gaon program is also good. That is also the majority. The important thing is that when we want to eliminate, only the health department should not be involved. The sectoral means when whatever departments are there remaining all their coordination involvement at that time elimination will increase. These are interrelated topics. Gram Panchayat or Anganwadi all these when interdepartmental will involve at that time elimination will take place.
I: And what is your sentiment that this TPT started guidelines came? Does their implementation take place? How much of its contribution will be there for TB elimination TPT.
R8: There will be definite benefits of TB Preventive therapy.
I: Does anyone want to add anything? Experience etc.
R8: Experience is such that took positively and gave treatment to patient. There are man patients like that. I was MO 5-10 years back. The patient of that time met us. They say we are cleared. Such

experiences are also there. If we take follow-up of TB related. Actually there were 4 patients in one family. His mother, father and son all were boycotted in the village, but all 4 were cured. The mother expired due to age but the rest of the children do good work now. Means if we look at it with a positive approach, then these patients can live in society after getting cured. This is important. Our mentality is a little bit like this. If we do this by good heart then there will definitely be benefit and their economic burden will also get reduced. Because as long as there will be cough and if it is not diagnosed timely then they will take treatment somewhere. Means money is also wasted at private. Complications also increase. After complications are increased then they have to take treatment again. If they have to break this cycle then this elimination.
I: Now we all discussed all guidelines, challenges, and implementation. Thank You!

THE END

Translation using AI

Patient:

- Brain: We say that the primary cause is tuberculosis.
- Even if the patient is cured, the side effects of the medicine remain.
- The presentation of TB side effects occurs after a long period. It is often seen that after 60-65 years, the patient starts to have side effects.
- The patient then says that now what will happen? Will I continue to take medicine for the rest of my life?

- All these things are very common at the primary level.

· I try to understand that there are 13 patients in my PHC. Out of the 13 patients, 13 patients are positive. The remaining patients are under-treated. We tell the patient that whatever medicines are there, they will have to take for 6 months. Therefore, you will be saved from the complications that will occur later. Apart from that, your family members who are young children or who go to Anganwadi or who are pregnant mothers. They should tell these people that complications can occur after treatment. Because we know that if you take the dose of Isoniazid and Rifampicin, etc., it can cause complications like fever, etc. in children.

· Therefore, we prefer to tell the patient that they should not leave the house. Because complications like fever, etc. can occur in children.

· We prefer to tell the patient that they should not leave the house. Because complications like fever, etc. can occur in children. It can also happen to pregnant women or elderly people.

- Line 1: "This is the reason why people have to run around."
- Line 2: "Because, if we say to send the patient directly to places like Pune from Indapur-Baramati,"
- Line 3: "It means the patient also shouldn't have to run around."
- Line 4: "And the patient is like, one lakh here and one lakh there..."

Section 2

- Line 1: "...This is why you should enjoy the free treatment you get from the government."
- Line 2: "Instead of doing all this, what you do is called counseling."
- Line 3: "In counseling, the most important part is stigma."
- Line 4: "To remove the stigma related to TB..."

Section 3

- Line 1: "...the most important part is..."
- Line 2: "...housekeeping"
- Line 3: "...stigma is. To remove the stigma related to TB..."
- Line 4: "...if we follow up regularly through ASHAs..."

Section 4

- Line 1: "...and the patient..."
- Line 2: "...should come to you. Sometimes, if the patient..."
- Line 3: "...doesn't come, then you yourself..."
- Line 4: "...should send your team to bring the suspect..."

Section 5

- Line 1: "...and examine them and give them..."

Counseling is a process of giving advice and guidance to a person. In the context of TB treatment, counseling is important for ensuring that patients adhere to their treatment regimen.

Why is Counseling important in TB treatment?

TB treatment involves taking medication for a long period of time, often for six months or more. Some patients may experience side effects from the medication, which can make them feel uncomfortable and may lead them to stop taking their medication. Counseling can help patients to understand the importance of completing their treatment and can provide them with strategies for managing side effects.

What are the benefits of Counseling?

Counseling can help patients to:

- Understand the importance of completing their TB treatment
- Manage side effects from medication
- Develop coping mechanisms for dealing with the stress of TB
- Build self-confidence and motivation to complete treatment

Who can provide Counseling?

Counseling can be provided by a variety of healthcare professionals, including doctors, nurses, and social workers.

If you are a TB patient, it is important to talk to your healthcare provider about counseling services.

Completely curable

100% can be cured. It is necessary to tell what side effects can occur if the treatment is done incompletely.

Important points:

- If the medicine is taken as per the protocol, it will be 100% curable.
- Do not let others get it.
- You need to take care of yourself.
- There are small children, pregnant women in your house. How to keep them away from it.

- If the weight decreases even slightly and the medicine starts in the beginning, it is necessary to take it.
- As soon as the medicine starts, confidence comes.
- One day, seeing this, the patient will also get confidence.

Counselling:

- By doing such counselling, if the patient is cured properly, we can tell others that this patient was like this before.
- If the patient is cured properly, we can tell others that this patient was like this before.
- By doing such counseling, if the patient is cured properly, we can tell others that this patient was like this before.

Patient's confidence:

- If the weight increases, he also gets more self-confidence.

In this way, by doing counselling, if the patient is cured properly, we can tell others that this patient was like this before.

"Now, the TB patient is completely cured. Now, they don't need any counselling. This is one benefit. Now we are talking about the change in TB Preventive Therapy, about the change in medicines, let's talk about their availability. What is the situation? What is your experience in your healthcare facility? Are all available? Right now, only the problem of availability has come."
