# Supplementary material for: Factors influencing tuberculosis preventive treatment implementation in India: A hybrid inductive-abductive thematic analysis using implementation framework
Source: PLoS One. 2026 Oct 14;21(10):e0359171. doi: 10.1371/journal.pone.0359171 (PMC13667825; doi:10.1371/journal.pone.0359171)
Supplement: Supplementary file 4 [file pone.0359171.s001.zip › Focus Group Discussions (Adult contacts).zip!/Focus Group Discussions (MO)/FGD_MO_7.pdf]

FGD_MO_7
Date: 02.08.2024

I: Government of India has started about TB prevention guidelines. Have you heard about it?
R7: We can't treat TB directly
I: I am talking about TB preventive guidelines. Have you heard about it? Has any training done
R1: Training done, DOTS are also happening. We do early diagnosis of symptomatic patients.
R4: Mask wearing is not sufficient due to the open cases of TB. Congestion is also one of the problem. Working and crowded places also responsible as patient are available there. So patients should avoid such places. This can spread.
R9: We have to focus on contacts screening. As one person can come in contact with multiple persons like family and others. We have to focus mostly on close contacts. If one of contact came positive then need to trace the contacts of that patient also. I think we have to start prophylactic.
R10: Our screening is 90% and above. We do sputum, x-ray in sputum positive cases also started TPT, takes timely follow-up. Even in relapse cases we do x-ray and sputum as repeat test for prevention as a preventive measure. Diet also provided as a preventive measure.
R1: Social awareness.
R3: After diagnosis we directly send the patient to home, means there is no isolation.
I: What do you do with household contacts?
R5: INH we give, currently Refampicin also gives with INH
I: INH given to all or according to age?
R5: We give INH to those who are in contact, and if their child is in contact, then we give INH by using weight
R10: I think earlier procedure was good as patients were getting isolated. It is not possible now as reduce in cases
I: Does screening happen?
R5: Yes, need to convince relatives as well as patients that come and do X-ray. And if contacts are negative, we give Prophylactic INH.
I: Obviously counselling must be for them. What tests do you perform?
R1: Sputum, chest X-rays
I: Do all contacts do sputum and chest X-rays?
R1: Contacts who are sputum positive can do a Chest X-ray. Sputum we do for all.
R3: Patients are of drug sensitive and MDR TB. We give INH to drug sensitive patients and for MDR TB we give drugs.
I: Will go step by step.
R1: No sputum for paediatric age group, only X-ray. Sputum not possible.

I: Does it happen 90% after doing counselling?
R10: We do counselling at our level. Most of the time they agree and if we tell them that it's a curable disease, then they also agree.
I: Have you had TPT training?
R6,2: Attended online and offline. Online at the time of COVID.
R8,9: Not done
R3: I joined recently, but there is no training yet.
I: But you learnt. Have you received a portion of the population or the complete population?
R6: Complete population.
R5: Yes
R2: We received the target for sputum. If the population is 1 lakh, then 3600 sputum can be done.
R1: It depends upon one lakh population, if population is area wise then 3400 suspected have to do sputum.
I: For every case, is there any test other than sputum and X-ray?
R1: CBNAAT we do?
I: It is done where?
R10: Balasaheb TU
R6: Sutar hospital, KH also do
I: Are any experts available?
R1: Technicians are there.
I: What percentage of CBNAAT?
R10: MDR contacts do CBNAAT.
R8: Sputum and X-ray we do, if both positive goes for CBNAAT.
I: Contraindications?
R: No
R2: 1st we do a sputum test, then X-ray, and then INH. If the patient has Peripheral TB we avoid it. Also, we did not give INH to heavy drinkers or those who are on ART.
I: Do you follow guidelines?
R10: We usually follow guidelines.
I: What do you do for contacts of MDR TB?
R2: If the patient has pulmonary TB, we provide INH. If the patient is INH-resistant, then we give Rifampicin.
I(asking others): Did you observe such a case? What did you do in this? Among the patient's contacts, were there any pregnant women?
R2: We give INH, as it is not contradictory for pregnant women.
I: INH availability
R: It is available, no shortage

I: For how many months has it been given to contacts?
R2: 3HP for three months and INH for six months.
CTO: 3HP once a week, 12 doses required for three months. A daily dose of INH is needed; sometimes they forget to take it. Patient feels like we had TB, so why take these? Private practitioners are reluctant to provide a single dose on the field. They prefer for patients. If a patient is MDI resistant and if it is negative then in that case then after doing IGRA, INH can be given. Without doing IGRA, if INH is provided it may develop resistance.
I: Has this all been done in training?
I: Drug availability for contacts. Also you said for three months, then what do you think about how many days contacts take? Have you observed any complaints from them or any home visits?
R10: Home visits happen.
I: Percentage of complaints? How many packets were found empty.
R10: 60-70%
R2: 70%
CTO: Axshya and NGO had a work for home visits for the contacts for patients who were positive. Also they do follow-up from enrollment. Three visits were compulsory. They do screening in 1st visit after that they send patients to nearby PHC. Sputum and X-ray were done for them, if found negative then they follow INH/3HP. Mostly we were providing 6H at that time. It is done with 70% people. In some public sector we started INH 100%. Patient from private was told that a follow-up call was received, people came for home visits. Patients usually discuss with each other and say no need to take such medication and all. So we asked private practitioner for their helpful pattern.
I: Has any training been done for a private practitioner?
CTO: In three years, training was done twice. In that Medicine and chest medicine, 70-80% of practitioners were trained. Also taken training for staff who come under Nikshaya for self….. Data from patients has been uploaded on Nikshaya portal and provides recent guidelines to them. National speakers also have asked them about how's going in private practices. Bharati Medical College and Navale Medical College were opposed for TPT. 10% practitioners were ready and 90% were not. Patients from private did not come to us for TPT. The government mostly came.
I: Does private practices help related to TPT? Or small units? Any experience?
R: Not much
CTO: Private did not interfere with us as we have a field, they go directly.
R6: Not for TPT. Private is for basic not for TPT.
R1: There is reluctance also has social stigma. They treat their patient only.
I: Opinion about TPT.
R2: If latent TB came positive then it should be given without doing sputum and microscopy.

I: What about IGRA?
R2: Not in Government.
I: Is TPT useful or not? Any challenges/strategies? Is it possible to eliminate TB till 2025.
R2: Duration can be reduced from six months to three months.
I: Is any infection, side-effect, mentioned or monitored?
R1: If patient is symptomatic then LFT
I(asking to R8): What do you think, you are new here?
R8: Duration is a major problem, in that convincing a patient for six months is quite difficult. If we TPT can be given at contact screening then also it is difficult as I think.
R9: Isolation can be done.
R10: Capacity in Sassoon….diet is useful.
I: Does the patient take a diet or share it with others?
R1: Shares equally
CTO: Malnutrition is a major cause.
R7: Those who didn't get proper diet they mostly found ill
I: TPT supply
R1 and 2: No any problem
R10: Contacts come to collect
R2: Patient or contact come and collects for all
I: For how many months you provide?
R2: For one month
I: Do they come again to collect after one month?
R: Yes
I: Is any case found with HIV, TB co-infection? And what did you do with them?
R10: Refer to sassoon
I: For contacts? Have you done investigations?
R: yes
R10: 40 ANC….now hardly
I: What do you think will TB be eliminated by 2025?
R7: No
R5: Awareness will have to increase, overcrowding can be reduced, use of mask can prevent it.
R7: Pollution can be reduced
I: MDR proportion?
R2: Adherence should have low, treatment duration should be low.
I: What strategies should be followed, what do you think?
R1: Hammering for TPT
R7: BCG
R3: Complicated

CTO: BCG immunity reduces after 8-10 years. Hardly 5-10% of the population has taken BCG from private.
I(Asking R3): What strategies should be followed?
R3: Avoid over crowding
R1: Awareness
R7: There is a daily March (Morcha) happens.
R5: If we ask them to use a mask, they feel bad, and stigma arrives.
R10: Complete vegetarian
R3: Follow-up
R2: Addiction is the cause. They did not follow medicine.
I: MDR percentage
R: In PMC 10%
I: How much is the compliance?
R: Non-compliance 2-3%
CTO: Those 2-3% are beggars or who don't have houses. After diagnosis they get treatment. They don't get food. After admission they follow till 15-20 days or for one month. For them, the Government does not have any policy that they get such people at one place and start one or two Service centers till completion of followup duration or if possible to provide nutrition. So that it cannot spread and also they will cure. In between we experienced that if we get a patient, after sometime it goes lost to follow-up and after a certain period team visitors get that patient. The team visitors come with the patient. They again do test and counselling of that patient and give treatment. This requires 2-3 years.
R10: Spitting is a major problem.
R7: Smoking
R11: Overcrowding and gentle hygiene
I(Asking to CTO): Sir, what do you think apart from strategies what we can do to prevent TB or any challenges.
CTO: We can do that which is possible.
R7: Overcrowding
I: Is TPT useful or not to eliminate TB?
R10: Yes, it is.
R3: Not for everyone. Private did not provide.
R8-9: It it can be
I: Any other point
CTO: Focus on TB prevention to eliminate TB till 2025. We try 100% that INH, 3HP should be compulsory atleast who are ready. It should be 50% private and 50% government. 100% TPT provided to those who are actually ready to take it so that it feels like their family get ill. So that it is possible to eliminate TB till 2030. Our ASHA are doing regular surveys and try to get patient

by doing diagnosis. Maharashtra does a survey for active case finding twice a year. In PMC slum area 350 ASHA do house to house surveys. In that TB templates are distributed asks them for symptoms related to TB. If found symptomatic then sputum test performs and on the spot has been taken place. Then it is sent to the PMC. It takes place twice a year for high risk patients who stay in slum area.Everything has been mentioned in the pamphlet. Extrapulmonary samples also taken. Not all family members are found at one time and one place, so it takes place in morning, afternoon and evening or messages also circulated with them that they should be available in the week and so on. If pamphlets are stayed for a month so that 70-80 people read that and help to resist. Monitoring also takes place for house to house surveys. For everyday there is a target set for ASHA for 40 people. For this close monitoring also happens. One supervisor also provided for one team. The government has provided them formats for data collection. They get remuneration for this work. If they get success to diagnose the patient then they get Rs.500 each. They get motivated before going to the field. Training also happens for them so that the quality of the survey will increase. Only asking that do you have TB will not give output. So that they are trained to ask five symptoms to each person so 2-3 persons staying in the family will also get the same message. Supervisors calls also happen from time to time. Areas have been distributed amongst Medical officers. If the survey starts at 10 AM then the team has to stay on the field at least till 1pm. Revisits also happen if homes are closed.

I: Experience and response of households.

R10: ASHAs always stay near to them, so they are familiar with each other and talk good.

CTO: ASHAs asks them to meet at selected areas. 150-200 patients found yearly. Continuous Monitoring happens for 30-40. They had that track that, which patient did not came to collect the medicine. ASHAs contact such patients and request them to come and collect the medicines. If possible they directly go to patients' homes.

I: Any other points you want to discuss

R1: Continuous and sufficient amount of drug supply is lower.

CTO: Drugs are provided through the central government. Last year two drugs for NDR were not received. It gets purchased through PHC but the cost is quite high so it will take time till 2-3 months.

I: Thank you all
