# Supplementary material for: Factors influencing tuberculosis preventive treatment implementation in India: A hybrid inductive-abductive thematic analysis using implementation framework
Source: PLoS One. 2026 Oct 14;21(10):e0359171. doi: 10.1371/journal.pone.0359171 (PMC13667825; doi:10.1371/journal.pone.0359171)
Supplement: Supplementary file 4 [file pone.0359171.s001.zip › Focus Group Discussions (Adult contacts).zip!/Focus Group Discussions (MO)/FGD_MO_8.pdf]

FGD_MO_8
Date: 05.06.24

I: Do you know about TB prevention guidelines, challenges for implementation?
R1: In TPT isoniazid was from start now the rifapentine has started. We give TPT pulmonary TB household contacts.

R2: We do not provide TPT 3HP to the age group under 2 years but currently due to shortage we are providing directly to the age group above 14 years.

I(asking to R3): What do you want to say regarding this point?

R3: We give INH as a preventive but now I got to know about rifapentine for the age group above and below 14.

I(asking to others): Anyone wants to add anything or any other points related to guidelines?

R4: That is sufficient

R5: First CBNAAT for drug sensitivity

R6: TB is a communicable disease. There is a possibility for contacts and family members to get affected so TPT should start. Before 2021 we were given TPT and INH individually per kg weight to children living in patients home. After 2021 we started giving TPT to adults also who were living in the same house. IF we found some symptoms in contacts then we refer contact to do sputum for CBNAAT, also did X -rays and if bacteria is found then it goes for treatment. We did an IGRA test also, and if the patient comes positive and TPT started. After starting TPT, chances of TB decrease.

I(asking to R7): What do you want to say regarding this point?

R7: 1st we do IGRA test, if results come positive then it we provide INH

I: Percent wise contact

R: 60-70%. Pregnancy is not contraindicated

I: How much contact tracing?

R: 70%

I: If a patient comes positive then we trace his/her household or other work-related persons who regularly comes with patient contact and selects 70-80% of them. Sputum and X-ray we do the same contacts.

I(asking to another R): How about your area?

R: Survey is must for all contacts. In those who come positive that contact undergoes treatment and follow-up is must.

I: Utilization of drugs?

R: 60-70%

R: We gave the regimen only as a daily regimen for 6 months. After that, we started a new regimen, i.e., 3 HPM (after 2021) for household contacts who tested IGRA positive. The dose is for those above 14 years for 3 months (once a week), and compliance has increased.

I: Do you want to add anything in these guidelines? Have you undergone any training related to guidelines?

R: Yes

I: You all have undergone

R6: Updates every time

R2: There is contraindication in pregnancy but we avoid

I: Any other points from those guidelines which you know? All have talked about household contacts and algorithm

R: In HIV we are going to start INH therapy for six months

I: We saw about guidelines, now we will discuss about its implementation that how you follow and actually implements

R: We visit household contacts of Pulmonary TB patient and check for symptoms. If symptoms are found then we go for an IGRA test. IGRA positive get treatment. For below 5 years we prefer TPT. Recovery rate is 80-90%

I: Does any counselling happen for treatment?

R: Yes, we personally do it and THO does.

I: How and what?

R: We generally do like 'how it occurs, it is communicable, also ask them that you might suffer. After starting medicines, the bacterial load decreases. Perhaps it may be found in the body, if it is then it can be reduced between 10-12 days. In that case to prevent infection, medicines will be recommended for 6 months and it is a must. It may show some side effects.' We ask adults not to consume alcohol. We do screening for symptomatic patients. Then IGRA does and the test comes positive then we provide 3HP to the patient. Below 14 years INH therapy we start. It also start for pregnant female

I: Any one?

R: Most people fear that their family will be affected by this, so counseling is a must. Use of mask, hand washing and isolation may prevent. We ask them to help patients without any fear. Counseling related diets can be done.

I: Are medicines available everywhere?

R: Yes, Private sector lacks about TPT

I: Challenges in that case.

R: Counseling has to do, step by step

I: We discussed about implementation now we will move toward the challenges you face. What challenges you face by following TPT and challenges at the time of implementation.

R: Most people require counseling multiple times as they thought they are not ill so why to take care. Follow-up is a must. If counselling is done right then people take positively. The patient stays in contact with us. Patients' relatives get TPT. If contact gets ill then counselling for medicines is must.

I: But what are the challenges?

R: If counseling goes perfect then there is no issue

I: If they don't take then what are the challenges you face?

R: If the mother is patient and the child get in contact, in that case problem occurs…………………………….They should take care in home as well as outside. After counseling…….sometimes what happens they don't get the importance and avoid medicines after certain time medicines stop automatically. Most of the times family members talk about irregularity in taking medicines. To handle mentality is essential in such cases.

I: What are the reasons for complaints?

R: Side effects and duration. For TPT patient comes, there is no issue.

I: You said no problems after counseling

R: Most of the people aware about TB, Some of them are suspected or not even in contact, come and ask us to do sputum and X-ray. Also says about ill patients even though they are unaware about pneumonia or anything else. They tries for awareness.

R: When IGRA comes positive……………..Now we have a weekly 3HP dose. Previously it was daily. 3HP BT is easier than earlier. It is easier to say that to take three medicines i.e. 12 doses. But its side effects haven't been found yet as it has only one completed till. Now IGRA availability is challenging

I: Is this available everywhere now?

R: Last year it was available, now it is in VIP…..margine…..gap…..IGRA report if found….if not then it becomes difficult. Repeated counseling

I: Staff availability?

R: Yes, from target population identification, contact tracing, overall throughout available. Drug availability also.

I: Follow-up and monitoring challenges?

R: There is no record. For monitoring ASHAs are there, all have everything……link…….Per patient ASHAs get 2-15 Rupees. It has started now, earlier this facility was not available. If anyone has not done then it shows in review.

I: Side effects from these medicines.

R: Yes, acidity, hyperacidity, vomiting. They get the question that we should take medicine as it comes out through vomiting. In such cases we prescribe antacid for 15 days-1 month then goes stabilize. Counseling and medicine is essential thing.

I: Anything to add. Record keeping and another challenges?

R: Record is on excel so no issue.

I: We have discussed challenges, now do you have any idea how to overcome them?

R: Counseling is the main other than that, follow-up is required to determine whether the patient is taking medicine or not. Previously diet was also an issue; now, Nikshaya has decreased. There are strategies for TB elimination by taking nutritious food.

I: Do you think that TPT can be eliminated by 2025? The world's target is 2030.

R: Diagnosing newly diagnosed cases will be decrease simultaneously.

I: What you think that TB can be eliminated till 2025, now currently its 2024.

R: Proportion will be reduced

I(asking to other): As early as patient patient detection, active case finding is done then it is possible to detect.

R: Patient comes from observation and private to us. In that cycle, spread is more. We have to do sensitization in private practices. So that detection rate will increase and they don't have to look towards money. They should send patients to us at the earliest. The patient spreads everywhere and then comes to us.

I: Anyone want to share your experience?

R: Most of the patients came like this as they spread everywhere. We found it by checking their histories that they 1st visited to private and now came to us. Most of them start their treatment from private. Once they got some information then came to us. This middle condition that patient came first to private and then government, if possible to reduce then it will be more beneficial.

I: Any more?

R: If a patient informs a private practitioner then they also can manage contact tracing and start TPT. Private practitioners should also counsel them that they will send someone for contact tracing and provide medicine to 10 persons to prevent from TB. This will be more beneficial to us for coordination. Private practitioners can also provide such medications to that patient. They notify and inform them to visit us and patients follow it. If private directly reaches us then it goes easier for the government.

I: Anyone?
R: Complaints increased after 3HP weekly doses. The percentage of entries in Nishaya can be increased.

R: Will start TPT, vaccination early detection and treatment will work. Early detection and early treatment can prevent spread and proportion of TB will be reduced. We thought that in urbanisation TB is found only in poor people but it's wrong. We saw in urban area that, it is found in IT persons. It happens due to their stress factor or diet system. Hence you belong to a rich background but still you will have to follow diet, exercise. As the main reason for the TB is immune system. If immunity is strong, it can prevent such a situation. So we can focus on developing immunity status and also provide knowledge and health education to society. This is the important aspect to reduce TB, only medication cant do everything.

I: Thank you all of you that you all participated here and thanks for your valuable time.
